# Synthesis, Leishmanicidal, Trypanocidal, Antiproliferative Assay and Apoptotic Induction of (2-Phenoxypyridin-3-yl)naphthalene-1(2*H*)-one Derivatives

**DOI:** 10.3390/molecules27175626

**Published:** 2022-08-31

**Authors:** Zuleima Blanco, Esteban Fernandez-Moreira, Michael R. Mijares, Carmen Celis, Gricelis Martínez, Juan B. De Sanctis, Soňa Gurská, Petr Džubák, Marián Hajdůch, Ali Mijoba, Yael García, Xenón Serrano, Nahum Herrera, Jhonny Correa-Abril, Yonathan Parra, Jorge Ángel, Hegira Ramírez, Jaime E. Charris

**Affiliations:** 1 Organic Synthesis Laboratory, Faculty of Pharmacy, Central University of Venezuela, Los Chaguaramos 1041-A, Caracas 47206, Venezuela; 2 Escuela de Medicina, Universidad Espíritu Santo, Samborondón 092301, Ecuador; 3 Biotechnology Unit, Faculty of Pharmacy, Central University of Venezuela, Los Chaguaramos 1041-A, Caracas 47206, Venezuela; 4 Institute of Immunology, Faculty of Medicine, Central University of Venezuela, Los Chaguaramos 1050-A, Caracas 50109, Venezuela; 5 Institute of Molecular and Translational Medicine, Faculty of Medicine, Palacky University, Hněvotínská 1333/5, 77900 Olomouc, Czech Republic; 6 Laboratory of Biology and Chemotherapy of Tropical Parasitosis of the Foundation Institute for Advanced Studies (IDEA) Health Area, Caracas 1080, Venezuela; 7 Grupo de Investigación en Moléculas y Materiales Funcionales (MoléMater), Dirección de Investigación, Universidad Central del Ecuador, Jerónimo Leiton s/n y Gilberto Gatto Sobral, Quito 170521, Ecuador; 8 Laboratorio de Síntesis Orgánica y Diseño de Fármacos, Department de Química, Facultad Experimental de Ciencias, Universidad del Zulia, Maracaibo 4001, Venezuela; 9 Universidad ECOTEC, Km. 13.5 Vía Samborondón, Guayaquil 092302, Ecuador

**Keywords:** *Leishmaniasis*, *Trypanosoma cruzi*, cancer, apoptosis, chalcone

## Abstract

The coexistence of leishmaniasis, Chagas disease, and neoplasia in endemic areas has been extensively documented. The use of common drugs in the treatment of these pathologies invites us to search for new molecules with these characteristics. In this research, we report 16 synthetic chalcone derivatives that were investigated for leishmanicidal and trypanocidal activities as well as for antiproliferative potential on eight human cancers and two nontumor cell lines. The final compounds **8**–**23** were obtained using the classical base-catalyzed Claisen–Schmidt condensation. The most potent compounds as parasiticidal were found to be **22** and **23**, while compounds **18** and **22** showed the best antiproliferative activity and therapeutic index against CCRF-CEM, K562, A549, and U2OS cancer cell lines and non-toxic VERO, BMDM, MRC-5, and BJ cells. In the case of K562 and the corresponding drug-resistant K562-TAX cell lines, the antiproliferative activity has shown a more significant difference for compound **19** having 10.3 times higher activity against the K562-TAX than K562 cell line. Flow cytometry analysis using K562 and A549 cell lines cultured with compounds **18** and **22** confirmed the induction of apoptosis in treated cells after 24 h. Based on the structural analysis, these chalcones represent new compounds potentially useful for *Leishmania*, *Trypanosoma* *cruzi*, and some cancer treatments.

## 1. Introduction

Leishmaniasis is a group of diseases caused by protozoan parasites from more than 20 *Leishmania* species. These parasites are transmitted to humans by the bite of an infected female phlebotomine sandfly. There are three main forms of the disease: cutaneous leishmaniasis (CL), visceral leishmaniasis (VL), also known as kala-azar, and mucocutaneous leishmaniasis (MCL). Approximately 0.9–1.6 million new cases are reported and the mortality rate of the disease varies from 20,000 to 30,000 cases per year. The most common form of human leishmaniasis is CL, which leads to 600,000 to 1 million new infections worldwide annually [1]. The clinical symptoms of CL are mostly restricted to the skin lesions or mucosal affectations for MCL. However, VL is characterized by severe organic symptoms and might lead to death [2,3].

Chagas disease (CD) is caused by *Trypanosoma cruzi*, a hemoflagellate parasite that is transmitted through various species of hematophagous reduviid insects (kissing bugs) mainly in endemic areas, also known as American trypanosomiasis, which is broadly dispersed in Latin America and the Caribbean and affects between 6 and 7 million people and at least 12,000 die each year [4,5,6]. The infection is classified as a neglected tropical disease and related to poor populations in tropical and subtropical regions, although it has been spread to non-endemic areas in Europe, the USA, and Japan [7]. It is a multisystemic disorder that can affect the cardiovascular, digestive, and central nervous systems. Other routes of transmission include transfusion, oral, congenital, organ transplantation, and laboratory accidents [6].

Cancer is the second major cause of death globally after coronary artery disease. In 2018, there were an estimated 18.1 million new cases and 9.6 million deaths from cancer, data registered by the World Health Organization [8]. Cancer is a group of diseases involving abnormal growth of cells, which tend to proliferate in an uncontrolled way. It can affect almost any tissue in the body [9]. The clinical manifestations of cancers are wide-ranging and immunosuppression is a critical side-effect to be considered during the management of cancers [10].

Cancer may be induced by many environmental and physiological conditions, infections are also a risk factor, mainly those caused by bacteria, viruses, and parasites that have been recognized for years to be associated with human carcinogenicity [11,12,13,14,15,16]. The coexistence of leishmaniasis, CD, and neoplasia in human and animal models has been highlighted in previous studies [17,18]. For instance, failures at the epigenetic level to maintain the integrity of chromosomes is one contributing factor in neoplasia and *Leishmania* parasites also modulate and destabilize the host chromatin structure leading to potential changes in relevant immune-related genes and responses [19,20,21]. Moreover, it was shown that infected individuals with severely compromised immune systems, such as cancer patients, transplant recipients, and HIV-positive patients, were at risk for CD reactivation [22,23,24,25,26,27].

A limited number of drugs against leishmaniasis are available for treatment, and the recommended first-line therapies for leishmaniasis include pentavalent antimony compounds, such as sodium stibogluconate and meglumine antimoniate. The second-line treatments include pentamidine and amphotericin B. The oral administration of miltefosine has been used for the treatment of VL in some countries. However, the clinical use of these drugs is accompanied by several disadvantages, such as toxicity, high costs, prolonged treatment, and parenteral or intralesional routes of administration [28,29,30,31,32].

Benznidazole (Bnz, Rochagan™) and nifurtimox (Nfx, Lampit™) are the nitro drugs of choice for the treatment of *T. cruzi* infection. These drugs, introduced into clinical therapy over six decades ago, are effective in inducing cures in the early stages of infection, but the benefit of their administration in the chronic phase is limited due to variable efficacy [33]. In addition, the treatment is long and may lead to several adverse reactions that compromise its continuation [34]. Therefore, innovative models of drug research and development are needed. A logical alternative to combination therapy is the development of multi-target-directed ligands, such as chalcones, that are single small molecules able to hit multiple vital targets in parasites’ metabolic pathways [35,36,37].

Chalcones are vital flavonoid compounds derived from synthetic and natural products that belong to the family of flavonoids and isoflavonoids. Chemically, they consist of an open chain in which the two aromatic rings are joined by a three-carbon α,β-unsaturated carbonyl system. The wide variety of biological and pharmacological activities reported for these compounds include antioxidant, anticancer, anti-Alzheimer’s, anti-inflammatory, immunomodulatory, antiulcer, antibacterial, antimicrobial, immunosuppressive, tyrosinase inhibitor, estrogenic, as well as anti protozoan activity, including trypanocidal, leishmanicidal, and antimalarial [38,39,40].

**Figure 1:**
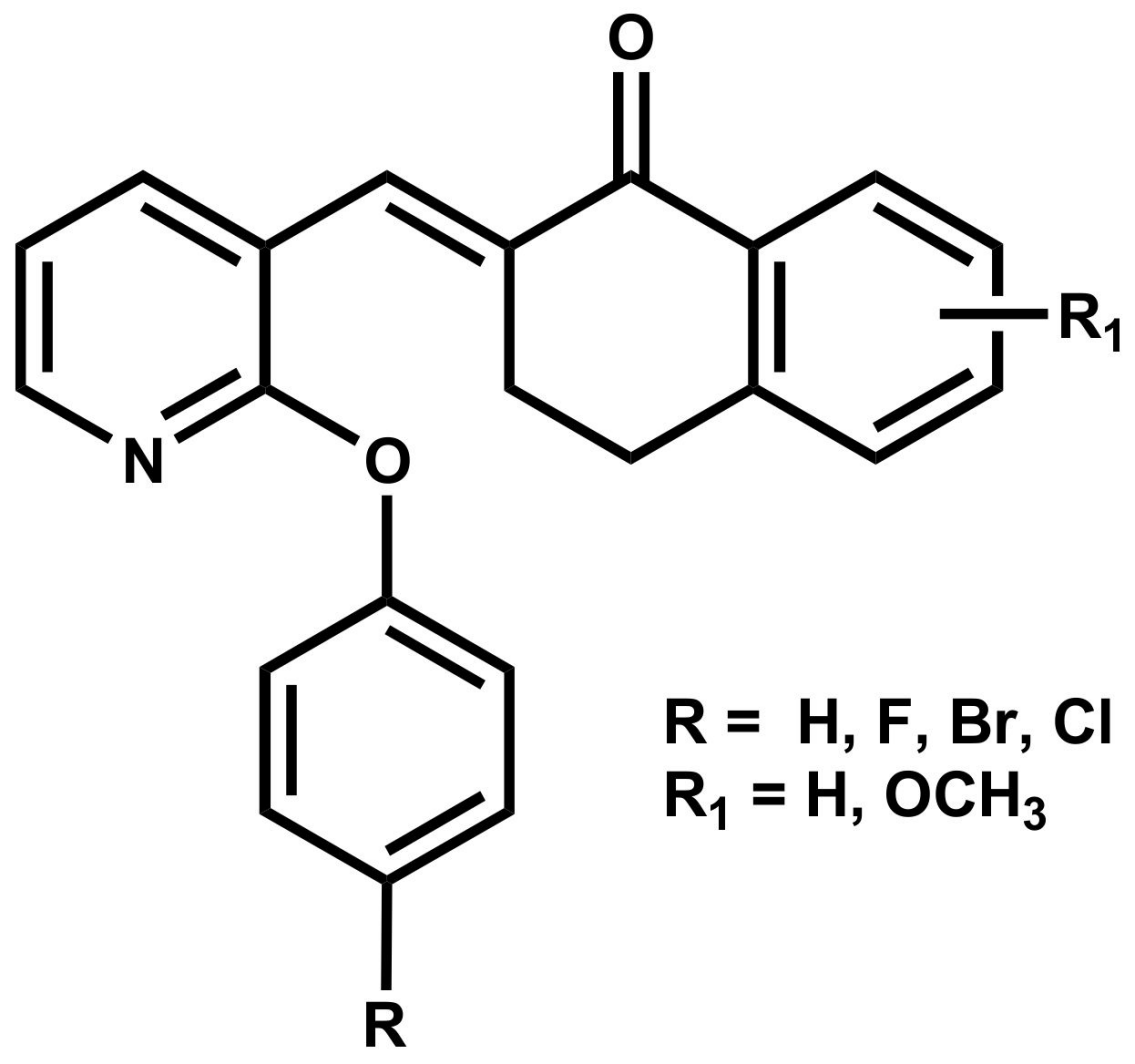
Structures of chalcone derivatives **8**–**23**.

In cancer, several parasitology research approaches have inspired the development of new anticancer drugs, for example, chloroquine, artemisinin, mebendazole, and vice versa miltefosine, camptothecin, imatinib, tipifarnib, tamoxifen, podophyllotoxin, paclitaxel [41,42,43,44,45]. Accordingly, we designed a small library of 16 chalcones derived from (2-phenoxypyridin-3-yl)methylene]naphthalen-1(2*H*)-one **8**–**23** (Figure 1), which aimed to explore and optimize its antiparasitic activity. From a wider perspective, this small library was also evaluated for its antitumor potential. Indeed, there is ample precedence of related chalcones exerting significant antileishmanial, antichagasic, and anticancer activities [38,39,46]. Accordingly, we will discuss below the antiparasitic and antitumor effects of compounds **8**–**23**, with a special focus on apoptotic activity as a possible mechanism of action.

## 2. Results and Discussion

### 2.1. Chemistry

**Scheme 1:**
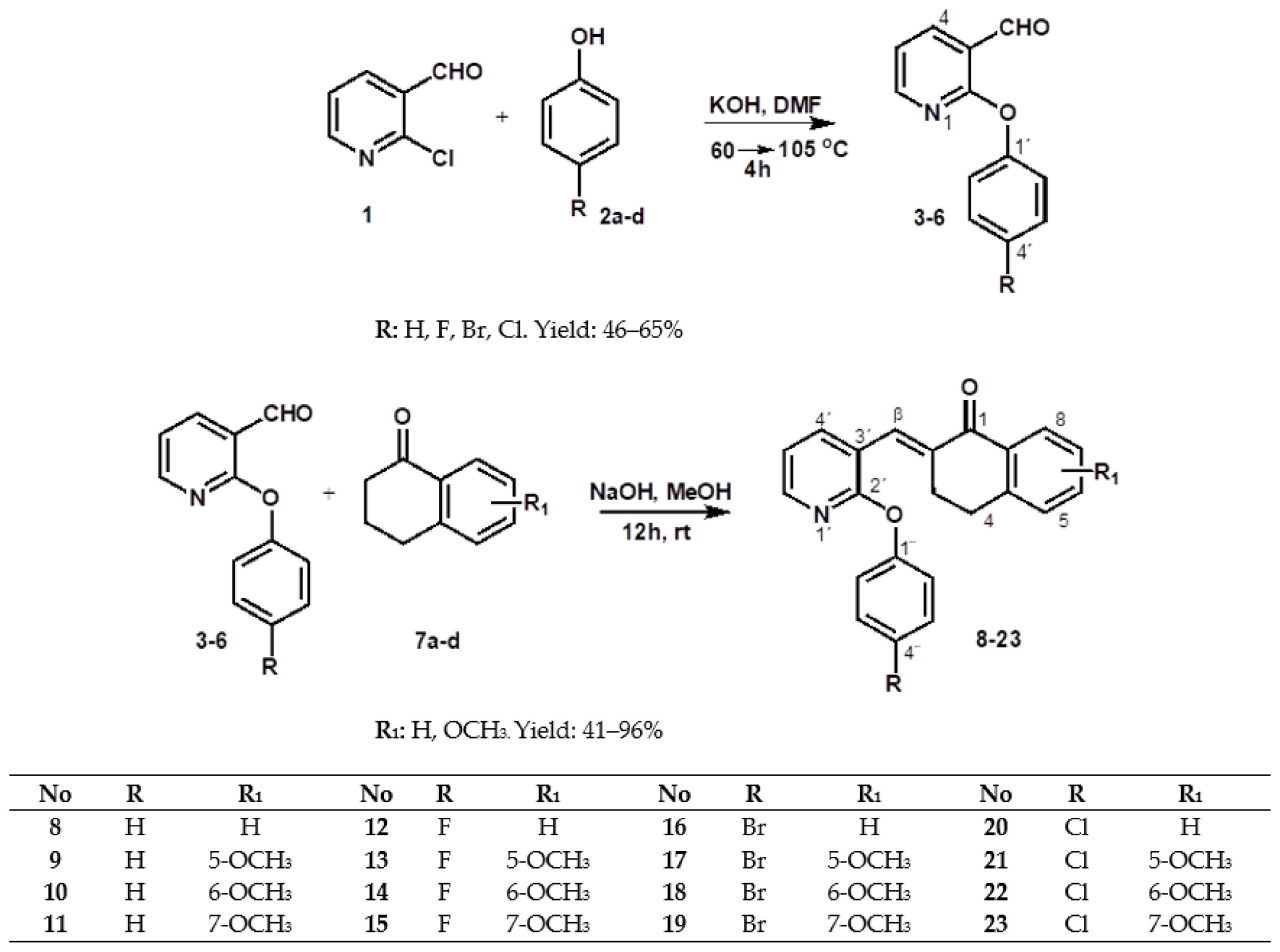
Synthesis of (*E*)-3,4-dihydro-2-[(2-phenoxypyridin-3-yl)methylene]naphthalen-1(2*H*)-one analogs **8**–**23**.

Our synthetic plan towards (2-phenoxypyridin-3-yl)naphthalen-1(2*H*)-one was based on a two-step, first procedure-attachment of phenol to pyridine ring at position 2, and finally the classical Claisen–Schmidt condensation base-catalyzed in methanol at room temperature (Scheme 1). This reaction has been widely used for the synthesis of chalcones, typically with good to excellent yields. In the required key starting materials, 2-phenoxypyridine-3-carbaldehyde **3**–**6** were prepared using a modification of the procedure previously reported [47], the reaction of 2-chloro-3-pyridinecarboxaldehyde **1** with phenols **2a**–**d** in the presence of anhydrous potassium hydroxide in dry *N*,*N*-dimethylformamide at 60 °C in 46–65% yields. The structures of **3**–**6** were elucidated by infrared (IR), ^1^H-, and ^13^C-NMR spectral analyses. The IR spectra of the compounds show one characteristic intense stretching band between 1686 and 1680 cm^−1^, confirming the presence of C=O. In the ^1^H-NMR spectra, the characteristic chemical shift of the proton of aldehyde was found around 10.51–10.54 ppm as a singlet(s), and the pyridine protons 4–6 around 7.81, 8.23, and 8.32 ppm as a double doublet (dd) with a *J* = 7.8, 7.2, and 1.8 Hz, respectively. The structures were confirmed further by ^13^C-NMR spectra, the chemical shift of the C=O was found to be a signal between 188.8 and 188.5 ppm, and the pyridine carbons 4–6 as a signal around 119, 138, and 153 ppm, respectively. The final compounds **8**–**23** were synthesized through aldol condensation of Claisen–Schmidt between compounds **3**–**6** and several methoxys substituted 1-tetralones **7a**–**d**, using potassium hydroxide in methanol at room temperature. These conditions were found to be satisfactory for the synthesis with a yield between 41 and 96%. Theoretically, *E* and *Z* geometric isomers can be equally formed during the reaction. However, only (*E*) isomers were obtained, which were confirmed by the singlet in the ^1^H-NMR spectra between 7.87 and 7.95 assigned to the protons in the Hβ position. Perhaps due to diamagnetic anisotropy of carbonyl group, where vinyl proton of (*E*) isomer gives a signal with a greater chemical shift than the vinyl proton of (*Z*) isomer, an observation that has been noted previously [48,49]. The aliphatic signals expected at upfield shifts were found as t from 2.91 to 3.06 ppm. Based on their chemical shifts, multiplicity and *J* were assigned the rest of the protons. The ^13^C-NMR spectrum of the same compounds exhibits signals between 120 and 127 ppm for Cβ, 135–139 ppm for Cα, and 186–187 ppm for C=O, which were also confirmed by DEPT 135° (distortionless enhancement by polarization transfer) (see Supplementary Materials, NMR spectra). The infrared (IR) spectra of the compounds show one characteristic intense stretching band between 1660 and 1699 cm^−1^, confirming the presence of α,β unsaturated C=O. The product formation was further substantiated by its mass spectra, all the compounds gave satisfactory elemental analysis.

### 2.2. Biology Assay

#### 2.2.1. Preliminary Antiparasitic Activity: MTT Studies

The obtained de novo chalcones **8**–**23** were evaluated on *L. braziliensis* promastigotes and *T. cruzi* epimastigotes proliferation, and the cytotoxicity assays on mouse bone marrow-derived macrophages (BMDM), and VERO cells derived from the kidney of an African green monkey. The concentration-response data for the most active derivatives were fitted by a nonlinear regression model and the concentration that induces 50% inhibition was calculated as the effective concentration EC_50_ on *L. braziliensis* promastigotes and *T. cruzi* epimastigotes.

In the first screening through MTT assays, we observed that compounds **21**–**23** showed an appreciable biological activity (EC_50_ < 15 μM) on parasite proliferation, and low cytotoxicity against BMDM and VERO cells was observed (EC_50_ > 100 μM). Compounds **8**, **10**, and **19** showed moderate activity (EC_50_ ≤ 60 μM) against *L. braziliensis* promastigotes, while compounds **20** and **21** (EC_50_ < 60 μM) can be considered with moderate activity against *T. cruzi* epimastigotes. A weak antileishmanial activity was observed for compounds **9**, **11**, **12**–**18**, and **20** with values (EC_50_ ≥ 60 μM), while a weak trypanocidal activity was observed for compounds **8**–**19** (Table 1). The three molecules **21**–**23** showed higher activity (9.24 ± 1.00 μM, 12.37 ± 1.15 μM, and 5.69 ± 2.65 μM, respectively), than miltefosine the reference drug (EC_50_ 25 ± 1.40 μM) as leishmanicidal. As trypanocidal compounds, **22** and **23** showed higher activity (5.03 ± 0.49 μM, 4.91 ± 0.98 μM), than benznidazole the reference drug (30.00 ± 0.68 μM). From EC_50_ as leishmanicidal, it was possible to calculate the selectivity index (SI) of compound **23** obtaining (SI 18 and 176) on VERO and BMDM cells, respectively. Meanwhile, miltefosine results in an (SI 5) that represents at least 3.5 to 35.1 times more active against the parasites than on the host cells. The values of SI for compounds **22** and **23** were (**22** SI 20 and 93 for **23** 20 and >204) on VERO and BMDM cells, while benznidazole presents a (SI 4) that represents at least 4.9, 23.3, 5.1, and 50.9 times more actives against the parasites than on the host cells. These results are interesting due miltefosine and benznidazole are the first drugs used for visceral leishmaniasis and Chagas disease treatments, respectively.

An analysis of the structure–activity relationship for the evaluated chalcones **8**–**23** as leishmanicidal and trypanocidal, permitted us to establish the following remarks. Replacement of the chlorine atom **21**–**23** by hydrogen, fluorine, and bromine atom on position four of phenoxy on position two of pyridine, decreases the leishmanicidal and trypanocidal activities. The most notable differences in activity were observed when a chlorine atom on position 4 on phenoxy and a methoxy group on position 7 of the tetralone are present, however, when the methoxy group is on position 5 or 6 a marginal loss of biological activity was observed. The replacement of the methoxy group by a hydrogen atom in the tetralon system represents a significant loss of parasiticidal activity.

#### 2.2.2. Antiproliferative Activity

Compounds **8**–**23** were tested in vitro for their antiproliferative activity on eight cancer cell lines: CCRF-CEM, CEM-DNR, K562, K562-TAX, A549, HCT116, HCT116p53−/−, U2OS, and on the two non-malignant cell lines MRC-5 and BJ. Antiproliferative activities are presented in (Table 2) and are expressed as IC_50_ values. The tested compounds showed IC_50_ values of between 3.91 ± 0.14 µM and more than 50 µM. The acute lymphoblastic leukemia CCRF-CEM cell line was the most sensitive to tested chalcones, particularly compounds **8**, **9**, **11**–**13**, and **15** (IC_50_ in the range of 4.15 ± 0.74 to 9.13 ± 1.18 µM) bearing H or F on phenoxy and H or OMe groups on position 5 or 7 of tetralone, and the compounds **16**, **18**, **20**, and **22** (IC_50_ in the range of 5.23 ± 0.30 to 8.7 ± 2.19 µM) bearing Br or Cl on phenoxy and H or OMe groups on position 6 of tetralone, what implies a correlation between the kind of halogen and position of the OMe group on tetralone and antiproliferative activity. All the compounds were less active against their daunorubicin-resistant CEM-DNR counterparts.

In the case of K562 and the corresponding drug-resistant K562-TAX cell lines, the antiproliferative activities showed a more significant difference for compounds **8**, **19,** and **23** having 2.5, 10.3, and 3.1 times higher antiproliferative activity K562-TAX than K562 cell line (20.0 ± 2.16 µM vs. > 50 µM, 4.17 ± 2.93 µM vs. 42.95 ± 5.1 µM, and 16.05 ± 4.44 µM vs. > 50 µM). On the other hand, compounds **14**, **18**, and **22** were 6.8, 4.1, and 5.5 times higher cytotoxicity against K562 than K562-TAX cell line (5.74 ± 3.2 µM vs. > 39.25 ± 9.37 µM, 4.33 ± 0.19 µM vs. 17.62 ± 7.09 µM, and 3.91 ± 0.14 µM vs. > 21.44 ± 4.08 µM). These results indicate that for the resistance, other mechanisms than P-glycoprotein are responsible, which is common for both cell lines.

Except for compounds **18** and **22** (9.64 ± 1.71 µM, and 9.74 ± 1.28 µM) all other compounds were less active against the human lung adenocarcinoma A549. Antiproliferative activity of compounds **8**–**23** tested against HCT116 and HCT116p53−/− were similar. The most efficient was compound **22** with IC_50_ of (12.95 ± 3.61 µM) in the case of HCT116. Except for compounds **18** and **22** (7.27 ± 0.68 µM, and 8.1 ± 1.71 µM), all other compounds were less active against the human U2OS cell line derived from osteosarcoma. In contrast, no effect was observed against the non-tumor lines BJ and MRC-5 when compounds **8**–**23** were evaluated (IC_50_ > 50 µM).

In general, the compounds were substantially less toxic on non-cancer cell lines MRC-5 and BJ than against the eight cancer cell lines evaluated: CCRF-CEM, CEM-DNR, K562, K562-TAX, A549, HCT116, HCT116p53−/−, U2OS, the favorable therapeutic index (TI) is expressed as the ratio between the average IC_50_ value of non-cancer cell line and the IC_50_ value of a given cancer cell line.

The highest TI value among all tested compounds was observed for compound **8** (CCRF-CEM 12). Compound **22** also showed high TI (CCRF-CEM 9.6), against the same cell line as TI (CCRF-CEM 8.4) was calculated for compound **18**. Compounds **9**, **11**, **12**, **13**, **15, 16**, and **20** are shown against the same cell line a TI (CCRF-CEM 8.2 to 5.5). Three compounds **14**, **18**, and **22** exhibit TI values for the K652 cell line (K562 8.7, 11.5, and 12.8, respectively), **19** was the only compound with good TI against some of the multidrug-resistant cell lines (K562-TAX 12), against the same cell line a TI (K562-TAX 3.1, 2.8, and 2.5) was calculated for compounds **23**, **18**, **15**, respectively. Two compounds **18** and **22** exhibit TI values for the A549 cell line (A549 5.2 and 5.1, respectively). Higher TI values for the U2OS cell line were calculated for compounds **18** and **22** (U2OS 6.9 and 6.2, respectively).

Therefore, we can infer that the antiproliferative effect of this class of compounds is related to the type of halogen atoms and the position of the methoxy group in the tetralone nucleus. The detailed mechanism of action will be the subject of our future investigation.

#### 2.2.3. Annexin V/Propidium Iodide (PI) Labeling

**Figure 2:**
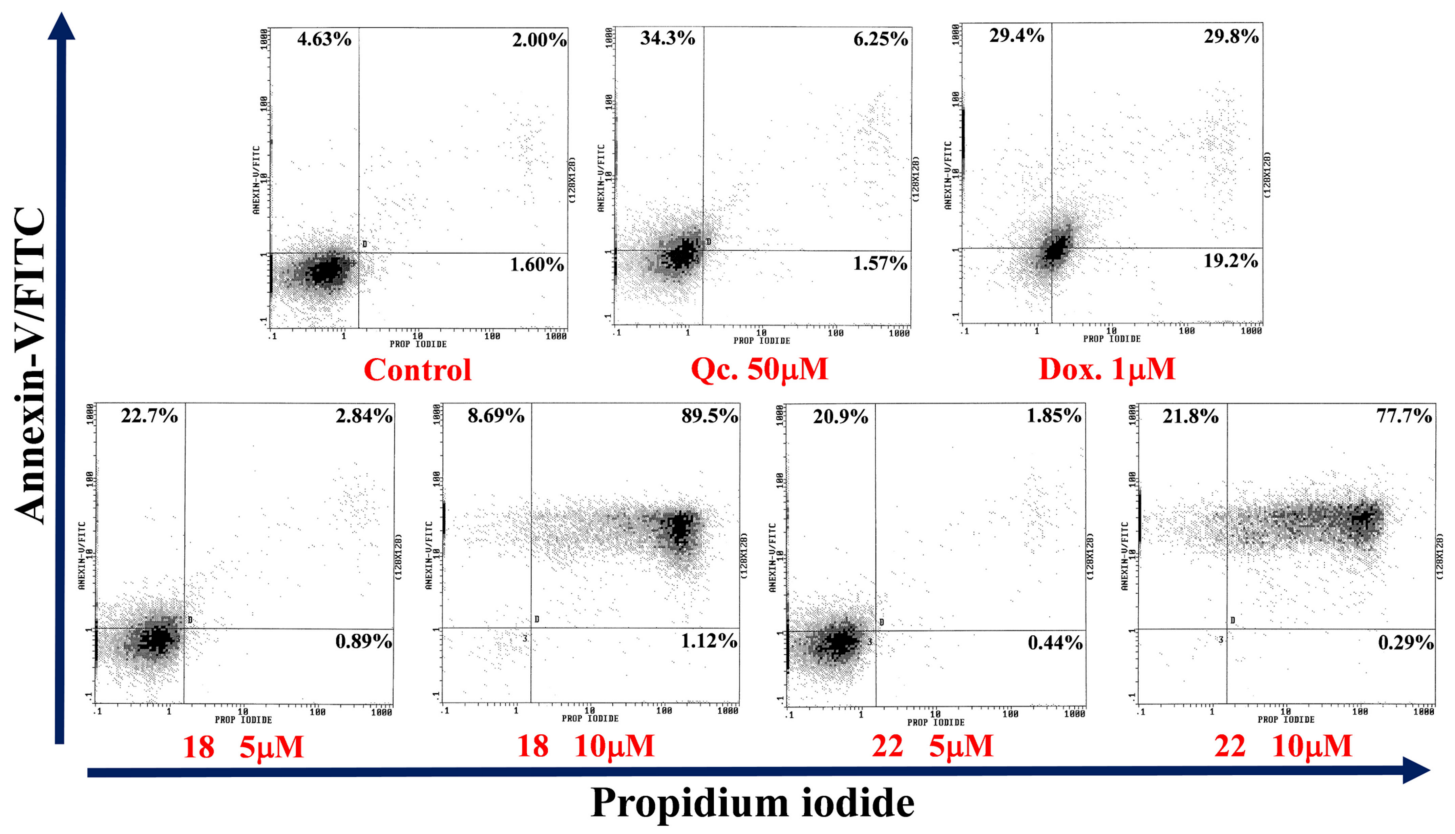
Effect of compounds **18** and **22** on apoptosis and necrosis of human K562 cell line assessed by flow cytometry. Early apoptosis was defined by annexin V-FITC brightness; necrosis expression by PI and late apoptosis by double positiveness. The double plots illustrate the effect of 24 h incubation with compounds **22** and **18** (5 and 10 µM), QC: quercetin (50 μM), Dox: doxorubicin (1 μM), and control.

**Figure 3:**
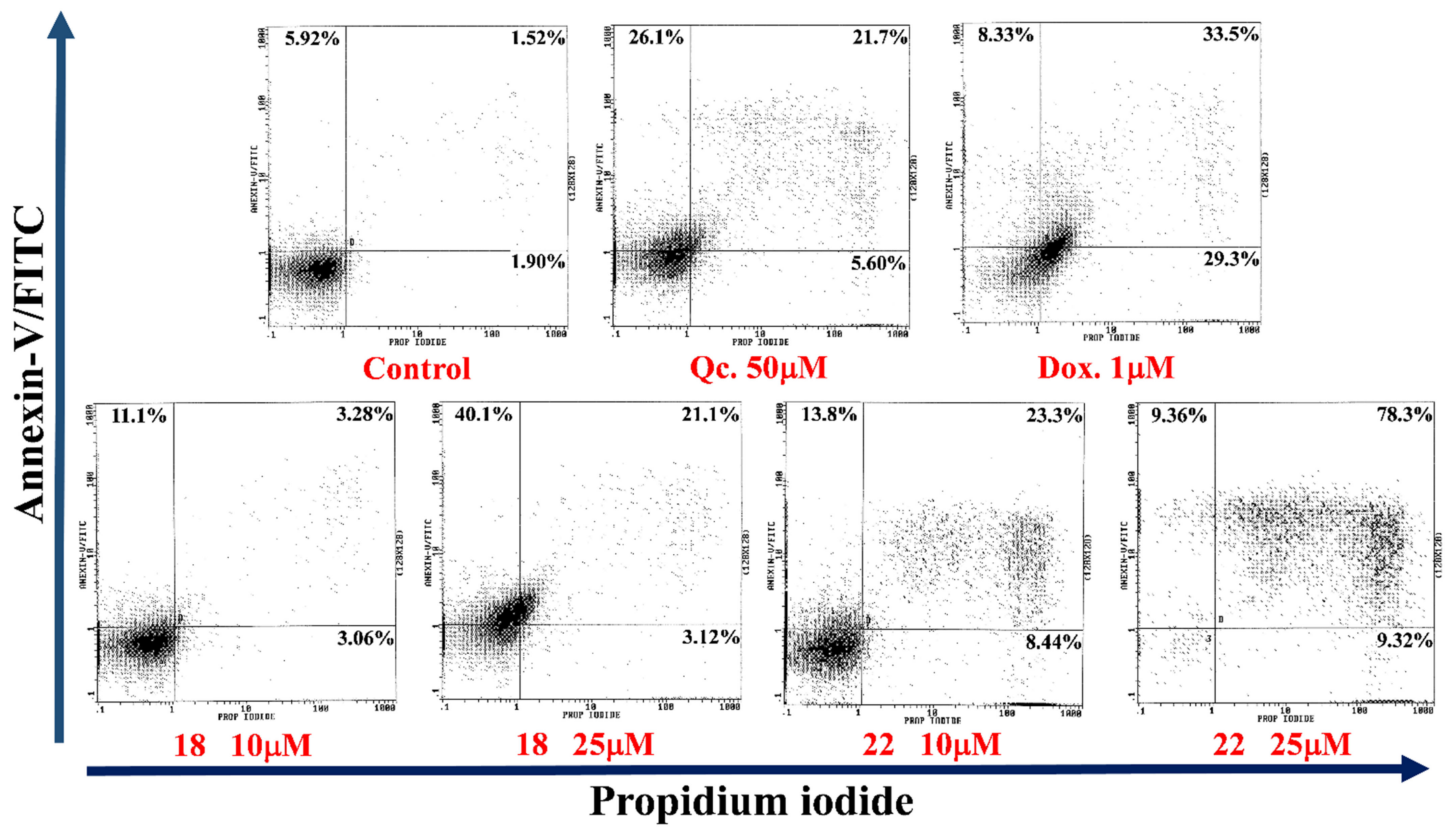
Effect of compounds **18** and **22** on apoptosis and necrosis of human A549 cell line assessed by flow cytometry. Early apoptosis was defined by annexin V-FITC brightness; necrosis expression by PI and late apoptosis by double positiveness. The double plots illustrate the effect of 24 h incubation with compounds **22** and **18** (10 and 25 µM), Qc: quercetin (50 μM), Dox: doxorubicin (1 μM), and control.

Cell death was assessed after 24 h incubation with compounds **18** and **22**. Apoptosis was analyzed by flow cytometry using annexin V-FITC and propidium iodide (PI) [50]. Early apoptosis was defined by annexin V-FITC expression and, necrosis by PI expression, late apoptosis by coexpression of annexin V/PI. Doxorubicin and quercetin were used as positive controls [51,52,53,54,55,56]. The results of a typical flow cytometry analysis of both compounds are illustrated in Figure 2 and Figure 3. An increase in early apoptosis and late apoptosis was observed; necrosis was less predominant. The results are summarized in Tables S1 and S2 (Supplementary Materials). The expression of annexin V was different depending on the compound used.

In the 24 h-treated K562 cell line, the maximum effect was reached at the concentration range of 10 μM. At 10 μM, annexin V-FITC expression was higher than 50% in cells treated with compounds **18** and **22** (10 μM). On the other hand, annexin V-FITC fluorescence augmented significantly when the A549 cell line was treated with compounds **18** and **22**, the maximum effect was observed at 25 μM in the cell line. The maximum effect of doxorubicin was recorded at 1 μM; however, it generated a marked increase in cell necrosis. Tables S1 and S2 show the influence of the compounds on PI expression. It can be concluded that the compounds did not induce necrosis of the cell lines tested.

## 3. Materials and Methods

### 3.1. Chemistry

Reactions were monitored by thin-layer chromatography (TLC) carried out on aluminum sheets precoated with silica gel 60 F254 (Merck KGaA, Darmstadt, Germany). Compounds were visualized with UV light. Column chromatography was performed on Merck silica gel 60 (40–63 µm) as a stationary phase. The ^1^H and ^13^C nuclear magnetic resonance (NMR) spectra were recorded using a Bruker Ascen^TM^ 600 (600 MHz/150.89 MHz) spectrometer (Bruker Bioscience, Billerica, MA, United States) using CDCl_3_ as the solvent, and are reported in ppm downfield from the residual CHCl_3_ (δ 7.25 ppm for ^1^H-NMR and 77.0 ppm for ^13^C-NMR). Spin multiples are given as singlet(s), doublet (d), double doublet (dd), and multiplet (m), where coupling constant (*J*) values were estimated in Hertz. A Thomas^TM^ Hoover Capillary Melting Point Apparatus (Thomas Scientific, Seattle, WA, USA) was used to determine the melting points (mp) and are uncorrected. Infrared (IR) spectra were determined as KBr pellets on a Shimadzu^TM^ IR-470 spectrophotometer (Shimadzu Co., Kyoto, Japan) and are expressed in cm^−1^. A Perkin Elmer^TM^ 2400 CHN elemental analyzer (Perkin Elmer, Inc., Waltham, MA, USA) was used to obtain the elemental analyses, and the results were within ±0.4% of the predicted values. Exact molecular masses were determined on a Agilent 5977E GC/MSD spectrometer (Agilent Technologies, Inc., Santa Clara, CA, USA). Solvents were purchased from different chemical suppliers and were dried and distilled under a nitrogen atmosphere.

#### 3.1.1. General Procedure for the Synthesis and Characterization of Compounds **3**–**6**

Phenol **2a**–**d** (3.0 mmol), powdered KOH (4.0 mmol), and dry *N*,*N*-dimethylformamide 5 mL, were combined in a 20 mL round-bottom flask fitted with a reflux condenser. The mixture was heated to 60 °C and allowed to stir for approximately 2 h. Subsequently, the suitable 2-chloropyridine-3-carbaldehyde **1** (2.0 mmol) was added and the reaction mixture was stirred and heated at 105 °C for 2 h plus. The reaction was quenched by the addition of water 10 mL. The organic phase was washed three times with saturated, aqueous K_2_CO_3_ 5 mL, and then separated and dried with anhydrous Na_2_SO_4_, which was collected by filtration. Solvents were removed under reduced pressure to produce a solid. The product was purified by recrystallization from ethanol:water (3:1).

##### 2-Phenoxypyridine-3-carbaldehyde (**3**)

Beige solid; yield: 56% from ethanol: water; m.p. 88–89 °C [lit. 86–88 °C] [47]; IR (KBr) cm^−1^: 1686, 1577,1427, 1248; ^1^H-NMR (CDCl_3_, 600 MHz) δ ppm: 7.11 (dd, 1H, H_5_, *J*_1_ = 7.8 Hz, *J*_2_ = 7.8 Hz), 7.18 (dd, 2H, H_2′,6′_, *J*_1_ = 8.4 Hz, *J*_2_ = 3.0 Hz), 7.25 (t, 1H, H_4′_, *J* = 8.4), 7.42 (t, 2H, H_3′_,_5′_, *J* = 8.4), 8.23 (dd, 1H, H_4_, *J*_1_ = 7.2 Hz, *J*_2_ = 1.8 Hz), 8.32 (dd, 1H, H_6_, *J*_1_ = 7.2 Hz, *J*_2_ = 1.8 Hz), 10.54 (s, 1H, CHO). ^13^C-NMR (CDCl_3_, 150.89 MHz) δ ppm: 118.9, 119.9, 121.6, 125.4, 129.7, 138.0, 153.0, 153.1, 164.0, 188.8 (CHO). Anal. calcd. for: C_12_H_9_NO_2_: C 72.35, H 4.55, N 7.03; Found C 72.33, H 4.57, N 7.21.

##### 2-(4-Bromophenoxy)pyridine-3-carbaldehyde (**4**)

Cream solid; yield: 65% from ethanol: water; m.p. 77–78 °C; IR (KBr) cm^−1^: 1680, 1585, 1418, 1230; ^1^H-NMR (CDCl_3_, 600 MHz) δ ppm: 7.07 (d, 2H, H_2′,6′_, *J* = 9.0 Hz), 7.13 (dd, 1H, H_5_, *J*_1_ = 7.5 Hz, *J*_2_ = 7.5 Hz), 7.53 (d, 2H, H_3′,5′_, *J* = 9.0 Hz), 8.23 (dd, 1H, H_4_, *J*_1_ = 7.5 Hz, *J*_2_ = 1.8 Hz), 8.31 (dd, 1H, H_6_, *J*_1_ = 7.5 Hz, *J*_2_ = 1.8 Hz), 10.51 (s, 1H, CHO). ^13^C-NMR (CDCl_3_, 150.89 MHz) δ ppm: 118.5, 119.3, 119.5, 123.5, 133.7, 138.3, 152.0, 152.9, 163.6, 188.5 (CHO). Anal. calcd. for: C_12_H_8_BrNO_2_: C 51.83, H 2.90, N 5.04; Found C 51.82, H 2.91, N 5.19.

##### 2-(4-Chlorophenoxy)pyridine-3-carbaldehyde (**5**)

Brown solid; yield: 46% from ethanol: water; m.p. 84–86 °C; IR (KBr) cm^−1^: 1686, 1644, 1565, 1420; ^1^H-NMR (CDCl_3_, 600 MHz) δ ppm: 7.11–7.14 (m, 3H, H_2′,6′,5_), 7.38 (d, 2H, H_3′,5′_, *J* = 9.0 Hz), 8.23 (dd, 1H, H_4_, *J*_1_ = 7.2 Hz, J_2_ = 1.8 Hz), 8.31 (dd, 1H, H_6_, *J*_1_ = 7.2 Hz, *J*_2_ = 1.8 Hz), 10.51 (s, 1H, CHO). ^13^C-NMR (CDCl_3_, 150.89 MHz) δ ppm: 119.4, 119.5, 123.1, 129.0, 130.8, 138.3, 151.4, 153.0, 163.7, 188.5 (CHO). Anal. calcd. for: C_12_H_8_ClNO_2_: C 61.69, H 3.45, N 5.99; Found C 61.73, H 3.49, N 6.23.

##### 2-(4-Fuorophenoxy)pyridine-3-carbaldehyde (**6**)

Brown solid; yield: 57% from ethanol: water; m.p. 76–78 °C; IR (KBr) cm^−1^: 1683, 1578, 1508, 1430; ^1^H-NMR (CDCl_3_, 600 MHz) δ ppm: 7.09–7.15 (m, 5H, H_5,2′,3′,5′,6′_), 8.22 (dd, 1H, H_4_, *J*_1_ = 7.2 Hz, *J*_2_ = 1.8 Hz), 8.31 (dd, 1H, H_6_, *J*_1_ = 7.2 Hz, *J*_2_ = 1.8 Hz), 10.52 (s, 1H, CHO). ^13^C-NMR (CDCl_3_, 150.89 MHz) δ ppm: 116.4 (*J*_C-3′,5′_ = 24.1 Hz), 119.0, 123.1 (*J*_C-2′,6′_ = 7.5 Hz), 138.3, 148.6 (*J*_C-1′_ = 3.0 Hz), 152.9, 160.7 (*J*_C-4′_ = 242.9 Hz), 163.9, 188.5 (CHO). Anal. calcd. for: C_12_H_8_FNO_2_: C 66.36, H 3.71, N 6.45; Found C 66.38, H 3.77, N 6.62.

#### 3.1.2. General Procedure for the Synthesis and Characterization of Compounds **8**–**23**

A mixture of 2-phenoxypyridine-3-carbaldehyde 3–6 (0.14 mmol), tetralone 7a–d respective (0.14 mmol), and sodium hydroxide (0.1 mmol) in methanol (2 mL) was stirred at room temperature by 12 h. The resulting precipitate was collected by filtration, washed with cold water, sodium bisulfite 10% aqueous solution, and diethyl ether. The product was purified by recrystallization from methanol.

##### (*E*)-3,4-Dihydro-2-[(2-phenoxypyridin-3-yl)methylene]naphthalen-1(2*H*)-one (**8**)

Beige solid; yield: 73% from methanol; m.p. 154 °C; IR (KBr) cm^−1^: 1667, 1568, 1408, 1292; ^1^H-NMR (CDCl_3_, 600 MHz) δ ppm: 2.98 (t, 2H, H_3_, *J* = 5.4 Hz), 3.06 (t, 2H, H_4_, *J* = 5.4 Hz), 7.03 (dd, 1H, H_5_, *J*_1_ = 1.2 Hz, J_2_ = 6.0 Hz), 7.12 (dd, 2H, H_2″,6″_, *J*_1_ = 1.8 Hz, *J*_2_ = 7.8 Hz), 7.18 (t, 1H, H_4″_, *J* = 7.8 Hz), 7.24 (dd, 1H, H_5′_, *J*_1_ = 6.0 Hz, *J*_2_ = 6.0 Hz), 7.35 (t, 1H, H_7_, *J* = 6.0 Hz), 7.38 (t, 2H, H_3″,5″_, *J* = 7.8 Hz), 7.48 (t, 1H, H_6_, *J* = 6.0 Hz), 7.69 (dd, 1H, H_4′_, *J*_1_ = 1.8 Hz, *J*_2_ = 7.1 Hz), 7.95 (s, 1H, =CH), 8.12-8.14 (m, 2H, H_8,6′_).^13^C-NMR (CDCl_3_, 150.89 MHz) δ ppm: 27.7, 28.8, 118.0, 120.1, 121.4, 124.7, 127.0, 128.2, 128.3, 129.5, 130.2, 133.2, 133.4, 137.6, 139.3, 143.2, 147.3, 153.8, 161.4, 187.3 (C=O). MS (*m/z*, %): 327 (M+, 1.2), 234 (48), 118 (20), 115 (19), 77 (100). Anal. calcd. for: C_22_H_17_NO_2_: C 80.71, H 5.23, N 4.28; Found C 80.71, H 5.25, N 4.39.

##### (*E*)-3,4-Dihydro-5-methoxy-2-[(2-phenoxypyridin-3-yl)methylene]naphthalen-1(2*H*)-one (**9**)

Yellow solid; yield: 74% from methanol; m.p. 148 °C; IR (KBr) cm^−1^: 1699, 1574, 1468, 1286; ^1^H-NMR (CDCl_3_, 600 MHz) δ ppm: 2.95 (t, 2H, H_3_, *J* = 6.0 Hz), 3.03 (t, 2H, H_4_, *J* = 6.0 Hz), 3.85 (s, 3H, OCH_3_), 7.02-7.03 (m, 2H, H_5′,6_), 7.13 (d, 2H, H_2″,6″_, *J* = 7.8 Hz), 7.18 (t, 1H, H_4″_, *J* = 7.2 Hz), 7.31 (t, 1H, H_7_, *J* = 7.8 Hz), 7.37 (t, 2H, H_3″,5″_, *J* = 7.8 Hz), 7.69 (dd, 1H, H_4′_, *J*_1_ = 1.8 Hz, *J*_2_ = 7.2 Hz), 7.75 (d, 1H, H_8_, *J* = 7.8 Hz), 7.91 (s, 1H, =CH), 8.12 (dd, 1H, H_6′_, *J*_1_ = 1.7 Hz, *J*_2_ = 7.2 Hz). ^13^C-NMR (CDCl_3_, 150.89 MHz) δ ppm: 21.6, 26.9, 55.7, 114.4, 118.0, 119.9, 120.2, 121.4, 124.7, 127.2, 129.5, 129.8, 132.3, 134.2, 137.6, 139.3, 147.3, 153.8, 156.3, 161.5, 187.6 (C=O). MS (*m/z*, %): 264 (52), 148 (17), 115 (11), 77 (100). Anal. calcd. for: C_23_H_19_NO_3_: C 77.29, H 5.36, N 3.92; Found C 77.32, H 5.35, N 4.13.

##### (*E*)-3,4-Dihydro-6-methoxy-2-[(2-phenoxypyridin-3-yl)methylene]naphthalen-1(2*H*)-one (**10**)

Yellow solid; yield: 61% from methanol; m.p. 134 °C; IR (KBr) cm^−1^: 1698, 1590, 1408, 1289; ^1^H-NMR (CDCl_3_, 600 MHz) δ ppm: 2.94 (t, 2H, H_3_, *J* = 6.0 Hz), 3.03 (t, 2H, H_4_, *J* = 6.0 Hz), 3.85 (s, 3H, OCH_3_), 6.69 (d, 1H, H_5_, *J* = 2.4 Hz), 6.86 (dd, 1H, H_7_, *J*_1_ = 3.0 Hz, *J*_2_ = 9.0 Hz), 7.02 (dd, 1H, H_5′_, *J*_1_ = 7.2 Hz, *J*_2_ = 7.2 Hz), 7.12 (d, 2H, H_2″,6″_, *J* = 7.8 Hz), 7.17 (t, 1H, H_4″_, *J* = 7.8 Hz), 7.37 (d, 2H, H_3″,5″_, *J* = 7.8 Hz), 7.67 (dd, 1H, H_4′_, *J*_1_ = 1.7 Hz, *J*_2_ = 7.2 Hz), 7.91 (s, 1H, =CH), 8.10–8.12 (m, 2H, H_6′,8_). ^13^C-NMR (CDCl_3_, 150.89 MHz) δ ppm: 27.7, 29.3, 55.4, 112.3, 113.4, 118.0, 120.3, 121.3, 124.7, 126.8, 129.5, 129.5, 130.8, 137.8, 139.3, 14 5.7, 147.2, 153.8, 161.4, 163.6, 186.2 (C=O). MS (*m/z*, %) 264 (35), 148 (12), 115 (9), 77 (100). Anal. calcd. for: C_23_H_19_NO_3_: C 77.29, H 5.36, N 3.92; Found C 77.30, H 5.39, N 4.09.

##### (*E*)-3,4-Dihydro-7-methoxy-2-[(2-phenoxypyridin-3-yl)methylene]naphthalen-1(2*H*)-one (**11**)

Yellow solid; yield: 73% from methanol; m.p. 136 °C; IR (KBr) cm^−1^: 1696, 1593, 1408, 1286; ^1^H-NMR (CDCl_3_, 600 MHz) δ ppm: 2.91 (t, 2H, H_3_, *J* = 6.0 Hz), 3.03 (t, 2H, H_4_, *J* = 6.0 Hz), 3.85 (s, 3H, OCH_3_), 7.03 (td, 1H, H_5′_, *J* = 7.2 Hz, 7.2 Hz), 7.07 (dd, 1H, H_6_, *J* = 2.4 Hz, *J*_2_ = 8.4 Hz), 7.12–7.17 (m, 4H, H_2″,6″,4″,5_), 7.37 (t, 2H, H_3″_, _5″_, *J* = 7.8 Hz), 7.62 (d, 1H, H_8_, *J* = 3.0 Hz), 7.68 (dd, 1H, H_4′_, *J*_1_ = 1.8 Hz, *J*_2_ = 7.2 Hz), 7.94 (s, 1H, =CH), 8.12 (dd, 1H, H_6′_, *J*_1_ = 1.7 Hz, *J*_2_ = 7.1 Hz). ^13^C-NMR (CDCl_3_, 150.89 MHz) δ ppm: 27.8, 28.0, 55.5, 110.3, 118.0, 120.1, 121.4, 121.6, 124.7, 129.4, 129.5, 130.2, 134.0, 135.9, 137.6, 139.3, 147.4, 153.8, 158.6, 161.4, 187.2 (C=O). MS (*m/z*, %): 264 (27), 118 (16), 115 (12), 77 (100). Anal. calcd. for: C_23_H_19_NO_3_: C 77.29, H 5.36, N 3.92; Found C 77.35, H 5.34, N 4.17.

##### (*E*)-2-{[2-(4-Fluorophenoxy)pyridin-3-yl]methylene}-3,4-dihydronaphthalen-1(2*H*)-one (**12**)

Brown oil; yield: 41% from column chromathography hexane:EtAc (8:2).; IR (KBr) cm^−1^: 1660, 1598, 1420, 1250; ^1^H-NMR (CDCl_3_, 600 MHz) δ ppm: 2.99 (t, 2H, H_3_, *J* = 6.0 Hz), 3.06 (t, 2H, H_4_, *J* = 6.0 Hz), 7.04-7.09 (m, 5H, H_2″,3″,5″,6″,5′_), 7.25 (d, 1H, H_5_, *J* = 8.4 Hz), 7.36 (t, 1H, H_7_, *J* = 7.2 Hz), 7.49 (td, 1H, H_6_, *J*_1_ = 1.8 Hz, *J*_2_ = 7.2 Hz), 7.69 (dd, 1H, H_4′_, *J*_1_ = 1.2 Hz, *J*_2_ = 7.2 Hz), 7.94 (s, 1H, =CH), 8.11 (dd, 1H, H_6′_, *J*_1_ = 1.8 Hz, *J*_2_ = 7.2 Hz), 8.14 (dd, 1H, H_8_, *J*_1_ = 1.2 Hz, *J*_2_ = 7.2 Hz). ^13^C-NMR (CDCl_3_, 150.89 MHz) δ ppm: 27.6, 28.9, 116.1 (*J*_C-3″,5″_ = 27.1 Hz), 118.1, 119.9, 118.1, 120.1, 122.9 (*J*_C-2″,6″_ = 9.0 Hz), 127.1, 128.2, 128.3, 130.0, 133.2, 133.4, 137.7, 139.3, 143.2, 147.3, 149.4 (*J*_C-1″_ = 4.5 Hz), 160.4 (*J*_C-4″_ = 242.9 Hz), 161.5, 187.3 (C=O). MS (*m/z*, %): 264 (52), 118 (23), 115 (12), 77 (47), 95 (100). Anal. calcd. for: C_22_H_16_FNO_2_: C 76.51, H 4.67, N 4.06; Found C 76.53, H 4.69, N 4.21.

##### (*E*)-2-{[2-(4-Fluorophenoxy)pyridin-3-yl]methylene}-3,4-dihydro-5-methoxynaphthalen-1(2*H*)-one (**13**)

Yellow solid; yield: 92% from methanol; m.p. 154–156 °C; IR (KBr) cm^−1^: 1661, 1584, 1414, 1256; ^1^H-NMR (CDCl_3_, 600 MHz) δ ppm: 2.94 (t, 2H, H_3_, *J* = 6.6 Hz), 3.02 (t, 2H, H_4_, *J* = 6.6 Hz), 3.85 (s, 3H, OCH_3_), 7.03–7.09 (m, 6H, H_2″,3″,5″,6″,6,5′_), 7.32 (t, 1H, H_7_, *J* = 7.8 Hz), 7.68 (dd, 1H, H_4′_, *J*_1_ = 1.8 Hz, *J*_2_ = 7.2 Hz), 7.76 (dd, 1H, H_8_, *J*_1_ = 1.8 Hz, *J*_2_ = 7.8 Hz), 7.89 (s, 1H, =CH), 8.10 (dd, 1H, H_6′_, *J*_1_ = 1.8 Hz, *J*_2_ = 7.2 Hz). ^13^C-NMR (CDCl_3_, 150.89 MHz) δ ppm: 21.6, 26.9, 55.7, 114.4, 116.0, 116.2 (J_C-3″,5″_ = 22.6 Hz), 118.1, 120.0, 122.9 (J_C-2″,6″_ = 9.1 Hz), 127.3, 129.7, 132.3, 134.2, 137.7, 139.4, 147.2, 149.5, 156.3, 158.8 (*J*_C-4″_ = 233.9 Hz), 161.5, 187.6 (C=O). MS (*m/z*, %): 264 (64), 148 (7.3), 115 (20), 77 (40), 95 (100). Anal. calcd. for: C_23_H_18_FNO_3_: C 73.59, H 4.83, N 3.73; Found C 73.61, H 4.82, N 3.94.

##### (*E*)-2-{[2-(4-Fluorophenoxy)pyridin-3-yl]methylene}-3,4-dihydro-6-methoxynaphthalen-1(2*H*)-one (**14**)

Beige solid; yield: 83% from methanol; m.p. 152–154 °C; IR (KBr) cm^−1^: 1660, 1503, 1414, 1263; ^1^H-NMR (CDCl_3_, 600 MHz) δ ppm: 2.95 (t, 2H, H_3_, *J* = 6.6 Hz), 3.03 (t, 2H, H_4_, *J* = 6.6 Hz), 3.85 (s, 3H, OCH_3_), 6.69 (d, 1H, H_5_, *J* = 2.4 Hz), 6.87 (dd, 1H, H_7_, *J*_1_ = 2.4 Hz, *J*_2_ = 8.4 Hz), 7.01–7.10 (m, 5H, H_2″,3″,5″,6″,5′_), 7.66 (dd, 1H, H_4′_, *J*_1_ = 1.2 Hz, *J*_2_ = 7.4 Hz), 7.90 (s, 1H, =CH), 8.09 (dd, 1H, H_6′_, *J*_1_ = 1.2 Hz, *J*_2_ = 7.4 Hz), 8.12 (d, 1H, H_8_, *J* = 8.4 Hz). ^13^C-NMR (CDCl_3_, 150.89 MHz) δ ppm: 27.7, 29.3, 55.4, 112.3, 113.4, 116.2 (*J*_C-3″,5″_ = 22.6 Hz), 118.1, 120.1, 122.9 (*J*_C-2″,6″_ = 7.5 Hz), 126.8, 129.4, 130.9, 137.9, 139.3, 145.7, 147.1, 149.5, 160.4 (*J*_C-4″_ = 242.9 Hz), 161.4, 163.7, 186.2 (C=O). MS (*m/z*, %): 264 (64), 148 (30), 115 (13), 77 (42), 95 (100). Anal. calcd. for: C_23_H_18_FNO_3_: C 73.59, H 4.83, N 3.73; Found C 73.57, H 4.86, N 3.91.

##### (*E*)-2-{[2-(4-Fluorophenoxy)pyridin-3-yl]methylene}-3,4-dihydro-7-methoxynaphthalen-1(2*H*)-one (**15**)

Yellow solid; yield: 64% from methanol; m.p. 150–152 °C; IR (KBr) cm^−1^: 1665, 1586, 1419, 1249; ^1^H-NMR (CDCl_3_, 600 MHz) δ ppm: 2.92 (t, 2H, H_3_, *J* = 6.0 Hz), 3.03 (t, 2H, H_4_, *J* = 6.6 Hz), 3.85 (s, 3H, OCH_3_), 7.02–7.10 (m, 6H, H_2″,3″,5″,6″,5,5′_), 7.15 (d, 1H, H_6_, *J* = 8.4 Hz), 7.61 (d, 1H, H_8_, *J* = 3.0 Hz), 7.68 (dd, 1H, H_4′_, *J*_1_ = 1.7 Hz, *J*_2_ = 7.2 Hz), 7.92 (s, 1H, =CH), 8.10 (dd, 1H, H_6′_, *J*_1_ = 1.8 Hz, *J*_2_ = 7.1 Hz). 27.8, 28.1, 55.6, 110.3, 116.2 (*J*_C-3″,5″_ = 15.1 Hz), 118.1, 120.0, 121.7, 123.0 (*J*_C-2″,6″_ = 7.5 Hz), 129.5, 130.1, 134.0, 136.0, 137.7, 139.3, 147.3, 149.4, 158.7, 158.8, 160.4 (*J*_C-4″_ = 153.9 Hz), 187.3 (C=O). MS (*m/z*, %): 264 (35), 148 (27), 115 (31), 77 (48), 95 (100). Anal. calcd. for: C_23_H_18_FNO_3_: C 73.59, H 4.83, N 3.73; Found C 73.61, H 4.83, N 4.02.

##### (*E*)-2-{[2-(4-Bromophenoxy)pyridin-3-yl]methylene}-3,4-dihydronaphthalen-1(2*H*)-one (**16**)

Yellow solid; yield: 76% from methanol; m.p. 138–140 °C; IR (KBr) cm^−1^: 1667, 1611, 1412, 1236; ^1^H-NMR (CDCl_3_, 600 MHz) δ ppm: 2.98 (t, 2H, H_3_, *J* = 6.0 Hz), 3.05 (t, 2H, H_4_, *J* = 6.0 Hz), 7.02 (d, 2H, H_2″,6″_, *J* = 9.0 Hz), 7.06 (dd, 1H, H_5′_, *J*_1_ = 7.2 Hz, *J*_2_ = 7.2 Hz), 7.25 (d, 1H, H_5_, *J* = 6.6 Hz), 7.36 (t, 1H, H_7_, *J* = 7.8 Hz), 7.47-7.50 (m, 3H, H_3″,5″,6_), 7.69 (dd, 1H, H_4′_, *J*_1_ = 1.8 Hz, *J*_2_ = 7.2 Hz), 7.92 (s, 1H, =CH), 8.11 (dd, 1H, H_6′_, *J*_1_ = 1.2 Hz, *J*_2_ = 7.8 Hz), 8.13 (dd, 1H, H_8_, *J*_1_ = 1.8 Hz, *J*_2_ = 7.2 Hz). ^13^C-NMR (CDCl_3_, 150.89 MHz) δ ppm: 27.6, 28.9, 117.0, 118.4, 120.1, 123.3, 127.1, 128.2, 128.3, 129.9, 132.5, 133.2, 133.5, 137.8, 139.4, 143.2, 147.3, 152.8, 161.1, 187.3 (C=O). MS (*m/z*, %): 264 (63), 157 (27), 148 (21), 115 (41), 77 (100). Anal. calcd. for: C_22_H_16_BrNO_2_: C 65.04, H 3.97, N 3.45; Found C 64.98, H 4.01, N 3.62.

##### (*E*)-2-{[2-(4-Bromophenoxy)pyridin-3-yl]methylene}-3,4-dihydro-5-methoxynaphthalen-1(2*H*)-one (**17**)

Yellow solid; yield: 96% from methanol; m.p. 158–160 °C; IR (KBr) cm^−1^: 1663, 1576, 1409, 1247; ^1^H-NMR (CDCl_3_, 600 MHz) δ ppm: 2.95 (t, 2H, H_3_, *J* = 6.0 Hz), 3.01 (t, 2H, H_4_, *J* = 6.0 Hz), 3.85 (s, 3H, OCH_3_), 7.01 (d, 2H, H_2″,6″_, *J* = 6.6 Hz), 7.04-7.06 (m, 2H, H_5′,6_), 7.32 (t, 1H, H_7_, *J* = 7.8 Hz), 7.48 (d, 2H, H_3″,5″_, *J* = 6.6 Hz), 7.70 (dd, 1H, H_4′_, *J*_1_ = 1.2 Hz, *J*_2_ = 7.8 Hz), 7.77 (dd, 1H, H_8_, *J*_1_ = 1.2 Hz, *J*_2_ = 7.8 Hz), 7.87 (s, 1H, =CH), 8.11 (dd, 1H, H_6′_, *J*_1_ = 1.8 Hz, *J*_2_ = 7.8 Hz). ^13^C-NMR (CDCl_3_, 150.89 MHz) δ ppm: 21.6, 28.9, 55.7, 114.5, 117.7, 118.4, 119.9, 120.2, 123.3, 127.3, 129.5, 122.3, 132.5, 134.2, 137.9, 139.5, 147.2, 152.8, 156.3, 161.1, 187.6 (C=O). MS (*m/z*, %): 264 (59), 157 (43), 148 (11), 115 (40), 77 (100). Anal. calcd. for: C_23_H_18_BrNO_3_: C 63.32, H 4.16, N 3.21; Found C 63.34, H 4.19, N 3.37.

##### (*E*)-2-{[2-(4-Bromophenoxy)pyridin-3-yl]methylene}-3,4-dihydro-6-methoxynaphthalen-1(2*H*)-one (**18**)

White solid; yield: 69% from methanol; m.p. 146–148 °C; IR (KBr) cm^−1^: 1665, 1597, 1414, 1260; ^1^H-NMR (CDCl_3_, 600 MHz) δ ppm: 2.94 (t, 2H, H_3_, *J* = 5.4 Hz), 3.02 (t, 2H, H_4_, *J* = 5.4 Hz), 3.86 (s, 3H, OCH_3_), 6.69 (d, 1H, H_5_, *J* = 2.4 Hz), 6.87 (dd, 1H, H_5′_, *J*_1_ = 9.0 Hz, *J*_2_ = 9.0 Hz), 7.02 (d, 2H, H_2″,6″_, *J* = 9.0 Hz), 7.04 (dd, 1H, H_7_, *J*_1_ = 2.4 Hz, *J*_2_ = 7.2 Hz), 7.48 (d, 2H, H_3″,5″_, *J* = 9.0 Hz), 7.68 (dd, 1H, H_4′_, *J*_1_ = 1.2 Hz, *J*_2_ = 7.6Hz), 7.88 (s, 1H, =CH), 8.09 (dd, 1H, H_6′_, *J*_1_ = 1.2 Hz, *J*_2_ = 7.8 Hz), 8.10 (d, 1H, H_8_, *J* = 8.0 Hz). ^13^C-NMR (CDCl_3_, 150.89 MHz) δ ppm: 27.7, 29.3, 55.4, 112.3, 113.4, 117.7, 118.4, 120.3, 123.3, 126.8, 129.2, 130.9, 132.5, 138.0, 139.4, 145.7, 147.1, 152.8, 161.1, 163.7, 186.2 (C=O). MS (*m/z*, %): 264 (89), 157 (16), 148 (21), 115 (12), 77 (33). Anal. calcd. for: C_23_H_18_BrNO_3_: C 63.32, H 4.16, N 3.21; Found C 63.29, H 4.17, N 3.40.

##### (*E*)-2-{[2-(4-Bromophenoxy)pyridin-3-yl]methylene}-3,4-dihydro-7-methoxynaphthalen-1(2*H*)-one (**19**)

White solid; yield: 62% from methanol; m.p. 144–146 °C; IR (KBr) cm^−1^: 1660, 1590, 1417, 1241; ^1^H-NMR (CDCl_3_, 600 MHz) δ ppm: 2.93 (t, 2H, H_3_, *J* = 6.6 Hz), 3.02 (t, 2H, H_4_, *J* = 6.6 Hz), 3.85 (s, 3H, OCH_3_), 7.01 (d, 2H, H_2″,6″_, *J* = 9.0 Hz), 7.02 (dd, 1H, H_5′_, *J*_1_ = 7.2 Hz, *J*_2_ = 7.2 Hz), 7.03 (dd, 1H, H_6_, *J*_1_ = 2.4 Hz, *J*_2_ = 8.4 Hz), 7.15 (d, 1H, H_5_, *J* = 8.4 Hz), 7.49 (d, 2H, H_3″,5″_, *J* = 9.0 Hz), 7.62 (d, 1H, H_8_, *J* = 2.4 Hz), 7.68 (dd, 1H, H_4′_, *J*_1_ = 1.8 Hz, *J*_2_ = 7.2 Hz), 7.88 (s, 1H, =CH), 8.11 (dd, 1H, H_6′_, *J*_1_ = 1.8 Hz, *J*_2_ = 7.2 Hz). ^13^C-NMR (CDCl_3_, 150.89 MHz) δ ppm: 27.8, 28.1, 121.8, 123.0, 129.9, 130.0, 132.5, 134.0, 135.9, 137.7, 139.4, 147.3, 152.8, 158.7, 161.1, 187.2 (C=O). MS (*m/z*, %): 264 (67), 157 (22), 148 (31), 115 (13), 77 (100). Anal. calcd. for: C_23_H_18_BrNO_3_: C 63.32, H 4.16, N 3.21; Found C 63.31, H 4.17, N 3.38.

##### (*E*)-2-{[2-(4-Chlorophenoxy)pyridin-3-yl]methylene}-3,4-dihydronaphthalen-1(2*H*)-one (**20**)

Yellow solid; yield: 74% from methanol; m.p. 130 °C; IR (KBr) cm^−1^: 1669, 1483, 1411, 1289; ^1^H-NMR (CDCl_3_, 600 MHz) δ ppm: 2.98 (t, 2H, H_3_, *J* = 5.4 Hz), 3.04 (t, 2H, H_4_, *J* = 5.4 Hz), 7.07 (dd, 1H, H_5_, *J*_1_ = 1.8 Hz, *J*_2_ = 7.2 Hz), 7.08 (dd, 2H, H_2″,6″_, *J*_1_ = 3.0 Hz, *J*_2_ = 8.4 Hz), 7.24 (dd, 1H, H_5′_, *J*_1_ = 6.6 Hz, *J*_2_ = 6.6 Hz), 7.33 (dd, 2H, H_3″,5″_, *J*_1_ = 3.0 Hz, *J*_2_ = 8.4 Hz), 7.36 (t, 1H, H_7_, *J* = 7.2 Hz), 7.48 (t, 1H, H_6_, *J* = 7.2 Hz), 7.69 (dd, 1H, H_4′_, *J*_1_ = 1.2 Hz, *J*_2_ = 7.5 Hz), 7.93 (s, 1H, =CH), 8.11 (d, 1H, H_8_, *J* = 7.2 Hz), 8.13 (dd, 1H, H_6′_, *J*_1_ = 1.2 Hz, *J*_2_ = 7.6 Hz). ^13^C-NMR (CDCl_3_, 150.89 MHz) δ ppm: 27.6, 28.8, 118.3, 120.1, 122.8, 127.1, 128.2, 128.3, 129.5, 129.8, 130.0, 133.2, 133.4, 137.8, 139.4, 148.2, 147.2, 152.2, 161.1, 187.3 (C=O). MS (*m/z*, %): 234 (32), 118 (23), 115 (21), 111 (35), 77 (26). Anal. calcd. for: C_22_H_16_ClNO_2_: C 73.03, H 4.46, N 3.87; Found C 72.99, H 4.47, N 4.02.

##### (*E*)-2-{[2-(4-Chlorophenoxy)pyridin-3-yl]methylene}-3,4-dihydro-5-methoxynaphthalen-1(2*H*)-one (**21**)

Yellow solid; yield: 73% from methanol; m.p. 164–166 °C; IR (KBr) cm^−1^: 1664, 1571, 1408, 1276; ^1^H-NMR (CDCl_3_, 600 MHz) δ ppm: 2.95 (t, 2H, H_3_, *J* = 6.6 Hz), 3.01 (t, 2H, H_4_, *J* = 6.6 Hz), 3.85 (s, 3H, OCH_3_), 7.03 (dd, 1H, H_6_, *J*_1_ = 1.8 Hz, *J*_2_ = 7.8 Hz), 7.05 (dd, 1H, H_5′_, *J*_1_ = 7.2 Hz, *J*_2_ = 7.2 Hz), 7.08 (d, 2H, H_2″,6″_, *J* = 9.0 Hz), 7.30 (t, 1H, H_7_, *J* = 7.8 Hz), 7.33 (d, 2H, H_3″,5″_, *J* = 9.0 Hz), 7.69 (dd, 1H, H_4′_, *J*_1_ = 1.8 Hz, *J*_2_ = 7.2 Hz), 7.77 (dd, 1H, H_8_, *J*_1_ = 1.8 Hz, *J*_2_ = 7.2 Hz), 7.88 (s, 1H, =CH), 8.11 (dd, 1H, H_6′_, *J*_1_ = 1.8 Hz, *J*_2_ = 7.8 Hz). ^13^C-NMR (CDCl_3_, 150.89 MHz) δ ppm: 21.5, 26.9, 55.7, 114.4, 118.3, 119.9, 120.1, 122.8, 127.2, 129.5, 130.0, 132.3, 134.1, 137.8, 139.4, 147.2, 152.2, 156.3, 161.1, 187.5 (C=O). MS (*m/z*, %): 264 (48), 148 (25), 115 (17), 111 (32), 77 (35). Anal. calcd. for: C_23_H_18_ClNO_3_: C 70.50, H 4.63, N 3.57; Found C 70.51, H 4.65, N 3.71.

##### (*E*)-2-{[2-(4-Chlorophenoxy)pyridin-3-yl]methylene}-3,4-dihydro-6-methoxynaphthalen-1(2*H*)-one (**22**)

Yellow solid; yield: 76% from methanol; m.p. 114 °C; IR (KBr) cm^−1^: 1660, 1484, 1414, 1270; ^1^H-NMR (CDCl_3_, 600 MHz) δ ppm: 2.94 (t, 2H, H_3_, *J* = 6.0 Hz), 3.02 (t, 2H, H_4_, *J* = 6.0 Hz), 3.85 (s, 3H, OCH_3_), 6.69 (d, 1H, H_5_, *J* = 2.4 Hz), 6.87 (dd, 1H, H_5′_, *J*_1_ = 6.0 Hz, *J*_2_ = 6.0 Hz), 7.07 (dd, 1H, H_7_, *J*_1_ = 2.4 Hz, *J*_2_ = 7.2 Hz), 7.04 (d, 2H, H_2″,6″_, *J* = 9.0 Hz), 7.33 (d, 2H, H_3″,5″_, *J* = 9.0 Hz), 7.68 (dd, 1H, H_4′_, *J*_1_ = 1.2 Hz, *J*_2_ = 7.7 Hz), 7.88 (s, 1H, =CH), 8.09 (dd, 1H, H_6′_, *J*_1_ = 1.2 Hz, *J*_2_ = 7.5 Hz), 8.13 (d, 1H, H_8_, *J* = 7.2 Hz). ^13^C-NMR (CDCl_3_, 150.89 MHz) δ ppm: 27.6, 29.2, 55.4, 112.3, 113.4, 118.3, 120.2, 122.8, 126.8, 129.2, 129.5, 129.5, 130.8, 137.9, 139.4, 145.7, 147.1, 152.2, 161.1, 163.7, 186.1 (C=O). MS (*m/z*, %): 264 (29), 148 (15), 115 (29), 111 (43), 77 (54). Anal. calcd. for: C_23_H_18_ClNO_3_: C 70.50, H 4.63, N 3.57; Found C 70.56, H 4.62, N 3.69.

##### (*E*)-2-{[2-(4-Chlorophenoxy)pyridin-3-yl]methylene}-3,4-dihydro-7-methoxynaphthalen-1(2*H*)-one (**23**)

Yellow solid; yield: 67% from methanol; m.p. 160 °C; IR (KBr) cm^−1^: 1695, 1590, 1414, 1280; ^1^H-NMR (CDCl_3_, 600 MHz) δ ppm: 2.92 (t, 2H, H_3_, *J* = 6.0 Hz), 3.02 (t, 2H, H_4_, *J* = 6.0 Hz), 3.85 (s, 3H, OCH_3_), 7.03-7.04 (m, 2H, H_6,5′_), 7.07 (d, 2H, H_2″,6″_, *J* = 9.0 Hz), 7.16 (d, 1H, H_5_, *J* = 7.2 Hz), 7.34 (d, 2H, H_3″,5″_, *J* = 9.0 Hz), 7.61 (d, 1H, H_8_, *J* = 3 Hz), 7.69 (dd, 1H, H_4′_, *J*_1_ = 1.8 Hz, *J*_2_ = 7.2 Hz), 7.91 (s, 1H, =CH), 8.11 (dd, 1H, H_6′_, *J*_1_ = 1.8 Hz, *J*_2_ = 7.8 Hz). ^13^C-NMR (CDCl_3_, 150.89 MHz) δ ppm: 27.8, 28.0, 55.5, 110.3, 118.3, 120.1, 121.7, 122.8, 129.5, 129.5, 129.9, 130.0, 134.0, 135.9, 137.8, 139.4, 147.2, 152.2, 158.7, 161.1, 187.2 (C=O). MS (*m/z*, %): 234 (25), 121 (16), 115 (11), 111 (26), 77 (29). Anal. calcd. for: C_23_H_18_ClNO_3_: C 70.50, H 4.63, N 3.57; Found C 70.54, H 4.63, N 3.75.

### 3.2. Biological Assays

#### 3.2.1. Culture and Maintenance of Parasite

Promastigotes of *Leishmania* (V.) *braziliensis* strain MHOM/CO/87/UA301 were isolated from footpad lesions in Balb/C mice previously infected. In the process of maintenance and differentiation of the parasites, the LTI medium (tryptose 15 g/L, yeast extract 5 g/L, liver extract 2 g/L, hemin-NaOH 0.02 g/L, glucose 4 g/L, NaCl 9 g/L, KCl 0.4 g/L Na_2_HPO_4_, 7.5 g/L at pH 7.4) supplemented with 10% fetal calf serum and maintained at 29 °C was used.

*T. cruzi* epimastigotes MHOM/VE/92/YBM venezuelan strain were used to test the anti *T. cruzi* in vitro studies. The strain was maintained in a modified LIT medium with 10% fetal bovine serum (FBS). Mouse bone marrow was used to obtain BMDM macrophages. Mouse fibroblast-conditioned medium L-929 was used for the differentiation and maintenance of BMDM macrophages [57].

Vero cells were derived from the kidney of an African green monkey, cells were maintained in Dulbecco’s Modified Eagle’s Medium (DMEM), supplemented with 10% heat-inactivated FBS and penicillin-streptomycin solution 1% at 37 °C, 5% CO_2_ atmosphere and > 95% humidity [58].

#### 3.2.2. Leishmanicidal Activity

We evaluated through a colorimetric method the effect of compounds **8**–**23** on the promastigotes of *L. brasiliensis* [59]. Briefly, 96-well plates were used, where 2 × 10^6^ parasites/mL were seeded. A concentration of 50 μM was used for each compound and an incubation time of 96 h at 29 °C. 3-(4,5-dimethylthiazol-2-yl)-2,5-diphenyl tetrazolium bromide (MTT) 1 μg/mL was added and incubated in darkness for 4 h, acidic isopropanol (4N) was added and the plate was read at 570 nm in a spectrophotometer Synergy HT (Biotek). Miltefosine was used as a reference drug. The EC_50_ calculation of the selected compound was performed using growth curves. Direct counting was used to monitor the proliferation of parasites, three independent experiments were performed for each concentration of the compounds evaluated. The in vitro evaluations of compounds toxicity by MTT in VERO and BMDM cells follow a methodology described above with some modifications [60]. The selectivity index was calculated with SI = CC_50_/EC_50_, where CC_50_ was the maximum concentration range of compounds **10**, **21**–**23** used to evaluate cytotoxicity in VERO and BMDM cells.

#### 3.2.3. Trypanocidal Activity

The trypanocidal activity of compounds **8**–**23** was evaluated on the viability of *T. cruzi* epimastigotes through a colorimetric method adapted with minor modifications [57]. 2 × 10^6^ parasites/mL were seeded in a 96-well plate, adding 50 μM of each derivative dissolved in DMSO (final concentration remained below 1%). The plate was incubated for 96 h at 29 °C. Then, 1 μg/mL of 3-(4,5-dimethylthiazol-2-yl)-2,5-diphenyl tetrazolium bromide (MTT) was added and incubated in darkness for 4 h. After this time, acidic isopropanol (4N) was added and the plate was read at 570 nm in a spectrophotometer Synergy HT (Biotek). Benznidazole was used as a reference drug. The in vitro evaluations of compounds toxicity by MTT in VERO and BMDM cells follow a methodology described above with some modifications [60]. Cells were counted in suspension and seeded at 20 × 10^3^ cells/well. The test was carried out in triplicate on 96-well microplates in different concentrations: 50 μM, 100 μM, 200 μM and 300 μM, untreated cells and reference drug (benznidazole) controls.

#### 3.2.4. Cell Lines

The cell lines used were acquired from the American Type Culture Collection (ATCC), CCRF-CEM (T lymphoblastic leukemia), K562 (acute myeloid leukemia), U2OS (osteosarcoma), HCT116 (colon carcinoma), A549 (lung adenocarcinoma) and HCT116p53−/− (HCT116 cell line mutated in p53) Horizon Discovery Ltd., (Waterbeach, Cambridge, United Kingdom).

In our laboratory, IMTM, we generated the daunorubicin-resistant subline of CCRF-CEM cells (CEM-DNR bulk) and paclitaxel-resistant subline K562-TAX, as described previously [61]. Overexpression of MRP-1 and P-glycoprotein protein was observed in the CEM-DNR cell and K562-TAX cells overexpress P-glycoprotein only. Both ABC transporters are involved in drug resistance [61,62]. As a control, human fibroblasts MRC-5 and BJ cell lines were used. The culture media used were: DMEM, RPMI 1640, and MEM (according to a cell line); all were supplemented with 5 g L^−1^ glucose, 10% fetal calf serum, 2 mM glutamine, 100 U mL^−1^ penicillin, 100 µg mL^−1^ streptomycin, and NaHCO3[61,62]. Adherent cells were passaged using PBS containing 0.25% trypsin plus 0.01% EDTA (ethylenediamine tetraacetic acid) every 2–3 days.

#### 3.2.5. Antiproliferative Activity Assay

The antiproliferative activity of compounds was determined using a standard 3-(4,5-dimethylthiazol-2yl)-5-(3-carboxymethoxyphenyl)-2-(4-sulfophenyl)-2*H*-tetrazolium (MTS) reduction assay on a robotic high throughput screening platform (HighResBio, Boston, MA, USA) following treatment of cells for 3–7 days. The IC_50_ values were calculated from the appropriate dose–response curves with Dotmatics software (San Diego, CA, USA) [61,62,63].

#### 3.2.6. Apoptosis Assay

K562, A549 cell lines were treated as described by the manufacturer (Santa Cruz Biotechnology), washed, and resuspended in annexin V binding buffer (0.01 M HEPES, 0.14 M NaCl, and 2.5 mM CaCl_2_) with the subsequent incubation of annexinV-FITC and then PI. An Epics XL cytometer (Beckman Coulter, Indianapolis, IN, USA) was used for the analysis. Untreated cells were the negative controls, and doxorubicin (Dox) (1 µM), and quercetin (QC) (50 µM) were the positive controls [64,65,66]. Early apoptosis refers to annexin V expression, late apoptosis, the coexpression of annexin V and PI, and necrosis the sole expression of PI. Normal fresh lymphocytes were not affected by the compounds, up to 5 µM. The experiments were performed as described before [64,65,66]. To avoid the unwanted effects of DMSO, the concentrations of the compounds were never higher than 100 µM. Since the IC50 values of the two most active derivatives 18 and 22 in both cell lines were: 3.91 ± 0.14 µΜ to 4.33 ± 0.19 µΜ for K562 cells and 9.64 ± 1.71 µΜ to 9.74 ± 1.28 µΜ for A549 cells, the experiments were carried out at 5, 10, and 25 µΜ, Figure 2 and Figure 3.

## 4. Conclusions

In conclusion, the 16 compounds were synthesized by two-step synthesis, involving nucleophilic aromatic substitution and the classical Claisen–Schmidt condensation base-catalyzed in methanol at room temperature. All the title compounds were obtained in good yields and characterized by spectroscopic technique. Two compounds showed in vitro activities against promastigotes of *Leishmania* (V.) *braziliensis* strain MHOM/CO/87/UA301 and epimastigotes of *T. cruzi* MHOM/VE/92/YBM Venezuelan strain. The most active were compounds **22** and **23** exerting micromolar parasiticide effects and also good selectivity with low toxicity to BMDM and VERO cells.

Ten compounds showed in vitro antiproliferative activities against a broad panel of cancer cell lines with an IC_50_ < 10 µM. The most active were compounds **18** and **22** exerting micromolar antiproliferative effects and also good selectivity to proliferating cancer cell lines with low toxicity to non-malignant MRC-5 or BJ fibroblasts. Unfortunately, except for compound **19**, low cytotoxicity was observed against multidrug-resistant cancer cell lines (CEM-DNR, K562-TAX), suggesting these results that for resistance is responsible other mechanisms than P-glycoprotein. Flow cytometry analysis confirmed the induction of apoptosis by parts of compounds **18** and **22** in treated cells after 24 h. Therefore, we can infer that the antiparasitic and antiproliferative effects of this class of compounds are related to the type of halogen atoms and the position of the methoxy group in the tetralone nucleus.

## Figures and Tables

**Table 1:** Effect of compounds **8**–**23** on the viability of promastigotes of *Leishmania* (V.) *braziliensis*, epimastigotes of *Trypanosoma cruzi*, BMDM macrophages, and VERO cells.

			EC_50_ (µM) 72 h							
No	R_1_	R_2_	*L. braziliensis*	*T. cruzi*	VERO	BMDM	SI_V/Lb_	SI_B/Lb_	SI_V/Tc_	SI_B/Tc_
**8**	H	H	57.65 ± 1.62	>60	>100	>1000	ND	ND	ND	ND
**9**	H	5-OCH_3_	>60	>60	<100	>1000	ND	ND	ND	ND
**10**	H	6-OCH_3_	22.57 ± 1.15	>60	89 ± 15	<1000	4	ND	ND	ND
**11**	H	7-OCH_3_	>60	>60	<100	<1000	ND	ND	ND	ND
**12**	F	H	>100	>100	-	-	-	-	-	-
**13**	F	5-OCH_3_	>100	>100	-	-	-	-	-	-
**14**	F	6-OCH_3_	>100	>100	-	-	-	-	-	-
**15**	F	7-OCH_3_	>100	>100	-	-	-	-	-	-
**16**	Br	H	>100	>100	-	-	-	-	-	-
**17**	Br	5-OCH_3_	>100	>100	-	-	-	-	-	-
**18**	Br	6-OCH_3_	>100	>100	-	-	-	-	-	-
**19**	Br	7-OCH_3_	44.57 ± 1.15	>100	-	-	-	-	-	-
**20**	Cl	H	>60	57.38 ± 3.6	>1000	>1000	ND	ND	ND	ND
**21**	Cl	5-OCH_3_	9.24 ± 1.00	35.5 ± 10	103 ± 15	>1000	11	ND	3	ND
**22**	Cl	6-OCH_3_	12.37 ± 1.15	5.03 ± 0.49	>100	468	ND	38	ND	93
**23**	Cl	7-OCH_3_	5.69 ± 2.65	4.91 ± 0.98	100 ± 16	>1000	18	ND	20	ND
**Mil**	-	-	25 ± 1.4	-	ND	125 ± 4.7	ND	5	-	-
**Bnz**	-	-	-	30.00 ± 0.7	120 ± 2.3	ND	-	-	4	ND

EC_50_: Effective concentration; Mil: miltefosine; Bnz: benznidazole; *L. braziliensis*: Strain MHOM/CO/87/UA301 promastigotes; *T. cruzi*: Strain MHOM/VE/92/YBM epimastigotes; VERO cells are derived from the kidney of an African green monkey; BMDM macrophages were obtained from mouse bone marrow; SI_v/Lb_: selectivity index on *L. braziliensis*/VERO; SI_B/Lb_: selectivity index on *L. braziliensis*/BMDM; SI_v/Tc_: selectivity index on *T. cruzi*/VERO; SI_B/Tc_: selectivity index on *L. cruzi*/BMDM; ND = not determined.

**Table 2:** Effect of compounds **8**–**23** after 72 h of incubation with cancerous and noncancerous human cell lines and drug resistance profile.

No	R_1_	R_2_	CCRF-CEM	CEM-DNR	K562	K562-TAX	A549	HCT-116	HCT116p53	U2OS
**8**	H	H	4.15 ± 0.74	38.16 ± 6.26	>50	20.0 ± 2.16	>50	>50	>50	>50
**9**	H	5-OCH_3_	6.15 ± 0.84	26.3 ± 7.95	19.55 ± 3.67	24.1 ± 6.02	29.97 ± 5.83	24.81 ± 3.77	20.21 ± 3.1	19.63 ± 3.65
**10**	H	6-OCH_3_	13.25 ± 1.82	33.74 ± 6.78	44.59 ± 3.67	30.07 ± 7.28	>50	>50	>50	>50
**11**	H	7-OCH_3_	8.79 ± 0.76	45.08 ± 7.9	>50	>50	>50	>50	>50	>50
**12**	F	H	6.7 ± 0.98	35.57 ± 4.42	22.45 ± 2.3	32.92 ± 3.19	28.12 ± 4.86	26.37 ± 1.58	26.89 ± 3.49	23.16 ± 1.90
**13**	F	5-OCH_3_	6.07 ± 0.59	37.26 ± 3.13	23.36 ± 2.31	39.3 ± 5.4	28.42 ± 2.14	27.19 ± 2.44	23.48 ± 1.5	20.96 ± 2.21
**14**	F	6-OCH_3_	20.1 ± 2.43	41.28 ± 5.67	5.74 ± 3.2	39.25 ± 9.37	43.65 ± 4.12	>50	>50	>50
**15**	F	7-OCH_3_	9.13 ± 1.38	23.1 ± 4.38	27.66 ± 2.49	19.67 ± 3.78	29.08 ± 5.0	30.51 ± 2.34	23.87 ± 2.5	26.32 ± 4.61
**16**	Br	H	8.7 ± 2.19	34.26 ± 3.61	23.87 ± 2.28	25.61 ± 5.37	39.7 ± 1.89	29.92 ± 4.59	31.47 ± 62.72	38.03 ± 4.47
**17**	Br	5-OCH_3_	12.93 ± 2.07	31.66 ± 5.86	32.04 ± 3.72	31.38 ± 6.59	>50	>50	>50	>50
**18**	Br	6-OCH_3_	5.97 ± 0.67	25.73 ± 5.71	4.33 ± 0.19	17.62 ± 7.09	9.64 ± 1.71	>50	>50	7.27 ± 0.68
**19**	Br	7-OCH_3_	12.89 ± 2.43	19.98 ± 3.99	42.95 ± 5.1	4.17 ± 2.93	>50	>50	>50	>50
**20**	Cl	H	6.07 ± 0.63	28.22 ± 4.83	23.63 ± 2.55	32.49 ± 2.93	37.04 ± 3.98	26.21 ± 2.4	25.57 ± 3.77	27.86 ± 4.13
**21**	Cl	5-OCH_3_	10.37 ± 2.46	35.76 ± 4.25	32.71 ± 3.67	23.56 ± 8.79	46.92 ± 4.02	38.11 ± 5.44	31.13 ± 4.45	46.76 ± 4.26
**22**	Cl	6-OCH_3_	5.23 ± 0.30	22.53 ± 4.77	3.91 ± 0.14	21.44 ± 4.08	9.74 ± 1.28	12.95 ± 3.61	33.9 ± 5.63	8.1 ± 1.71
**23**	Cl	7-OCH_3_	15.65 ± 1.54	23.95 ± 4.98	>50	16.05 ± 4.44	>50	>50	>50	>50

Compound **8**–**23** against **MRC5** and **BJ** noncancerous human cell lines IC_50_ > 50 µM. IC_50_ is the lowest concentration that kills 50% of cells. Compounds with IC_50_ > 50 µM are considered inactive.

## Data Availability

Not applicable.

## Supplementary Materials

The following supporting information can be downloaded at: https://www.mdpi.com/article/10.3390/molecules27175626/s1, ^1^H/^13^C NMR for compounds **3**–**6** and ^1^H/^13^C NMR, and mass spectra for compounds **8**, **10**, **13**, **14**, **17**, **18**, **21**, and **22**; Tables S1 and S2.

## Sample Availability

Samples of the compound are available from the authors.
