# Supplementary material for: Synthesis, Leishmanicidal, Trypanocidal, Antiproliferative Assay and Apoptotic Induction of (2-Phenoxypyridin-3-yl)naphthalene-1(2H)-one Derivatives
Source: Molecules. 2022 Aug 31;27(17):5626. doi: 10.3390/molecules27175626 (PMC9457600; doi:10.3390/molecules27175626)
Supplement: Supplementary file 1 [file molecules-27-05626-s001.zip › molecules-1860744-supplementary.pdf]

**Supporting Information for**

# Synthesis, leishmanicidal, trypanocidal, antiproliferative assay and apoptotic induction of (2-phenoxypyridin-3-yl)naphthalene-1(2*H*)-one derivatives

Zuleima Blanco[1], Esteban Fernandez-Moreira[2], Michael R. Mijares[3,4], Carmen Celis[3], Gricelis Martínez[3], Juan B. De Santics[4,5], Soňa Gurská[5], Petr Džubák[5], Marián Hajdůch[5], Ali Mijoba[6], Yael García[6], Xenón Serrano[6], Nahum Herrera[7], Jhonny Correa-Abril[7], Yonathan Parra[7], Jorge Ángel,[8] Hegira Ramírez[9]* and Jaime E. Charris[1,*]

[1] Organic Synthesis Laboratory, Faculty of Pharmacy, Central University of Venezuela, 47206, Los Chaguaramos 1041-A, Caracas, Venezuela

[2] Escuela de Medicina, Universidad Espíritu Santo, Samborondón, Ecuador

[3] Biotechnology Unit, Faculty of Pharmacy, Central University of Venezuela, 47206, Los Chaguaramos 1041-A, Caracas, Venezuela

[4] Institute of Immunology, Faculty of Medicine, Central University of Venezuela, 50109, Los Chaguaramos 1050-A, Caracas, Venezuela

[5] Institute of Molecular and Translational Medicine, Faculty of Medicine, Palacky University, Hněvotínská 1333/5, Olomouc 779 00, Czech Republic

[6] Laboratory of Biology and Chemotherapy of Tropical Parasitosis of the Foundation Institute for Advanced Studies (IDEA) Health Area, 1080, Caracas, Venezuela

[7] Grupo de Investigación en Moléculas y Materiales Funcionales (MoléMater), Dirección de Investigación, Universidad Central del Ecuador. Jerónimo Leiton s/n y Gilberto Gatto Sobral, 170521, Quito. Ecuador

[8] Laboratorio de Síntesis Orgánica y Diseño de Fármacos, Dpto. de Química, Facultad Experimental de Ciencias, Universidad del Zulia, Maracaibo, Venezuela

[9] Universidad ECOTEC, Km. 13.5 Vía Samborondón, Samborondón, Guayas, Ecuador

## Table of Contents of Figure caption


| | | Page |
|---|---|---|
| **S1** | The $^{1}H$ NMR spectrum of **3** | 3 |
| **S2** | The $^{13}C$ NMR spectrum of **3** | 4 |
| **S3** | The $^{1}H$ NMR spectrum of **4** | 5 |
| **S4** | The $^{13}C$ NMR spectrum of **4** | 6 |
| **S5** | The $^{1}H$ NMR spectrum of **5** | 7 |
| **S6** | The $^{13}C$ NMR spectrum of **5** | 8 |
| **S7** | The $^{1}H$ NMR spectrum of **6** | 9 |
| **S8** | The $^{13}C$ NMR spectrum of **6** | 10 |
| **S9** | The $^{1}H$ NMR spectrum of **8** | 11 |
| **S10** | The $^{13}C$ NMR spectrum of **8** | 12 |
| **S11** | The Mass fragmentation of **8** | 13 |
| **S12** | The $^{1}H$ NMR spectrum of **10** | 14 |
| **S13** | The $^{13}C$ NMR spectrum of **10** | 15 |
| **S14** | The Mass fragmentation of **10** | 16 |
| **S15** | The $^{1}H$ NMR spectrum of **13** | 17 |
| **S16** | The $^{13}C$ NMR spectrum of **13** | 18 |
| **S17** | The Mass fragmentation of **13** | 19 |
| **S18** | The $^{1}H$ NMR spectrum of **14** | 20 |
| **S19** | The $^{13}C$ NMR spectrum of **14** | 21 |
| **S20** | The Mass fragmentation of **14** | 22 |
| **S21** | The $^{1}H$ NMR spectrum of **17** | 23 |
| **S22** | The $^{13}C$ NMR spectrum of **17** | 24 |
| **S23** | The Mass fragmentation of **17** | 25 |
| **S24** | The $^{1}H$ NMR spectrum of **18** | 26 |
| **S25** | The $^{13}C$ NMR spectrum of **18** | 27 |
| **S26** | The Mass fragmentation of **18** | 28 |
| **S27** | The $^{1}H$ NMR spectrum of **21** | 29 |
| **S28** | The $^{13}C$ NMR spectrum of **21** | 30 |
| **S29** | The Mass fragmentation of **21** | 31 |
| **S30** | The $^{1}H$ NMR spectrum of **22** | 32 |
| **S31** | The $^{13}C$ NMR spectrum of **22** | 33 |
| **S32** | The Mass fragmentation of **22** | 34 |

**Figure S1**

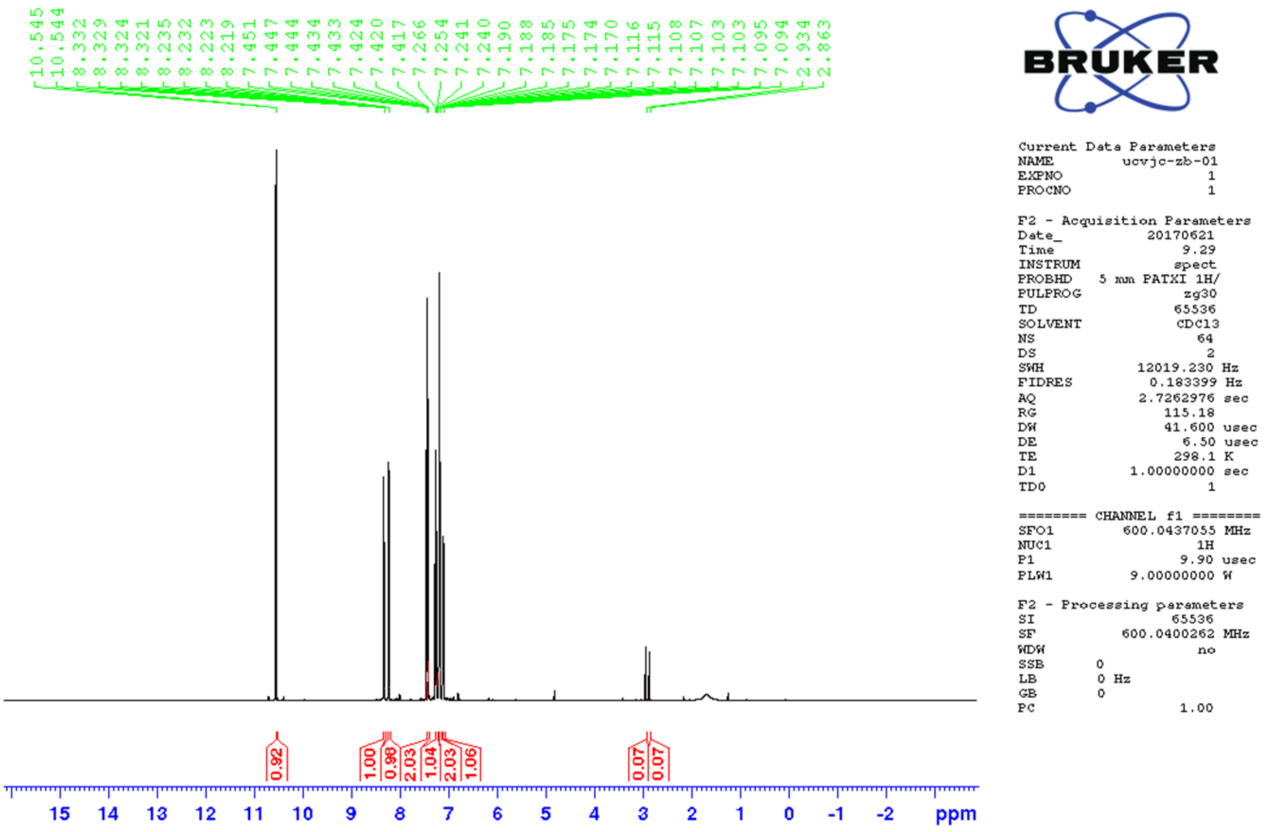

10.545
10.544
8.332
8.329
8.324
8.321
8.235
8.232
8.223
8.219
7.451
7.447
7.444
7.434
7.433
7.424
7.420
7.417
7.266
7.254
7.241
7.240
7.190
7.188
7.185
7.175
7.174
7.170
7.116
7.115
7.108
7.107
7.103
7.103
7.095
7.094
2.934
2.863
0.92
1.00
0.98
2.03
1.04
2.03
1.06
0.07
0.07
15 14 13 12 11 10 9 8 7 6 5 4 3 2 1 0 -1 -2 ppm
BRUKER
Current Data Parameters
NAME ucvjc-zb-01
EXPNO 1
PROCNO 1
F2 - Acquisition Parameters
Date_ 20170621
Time 9.29
INSTRUM spect
PROBHD 5 mm PATXI 1H/
PULPROG zg30
TD 65536
SOLVENT CDCl3
NS 64
DS 2
SWH 12019.230 Hz
FIDRES 0.183399 Hz
AQ 2.7262976 sec
RG 115.18
DW 41.600 usec
DE 6.50 usec
TE 298.1 K
D1 1.00000000 sec
TD0 1
======== CHANNEL f1 ========
SFO1 600.0437055 MHz
NUC1 1H
P1 9.90 usec
PLW1 9.00000000 W
F2 - Processing parameters
SI 65536
SF 600.0400262 MHz
WDW no
SSB 0
LB 0 Hz
GB 0
PC 1.00

**Figure S2**

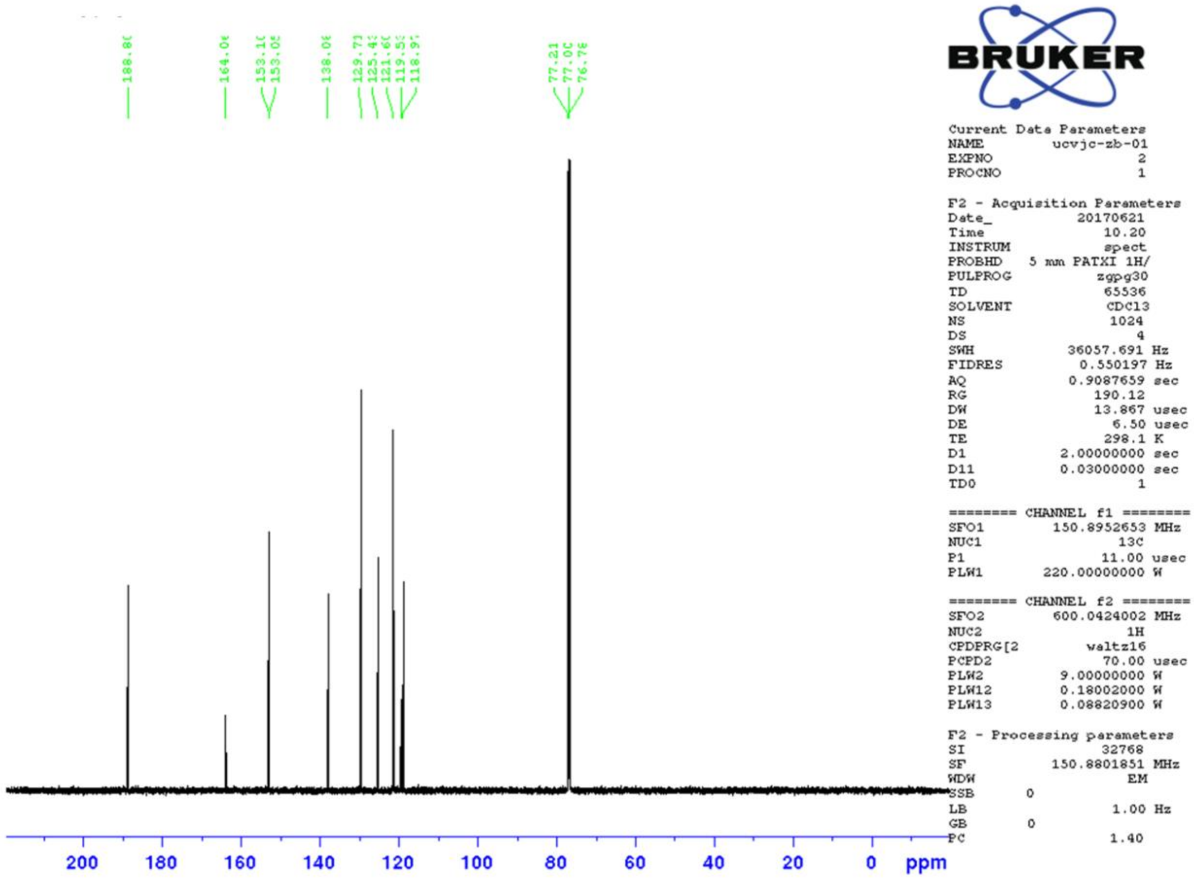
188.80
164.06
153.10
153.05
138.08
129.73
125.43
121.60
119.53
118.93
77.21
77.00
76.78
BRUKER
Current Data Parameters
NAME ucvjc-zb-01
EXPNO 2
PROCNO 1
F2 - Acquisition Parameters
Date_ 20170621
Time 10.20
INSTRUM spect
PROBHD 5 mm PATXI 1H/
PULPROG zgpg30
TD 65536
SOLVENT CDCl3
NS 1024
DS 4
SWH 36057.691 Hz
FIDRES 0.550197 Hz
AQ 0.9087659 sec
RG 190.12
DW 13.867 usec
DE 6.50 usec
TE 298.1 K
D1 2.00000000 sec
D11 0.03000000 sec
TD0 1
======== CHANNEL f1 ========
SFO1 150.8952653 MHz
NUC1 13C
P1 11.00 usec
PLW1 220.00000000 W
======== CHANNEL f2 ========
SFO2 600.0424002 MHz
NUC2 1H
CPDPRG[2 waltz16
PCPD2 70.00 usec
PLW2 9.00000000 W
PLW12 0.18002000 W
PLW13 0.08820900 W
F2 - Processing parameters
SI 32768
SF 150.8801851 MHz
WDW EM
SSB 0
LB 1.00 Hz
GB 0
PC 1.40
200 180 160 140 120 100 80 60 40 20 0 ppm

**Figure S3**

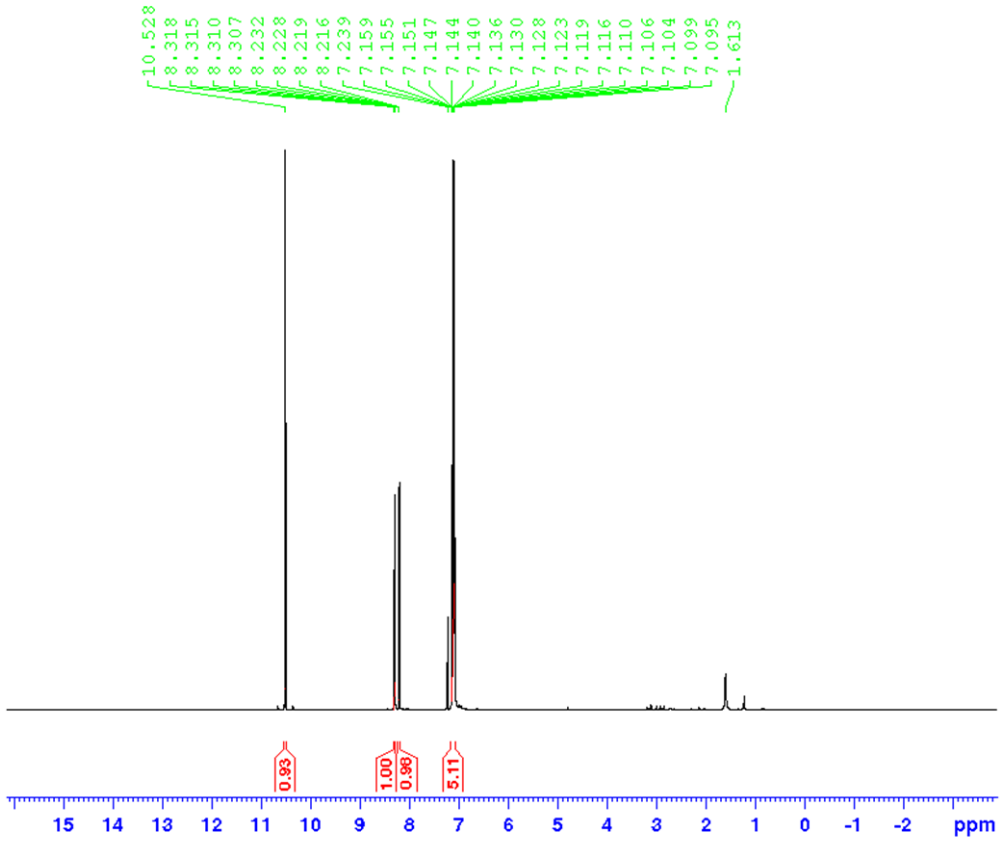


```
Current Data Parameters
NAME          ucvjc-zb-02
EXPNO                   1
PROCNO                  1

F2 - Acquisition Parameters
Date_            20170621
Time                10.35
INSTRUM             spect
PROBHD    5 mm PATXI 1H/
PULPROG              zg30
TD                  65536
SOLVENT             CDCl3
NS                     64
DS                      2
SWH            12019.230 Hz
FIDRES          0.183399 Hz
AQ             2.7262976 sec
RG                 115.18
DW                 41.600 usec
DE                   6.50 usec
TE                  298.1 K
D1             1.00000000 sec
TD0                     1

======== CHANNEL f1 ========
SFO1         600.0437055 MHz
NUC1                   1H
P1                   9.90 usec
PLW1           9.00000000 W

F2 - Processing parameters
SI                  65536
SF           600.0400262 MHz
WDW                    EM
SSB      0
LB                   0.30 Hz
GB       0
PC                   1.00
```

Figure S4

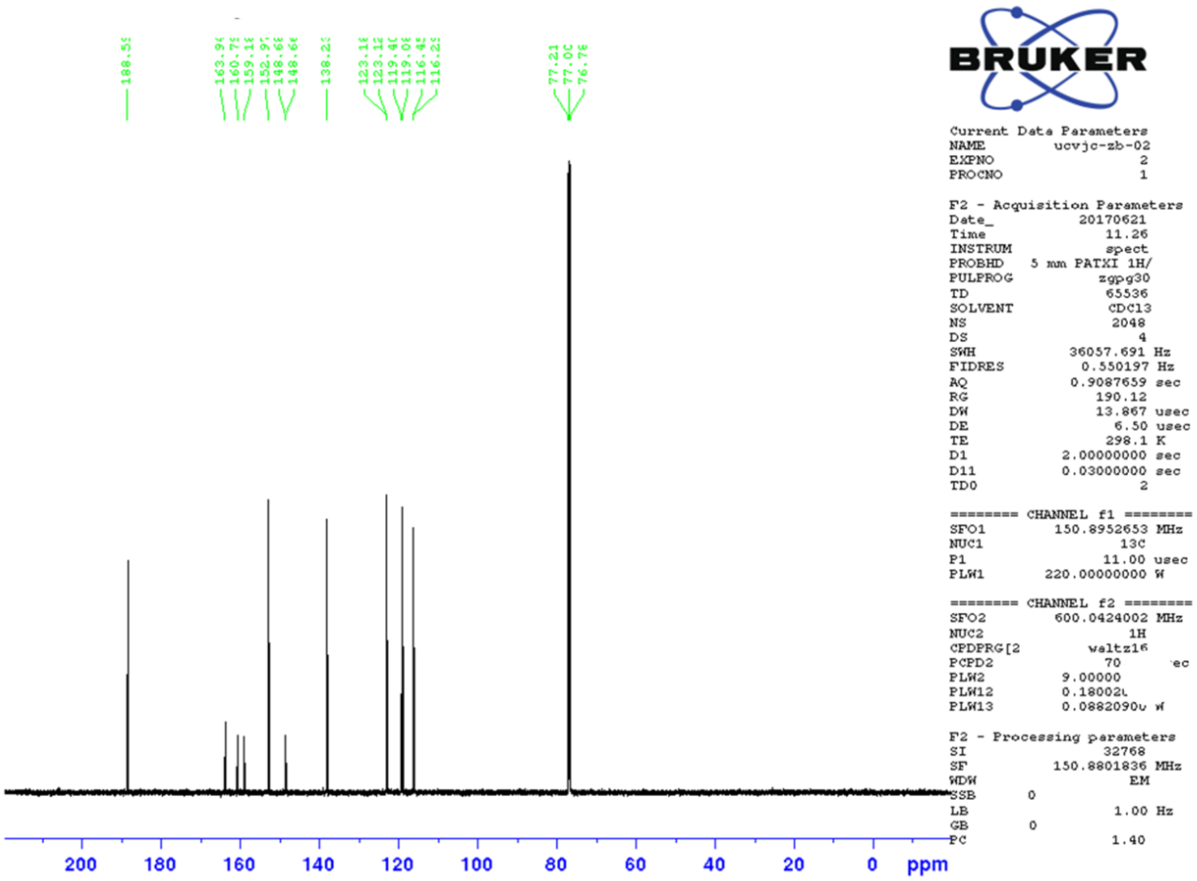
188.5
163.9
160.7
159.1
152.9
148.6
148.6
138.2
123.1
123.1
119.4
119.0
116.4
116.2
77.21
77.0
76.7
BRUKER
Current Data Parameters
NAME ucvjc-zb-02
EXPNO 2
PROCNO 1
F2 - Acquisition Parameters
Date_ 20170621
Time 11.26
INSTRUM spect
PROBHD 5 mm PATXI 1H/
PULPROG zgpg30
TD 65536
SOLVENT CDCl3
NS 2048
DS 4
SWH 36057.691 Hz
FIDRES 0.550197 Hz
AQ 0.9087659 sec
RG 190.12
DW 13.867 usec
DE 6.50 usec
TE 298.1 K
D1 2.00000000 sec
D11 0.03000000 sec
TD0 2
======== CHANNEL f1 ========
SFO1 150.8952653 MHz
NUC1 13C
P1 11.00 usec
PLW1 220.00000000 W
======== CHANNEL f2 ========
SFO2 600.0424002 MHz
NUC2 1H
CPDPRG[2 waltz16
PCPD2 70
PLW2 9.00000
PLW12 0.18002
PLW13 0.0882090 W
F2 - Processing parameters
SI 32768
SF 150.8801836 MHz
WDW EM
SSB 0
LB 1.00 Hz
GB 0
PC 1.40
200
180
160
140
120
100
80
60
40
20
0
ppm

**Figure S5**

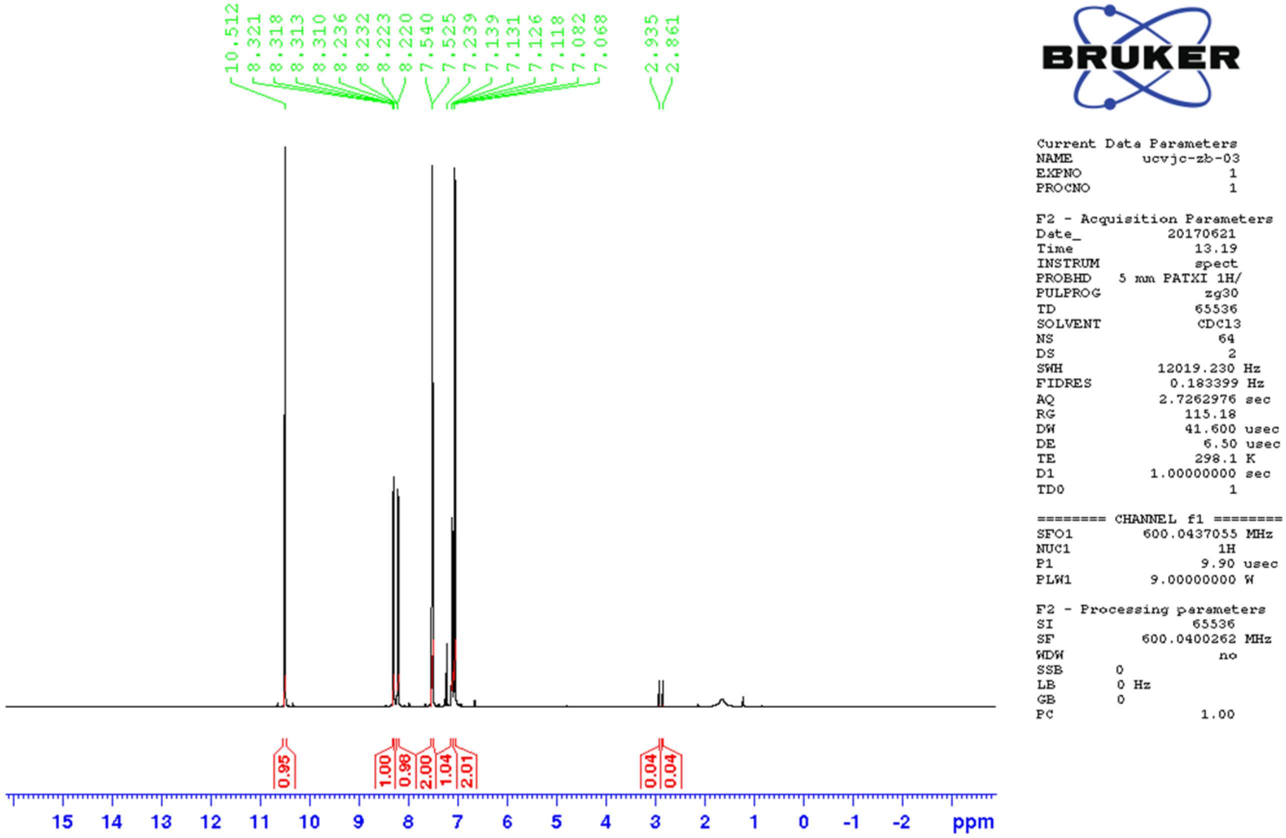

10.512
8.321
8.318
8.313
8.310
8.236
8.232
8.223
8.220
7.540
7.525
7.239
7.139
7.131
7.126
7.118
7.082
7.068
2.935
2.861
0.95
1.00
0.96
2.00
1.04
2.01
0.04
0.04
15 14 13 12 11 10 9 8 7 6 5 4 3 2 1 0 -1 -2 ppm
BRUKER
Current Data Parameters
NAME ucvjc-zb-03
EXPNO 1
PROCNO 1
F2 - Acquisition Parameters
Date_ 20170621
Time 13.19
INSTRUM spect
PROBHD 5 mm PATXI 1H/
PULPROG zg30
TD 65536
SOLVENT CDCl3
NS 64
DS 2
SWH 12019.230 Hz
FIDRES 0.183399 Hz
AQ 2.7262976 sec
RG 115.18
DW 41.600 usec
DE 6.50 usec
TE 298.1 K
D1 1.00000000 sec
TD0 1
======== CHANNEL f1 ========
SFO1 600.0437055 MHz
NUC1 1H
P1 9.90 usec
PLW1 9.00000000 W
F2 - Processing parameters
SI 65536
SF 600.0400262 MHz
WDW no
SSB 0
LB 0 Hz
GB 0
PC 1.00

**Figure S6**

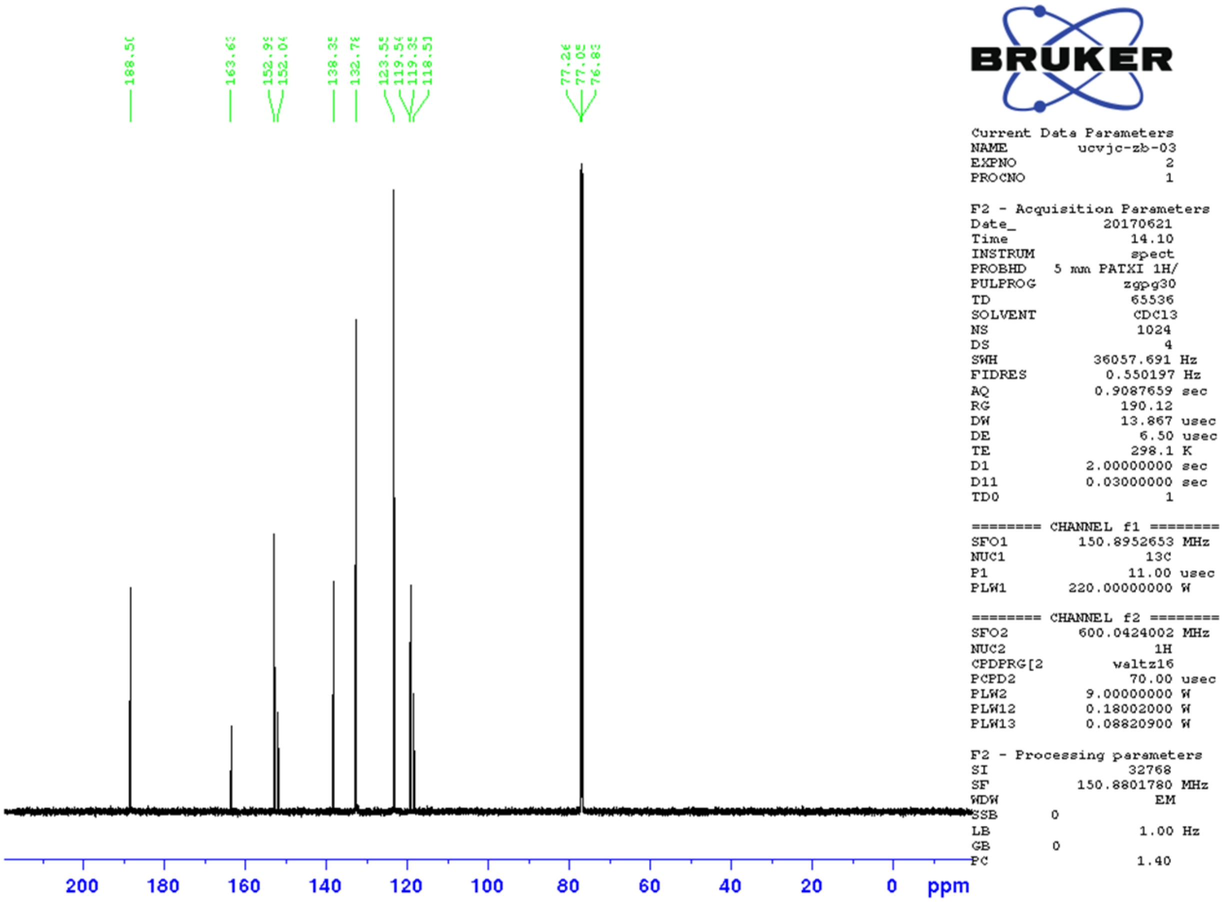

188.5
163.6
152.9
152.0
138.3
132.7
123.5
119.5
119.3
118.5
77.2
77.0
76.8
BRUKER
Current Data Parameters
NAME ucvjc-zb-03
EXPNO 2
PROCNO 1
F2 - Acquisition Parameters
Date_ 20170621
Time 14.10
INSTRUM spect
PROBHD 5 mm PATXI 1H/
PULPROG zgpg30
TD 65536
SOLVENT CDCl3
NS 1024
DS 4
SWH 36057.691 Hz
FIDRES 0.550197 Hz
AQ 0.9087659 sec
RG 190.12
DW 13.867 usec
DE 6.50 usec
TE 298.1 K
D1 2.00000000 sec
D11 0.03000000 sec
TD0 1
======== CHANNEL f1 ========
SFO1 150.8952653 MHz
NUC1 13C
P1 11.00 usec
PLW1 220.00000000 W
======== CHANNEL f2 ========
SFO2 600.0424002 MHz
NUC2 1H
CPDPRG[2 waltz16
PCPD2 70.00 usec
PLW2 9.00000000 W
PLW12 0.18002000 W
PLW13 0.08820900 W
F2 - Processing parameters
SI 32768
SF 150.8801780 MHz
WDW EM
SSB 0
LB 1.00 Hz
GB 0
PC 1.40
200
180
160
140
120
100
80
60
40
20
0
ppm

**Figure S7**

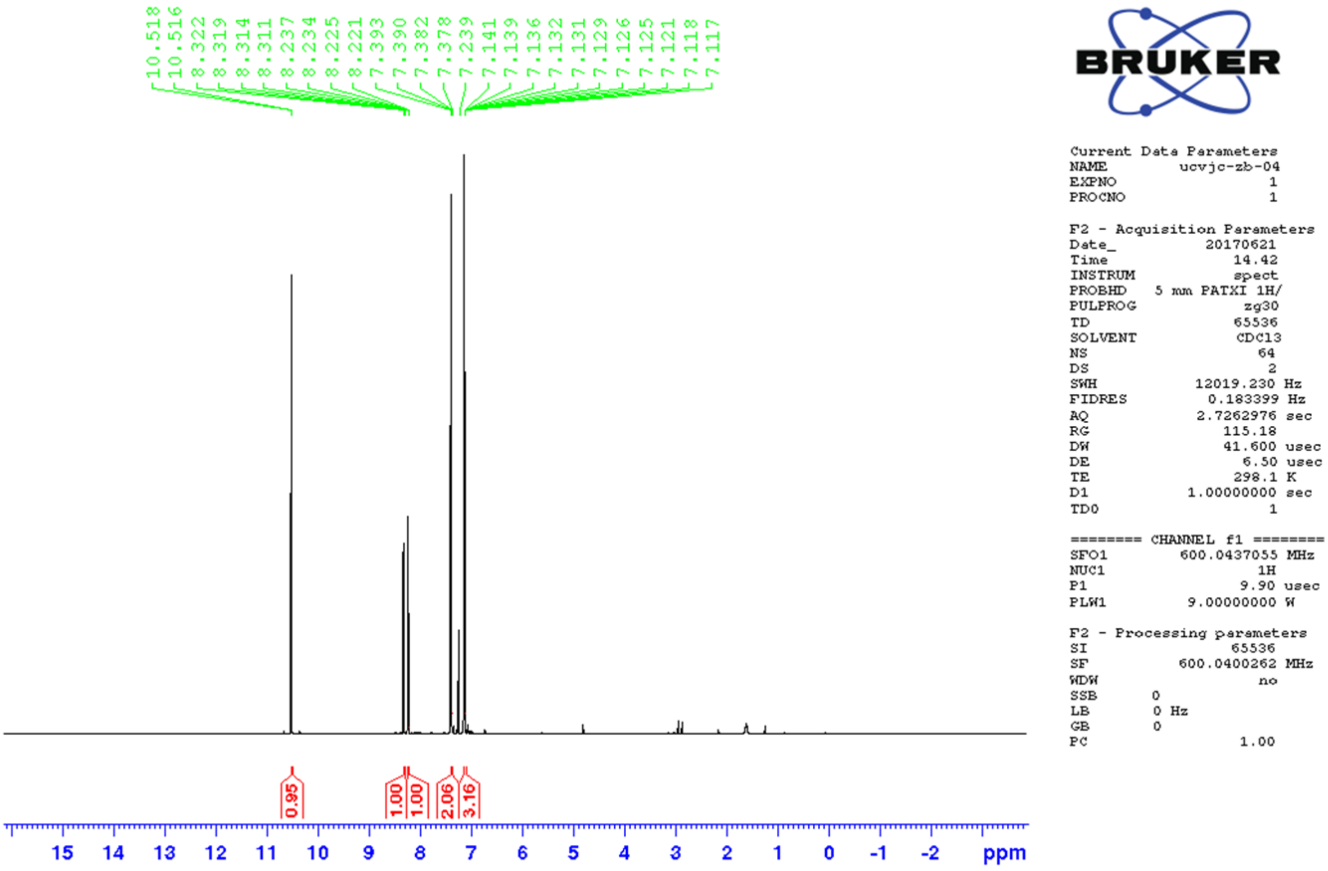
10.518
10.516
8.322
8.319
8.314
8.311
8.237
8.234
8.225
8.221
7.393
7.390
7.382
7.378
7.239
7.141
7.139
7.136
7.132
7.131
7.129
7.126
7.125
7.121
7.118
7.117
0.95
1.00
1.00
2.06
3.16
15 14 13 12 11 10 9 8 7 6 5 4 3 2 1 0 -1 -2 ppm
BRUKER
Current Data Parameters
NAME ucvjc-zb-04
EXPNO 1
PROCNO 1
F2 - Acquisition Parameters
Date_ 20170621
Time 14.42
INSTRUM spect
PROBHD 5 mm PATXI 1H/
PULPROG zg30
TD 65536
SOLVENT CDCl3
NS 64
DS 2
SWH 12019.230 Hz
FIDRES 0.183399 Hz
AQ 2.7262976 sec
RG 115.18
DW 41.600 usec
DE 6.50 usec
TE 298.1 K
D1 1.00000000 sec
TD0 1
======== CHANNEL f1 ========
SFO1 600.0437055 MHz
NUC1 1H
P1 9.90 usec
PLW1 9.00000000 W
F2 - Processing parameters
SI 65536
SF 600.0400262 MHz
WDW no
SSB 0
LB 0 Hz
GB 0
PC 1.00

**Figure S8**

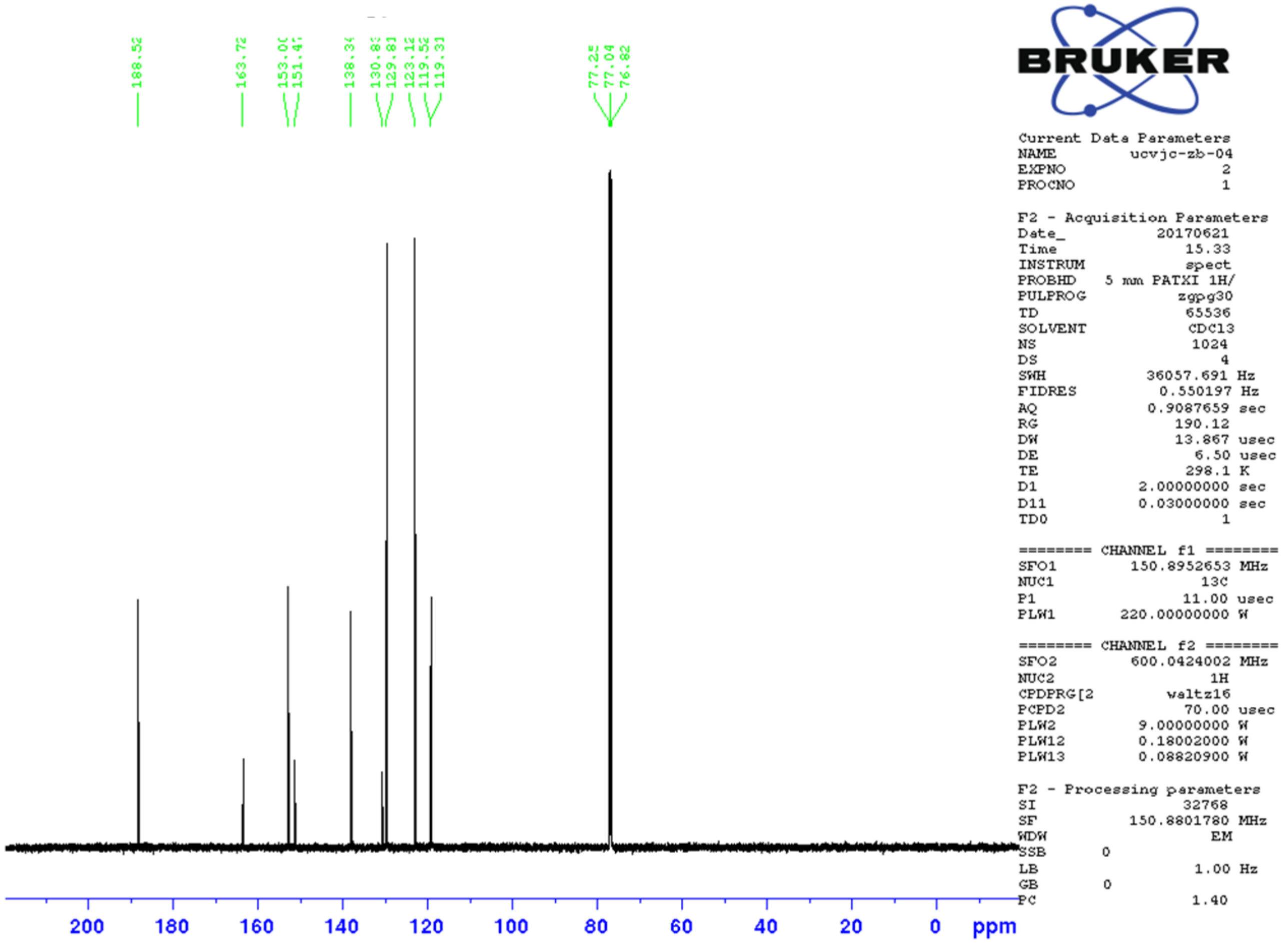
188.52
163.72
129.81
123.12
119.52
119.31
77.25
77.04
76.82
BRUKER
Current Data Parameters
NAME ucvjc-zb-04
EXPNO 2
PROCNO 1
F2 - Acquisition Parameters
Date_ 20170621
Time 15.33
INSTRUM spect
PROBHD 5 mm PATXI 1H/
PULPROG zgpg30
TD 65536
SOLVENT CDCl3
NS 1024
DS 4
SWH 36057.691 Hz
FIDRES 0.550197 Hz
AQ 0.9087659 sec
RG 190.12
DW 13.867 usec
DE 6.50 usec
TE 298.1 K
D1 2.00000000 sec
D11 0.03000000 sec
TD0 1
======== CHANNEL f1 ========
SFO1 150.8952653 MHz
NUC1 13C
P1 11.00 usec
PLW1 220.00000000 W
======== CHANNEL f2 ========
SFO2 600.0424002 MHz
NUC2 1H
CPDPRG[2 waltz16
PCPD2 70.00 usec
PLW2 9.00000000 W
PLW12 0.18002000 W
PLW13 0.08820900 W
F2 - Processing parameters
SI 32768
SF 150.8801780 MHz
WDW EM
SSB 0
LB 1.00 Hz
GB 0
PC 1.40
200
180
160
140
120
100
80
60
40
20
0
ppm

**Figure S9**

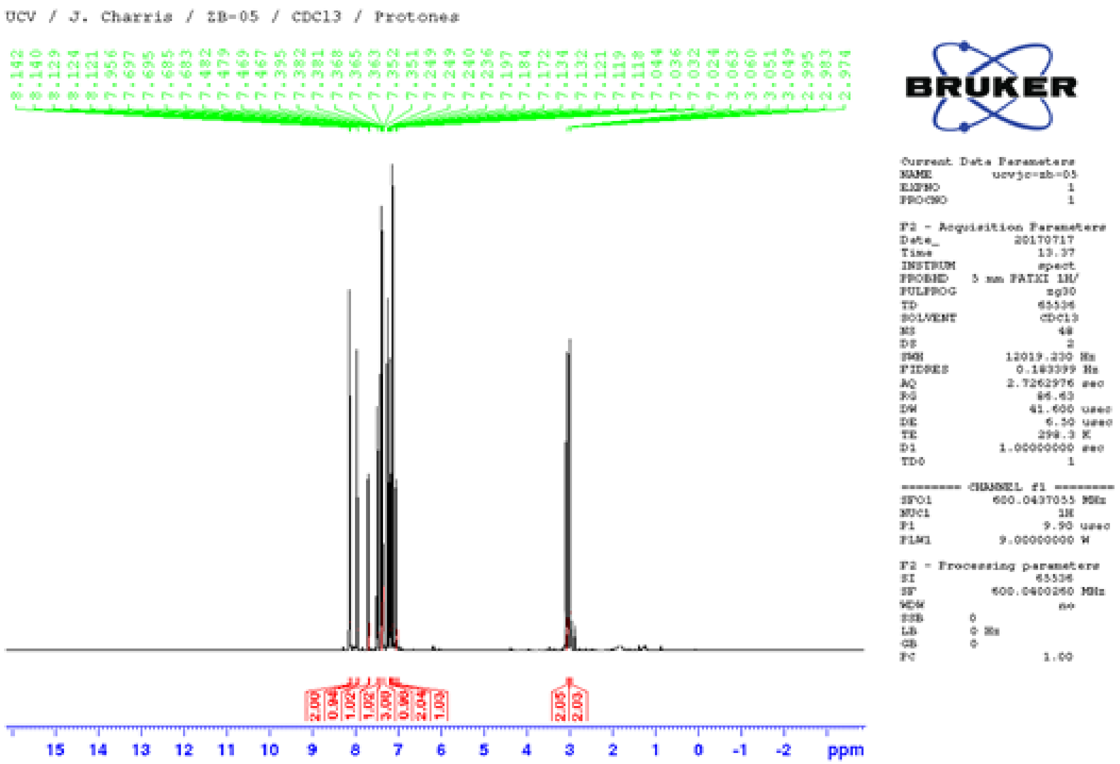

**Figure S10**

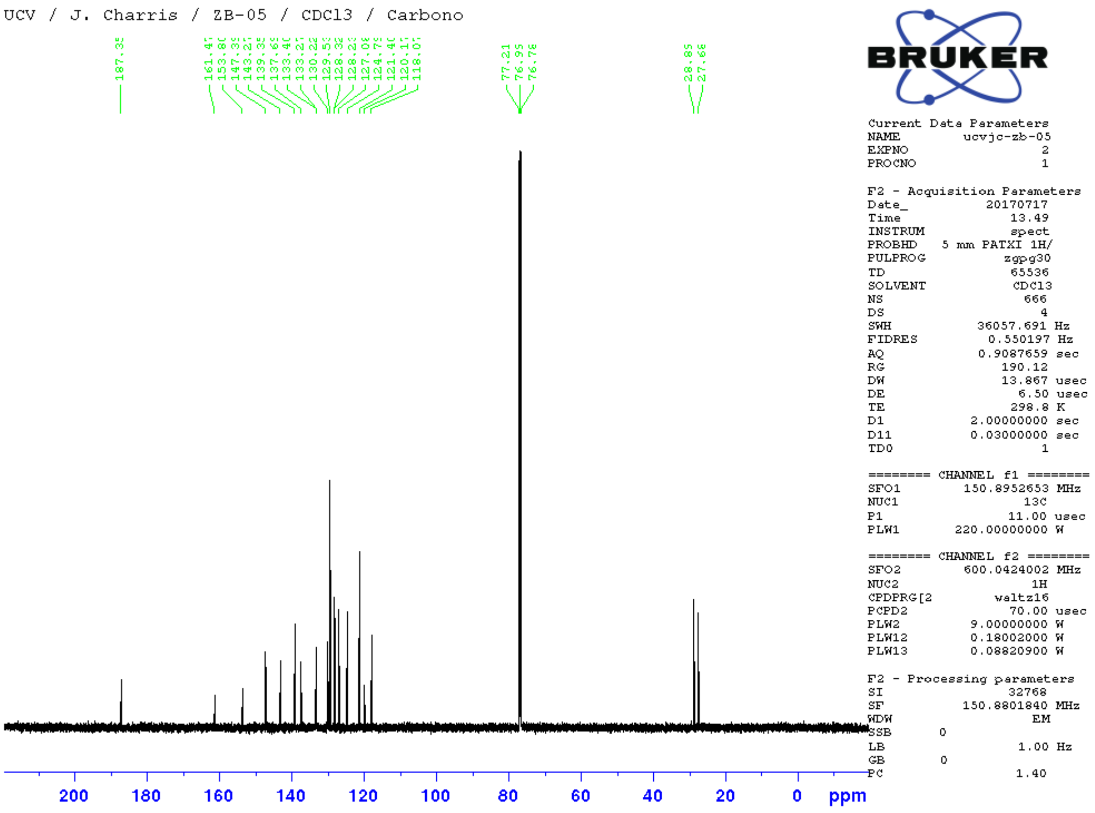
UCV / J. Charris / ZB-05 / CDCl3 / Carbono
BRUKER
Current Data Parameters
NAME ucvjc-zb-05
EXPNO 2
PROCNO 1
F2 - Acquisition Parameters
Date_ 20170717
Time 13.49
INSTRUM spect
PROBHD 5 mm PATXI 1H/
PULPROG zgpg30
TD 65536
SOLVENT CDCl3
NS 666
DS 4
SWH 36057.691 Hz
FIDRES 0.550197 Hz
AQ 0.9087659 sec
RG 190.12
DW 13.867 usec
DE 6.50 usec
TE 298.8 K
D1 2.00000000 sec
D11 0.03000000 sec
TD0 1
======== CHANNEL f1 ========
SFO1 150.8952653 MHz
NUC1 13C
P1 11.00 usec
PLW1 220.00000000 W
======== CHANNEL f2 ========
SFO2 600.0424002 MHz
NUC2 1H
CPDPRG[2 waltz16
PCPD2 70.00 usec
PLW2 9.00000000 W
PLW12 0.18002000 W
PLW13 0.08820900 W
F2 - Processing parameters
SI 32768
SF 150.8801840 MHz
WDW EM
SSB 0
LB 1.00 Hz
GB 0
PC 1.40
200 180 160 140 120 100 80 60 40 20 0 ppm

**Figure S11**

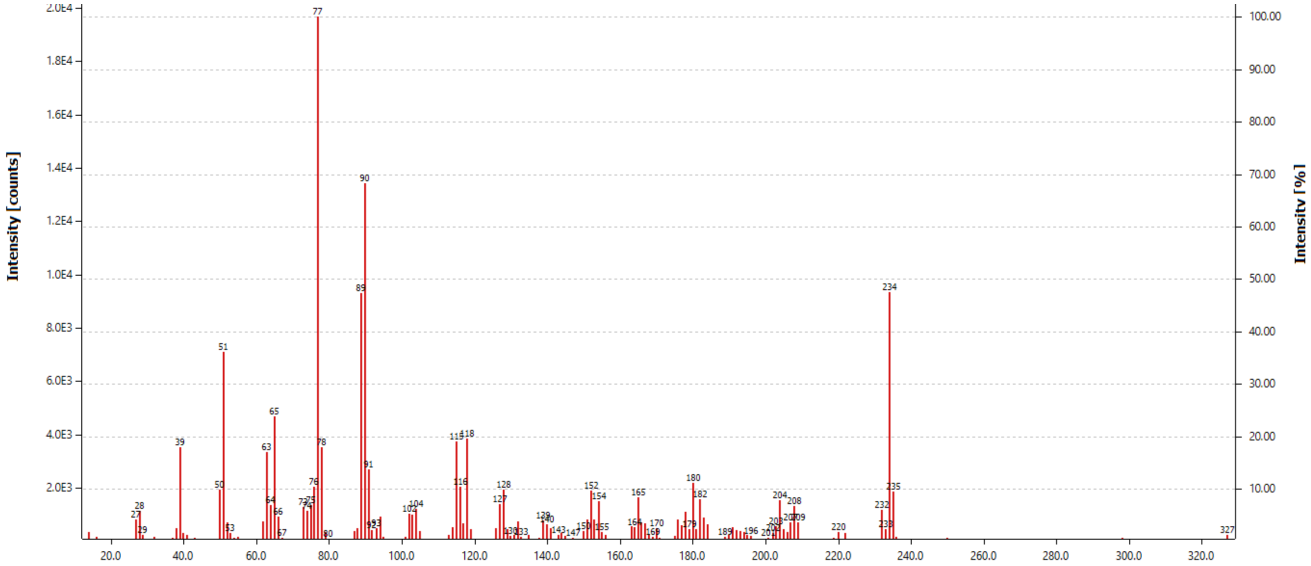

**Figure S12**

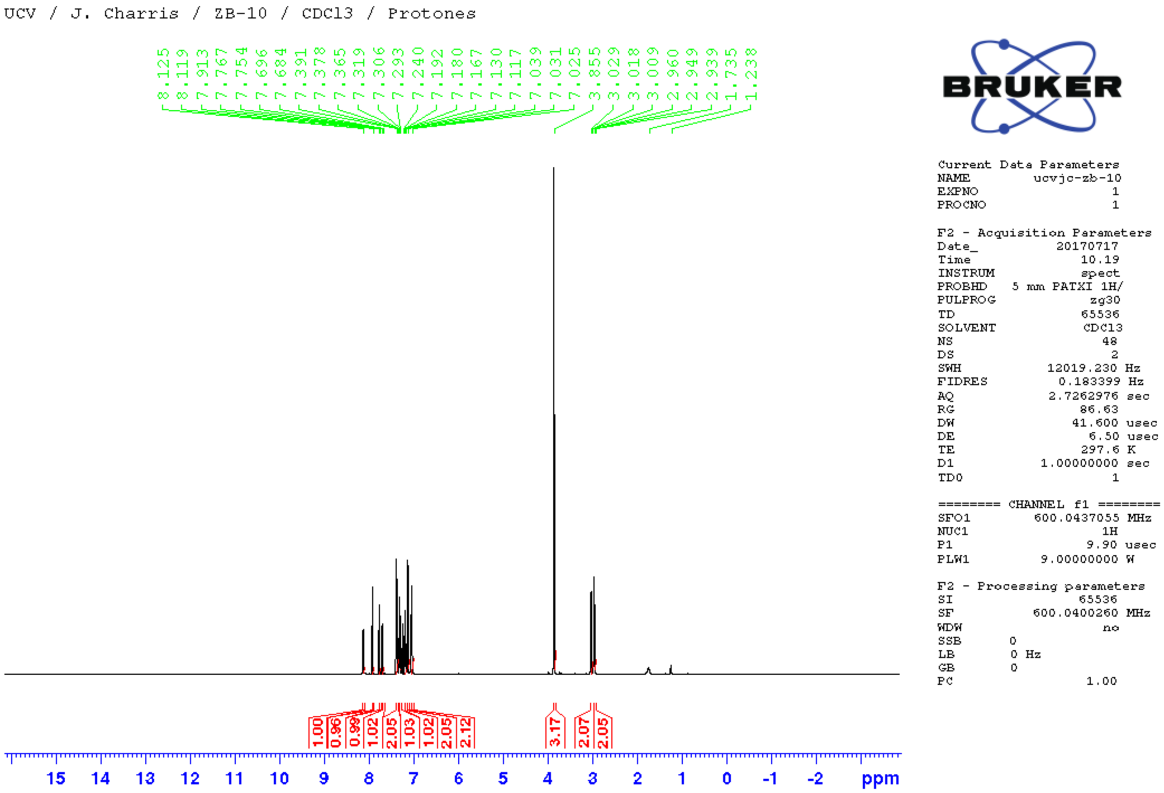
UCV / J. Charris / ZB-10 / CDCl3 / Protones
8.125
8.119
7.913
7.767
7.754
7.696
7.684
7.391
7.378
7.365
7.319
7.306
7.293
7.240
7.192
7.180
7.167
7.130
7.117
7.039
7.031
7.025
3.855
3.029
3.018
3.009
2.960
2.949
2.939
1.735
1.238
BRUKER
Current Data Parameters
NAME ucvjc-zb-10
EXPNO 1
PROCNO 1
F2 - Acquisition Parameters
Date_ 20170717
Time 10.19
INSTRUM spect
PROBHD 5 mm PATXI 1H/
PULPROG zg30
TD 65536
SOLVENT CDCl3
NS 48
DS 2
SWH 12019.230 Hz
FIDRES 0.183399 Hz
AQ 2.7262976 sec
RG 86.63
DW 41.600 usec
DE 6.50 usec
TE 297.6 K
D1 1.00000000 sec
TD0 1
======== CHANNEL f1 ========
SFO1 600.0437055 MHz
NUC1 1H
P1 9.90 usec
PLW1 9.00000000 W
F2 - Processing parameters
SI 65536
SF 600.0400260 MHz
WDW no
SSB 0
LB 0 Hz
GB 0
PC 1.00
1.00
0.96
0.99
1.02
2.05
1.03
1.02
2.05
2.12
3.17
2.07
2.05
15 14 13 12 11 10 9 8 7 6 5 4 3 2 1 0 -1 -2 ppm

**Figure S13**

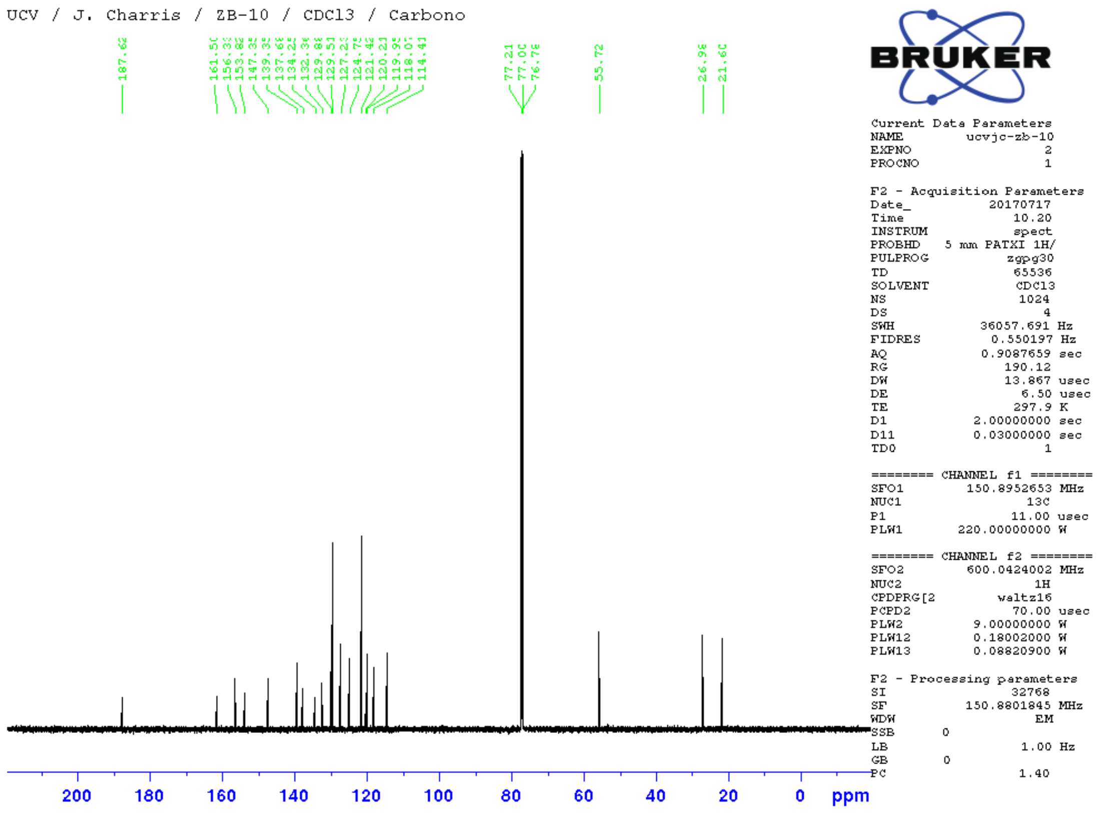
UCV / J. Charris / ZB-10 / CDCl3 / Carbono
187.62
161.50
153.82
129.51
124.75
121.42
120.21
114.41
77.21
77.00
76.78
55.72
21.60
BRUKER
Current Data Parameters
NAME ucvjc-zb-10
EXPNO 2
PROCNO 1
F2 - Acquisition Parameters
Date_ 20170717
Time 10.20
INSTRUM spect
PROBHD 5 mm PATXI 1H/
PULPROG zgpg30
TD 65536
SOLVENT CDCl3
NS 1024
DS 4
SWH 36057.691 Hz
FIDRES 0.550197 Hz
AQ 0.9087659 sec
RG 190.12
DW 13.867 usec
DE 6.50 usec
TE 297.9 K
D1 2.00000000 sec
D11 0.03000000 sec
TD0 1
======== CHANNEL f1 ========
SFO1 150.8952653 MHz
NUC1 13C
P1 11.00 usec
PLW1 220.00000000 W
======== CHANNEL f2 ========
SFO2 600.0424002 MHz
NUC2 1H
CPDPRG[2 waltz16
PCPD2 70.00 usec
PLW2 9.00000000 W
PLW12 0.18002000 W
PLW13 0.08820900 W
F2 - Processing parameters
SI 32768
SF 150.8801845 MHz
WDW EM
SSB 0
LB 1.00 Hz
GB 0
PC 1.40
200
180
160
140
120
100
80
60
40
20
0
ppm

**Figure S14**

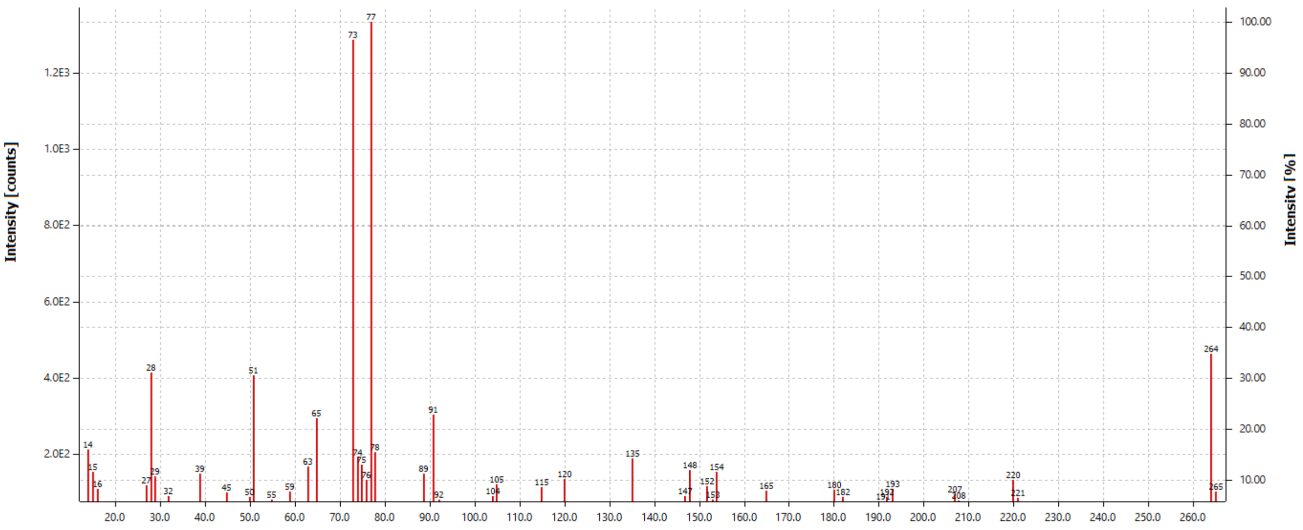

**Figure S15**

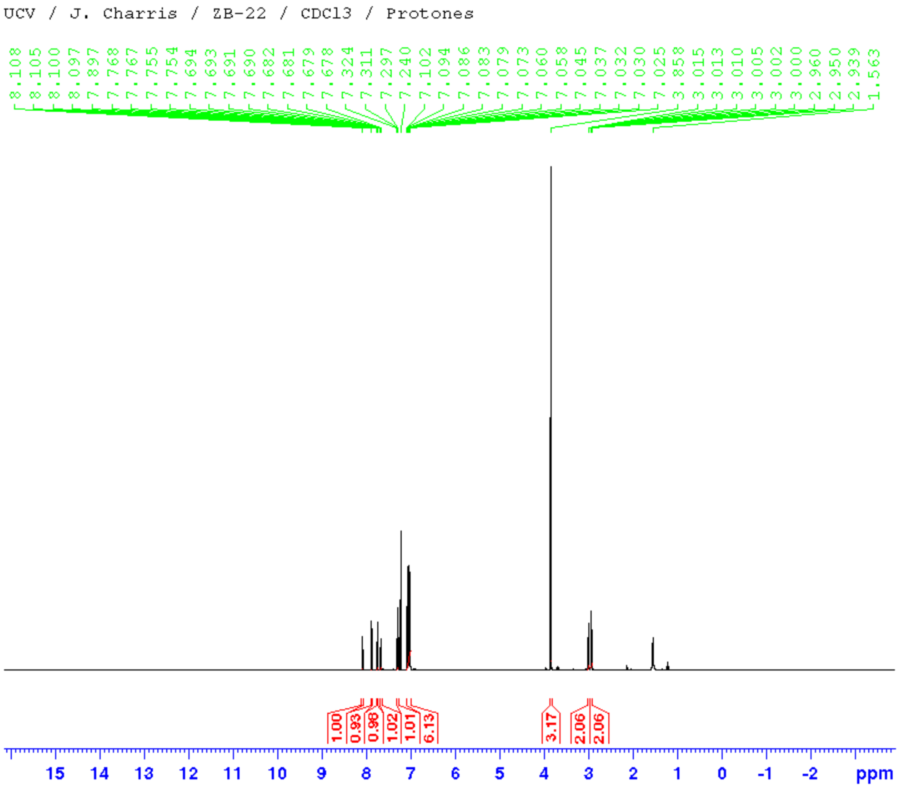


```
Current Data Parameters
NAME        ucvjch-zb-22
EXPNO                  1
PROCNO                 1

F2 - Acquisition Parameters
Date_           20180303
Time               10.30
INSTRUM            spect
PROBHD    5 mm PATXI 1H/
PULPROG             zg30
TD                 65536
SOLVENT            CDCl3
NS                    96
DS                     2
SWH            12019.230 Hz
FIDRES          0.183399 Hz
AQ             2.7262976 sec
RG                190.12
DW                41.600 usec
DE                  6.50 usec
TE                 298.1 K
D1            1.00000000 sec
TD0                    1

======== CHANNEL f1 ========
SFO1         600.0437055 MHz
NUC1                  1H
P1                  9.90 usec
PLW1          9.00000000 W

F2 - Processing parameters
SI                 65536
SF           600.0400262 MHz
WDW                   no
SSB       0
LB        0 Hz
GB        0
PC                  1.00
```

## Figure S16

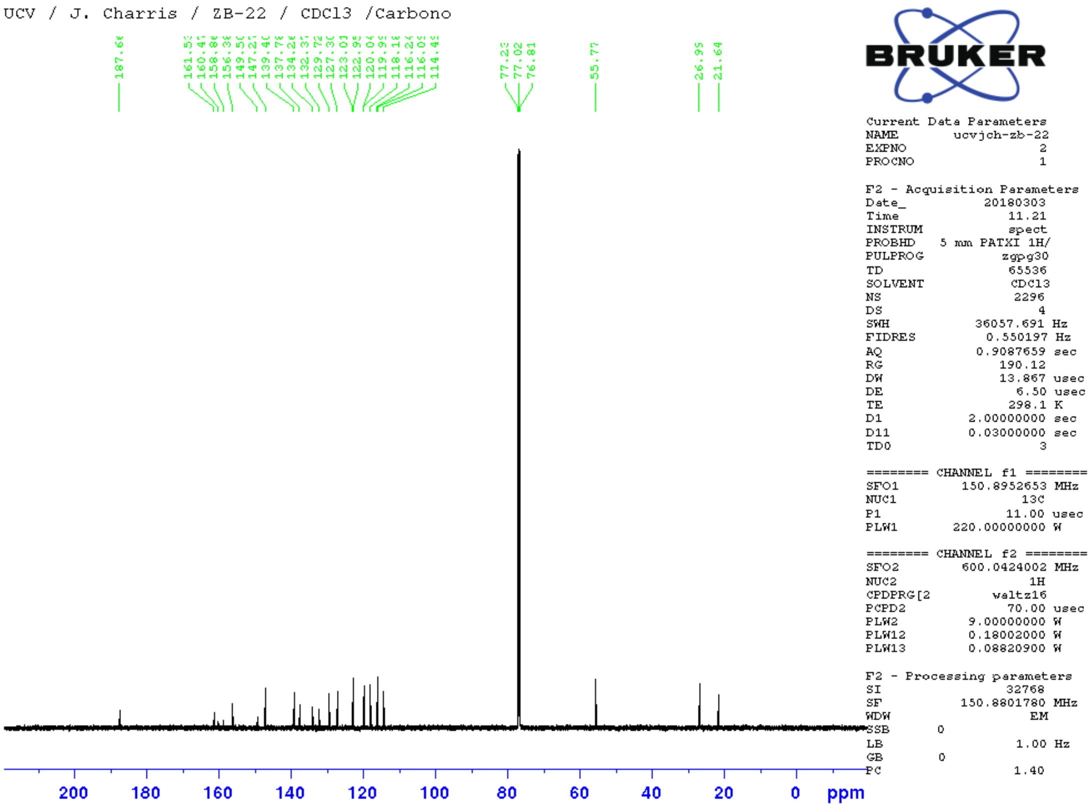

UCV / J. Charris / ZB-22 / CDCl3 /Carbono
77.23
77.02
76.81
55.77
26.95
21.64
BRUKER
Current Data Parameters
NAME ucvjch-zb-22
EXPNO 2
PROCNO 1
F2 - Acquisition Parameters
Date_ 20180303
Time 11.21
INSTRUM spect
PROBHD 5 mm PATXI 1H/
PULPROG zgpg30
TD 65536
SOLVENT CDCl3
NS 2296
DS 4
SWH 36057.691 Hz
FIDRES 0.550197 Hz
AQ 0.9087659 sec
RG 190.12
DW 13.867 usec
DE 6.50 usec
TE 298.1 K
D1 2.00000000 sec
D11 0.03000000 sec
TD0 3
======== CHANNEL f1 ========
SFO1 150.8952653 MHz
NUC1 13C
P1 11.00 usec
PLW1 220.00000000 W
======== CHANNEL f2 ========
SFO2 600.0424002 MHz
NUC2 1H
CPDPRG[2 waltz16
PCPD2 70.00 usec
PLW2 9.00000000 W
PLW12 0.18002000 W
PLW13 0.08820900 W
F2 - Processing parameters
SI 32768
SF 150.8801780 MHz
WDW EM
SSB 0
LB 1.00 Hz
GB 0
PC 1.40
200 180 160 140 120 100 80 60 40 20 0 ppm

**Figure S17**

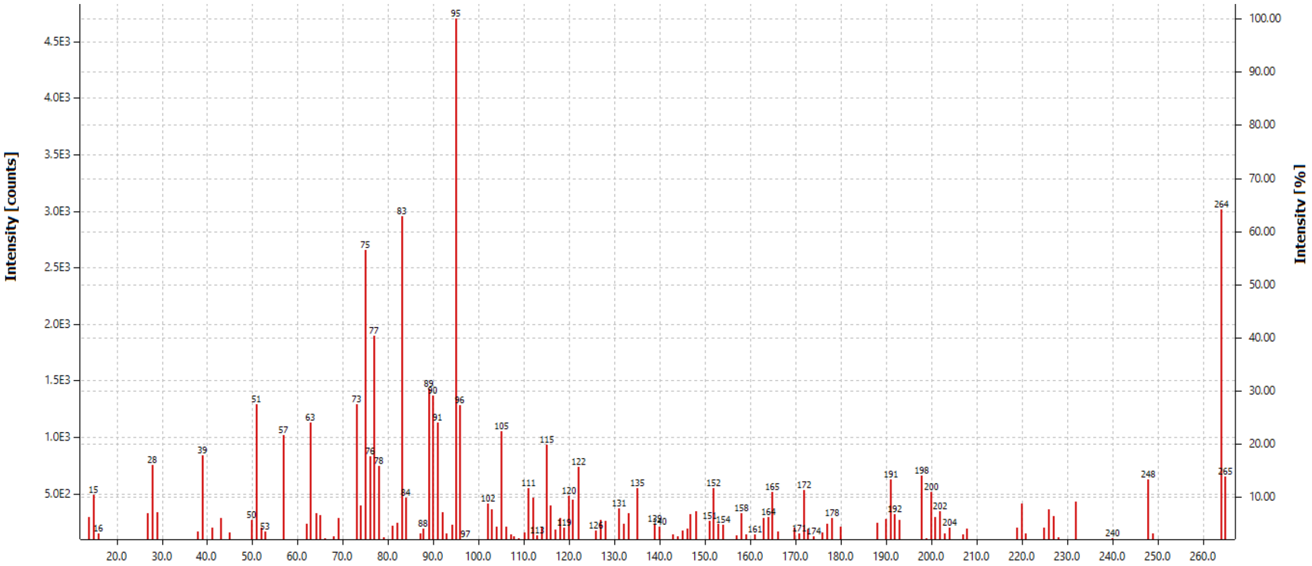
Intensity [counts]
Intensity [%]
4.5E3
4.0E3
3.5E3
3.0E3
2.5E3
2.0E3
1.5E3
1.0E3
5.0E2
100.00
90.00
80.00
70.00
60.00
50.00
40.00
30.00
20.00
10.00
20.0
30.0
40.0
50.0
60.0
70.0
80.0
90.0
100.0
110.0
120.0
130.0
140.0
150.0
160.0
170.0
180.0
190.0
200.0
210.0
220.0
230.0
240.0
250.0
260.0
95
83
75
77
89
90
96
73
51
63
57
91
105
115
122
264
265
248
191
198
200
192
202
204
240
39
28
15
16
50
53
76
78
84
88
97
102
111
120
119
126
131
135
139
140
151
152
154
158
161
164
165
172
174
178

**Figure S18**

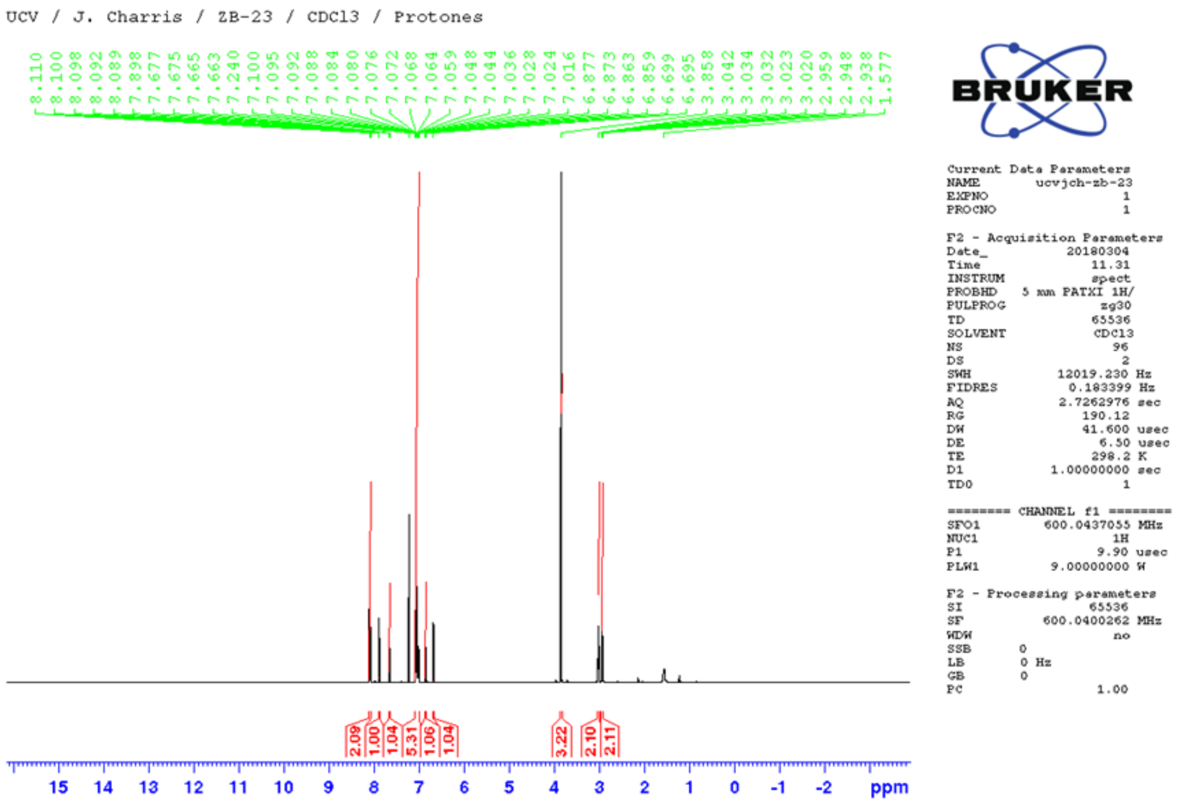

UCV / J. Charris / ZB-23 / CDCl3 / Protones
8.110
8.100
8.098
8.092
8.089
7.898
7.677
7.675
7.665
7.663
7.240
7.100
7.095
7.092
7.088
7.084
7.080
7.076
7.072
7.068
7.064
7.059
7.048
7.044
7.036
7.028
7.024
7.016
6.877
6.873
6.863
6.859
6.699
6.695
3.858
3.042
3.034
3.032
3.023
3.020
2.959
2.948
2.938
1.577
2.09
1.00
1.04
5.31
1.06
1.04
3.22
2.10
2.11
15
14
13
12
11
10
9
8
7
6
5
4
3
2
1
0
-1
-2
ppm
Current Data Parameters
NAME ucvjch-zb-23
EXPNO 1
PROCNO 1
F2 - Acquisition Parameters
Date_ 20180304
Time 11.31
INSTRUM spect
PROBHD 5 mm PATXI 1H/
PULPROG zg30
TD 65536
SOLVENT CDCl3
NS 96
DS 2
SWH 12019.230 Hz
FIDRES 0.183399 Hz
AQ 2.7262976 sec
RG 190.12
DW 41.600 usec
DE 6.50 usec
TE 298.2 K
D1 1.00000000 sec
TD0 1
======== CHANNEL f1 ========
SFO1 600.0437055 MHz
NUC1 1H
P1 9.90 usec
PLW1 9.00000000 W
F2 - Processing parameters
SI 65536
SF 600.0400262 MHz
WDW no
SSB 0
LB 0 Hz
GB 0
PC 1.00

**Figure S19**

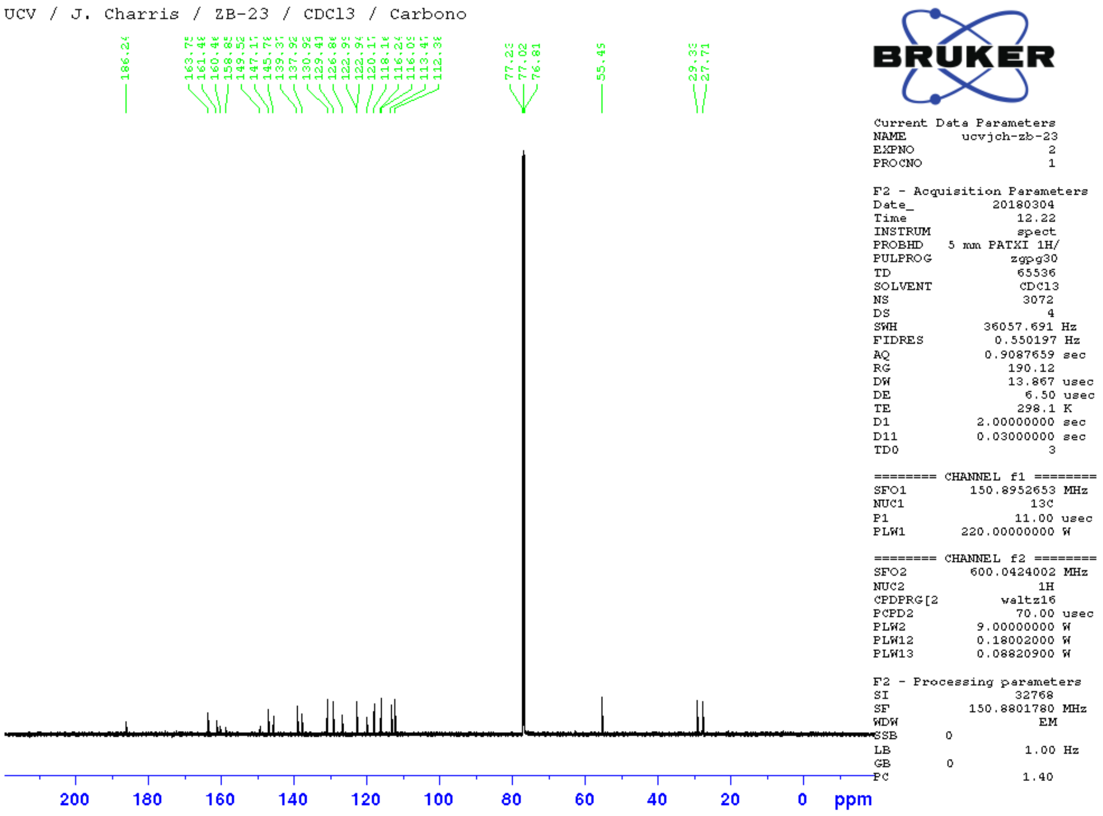
UCV / J. Charris / ZB-23 / CDCl3 / Carbono
77.23
77.02
76.81
55.45
BRUKER
Current Data Parameters
NAME ucvjch-zb-23
EXPNO 2
PROCNO 1
F2 - Acquisition Parameters
Date_ 20180304
Time 12.22
INSTRUM spect
PROBHD 5 mm PATXI 1H/
PULPROG zgpg30
TD 65536
SOLVENT CDCl3
NS 3072
DS 4
SWH 36057.691 Hz
FIDRES 0.550197 Hz
AQ 0.9087659 sec
RG 190.12
DW 13.867 usec
DE 6.50 usec
TE 298.1 K
D1 2.00000000 sec
D11 0.03000000 sec
TD0 3
======== CHANNEL f1 ========
SFO1 150.8952653 MHz
NUC1 13C
P1 11.00 usec
PLW1 220.00000000 W
======== CHANNEL f2 ========
SFO2 600.0424002 MHz
NUC2 1H
CPDPRG[2 waltz16
PCPD2 70.00 usec
PLW2 9.00000000 W
PLW12 0.18002000 W
PLW13 0.08820900 W
F2 - Processing parameters
SI 32768
SF 150.8801780 MHz
WDW EM
SSB 0
LB 1.00 Hz
GB 0
PC 1.40
200 180 160 140 120 100 80 60 40 20 0 ppm

**Figure S20**

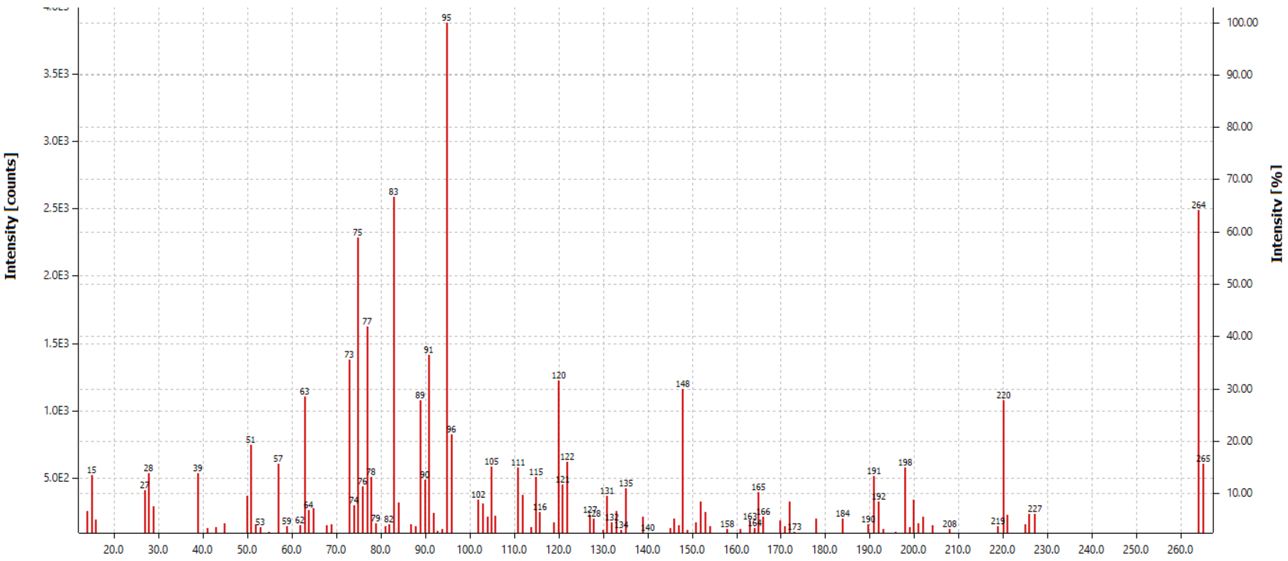

## Figure S21

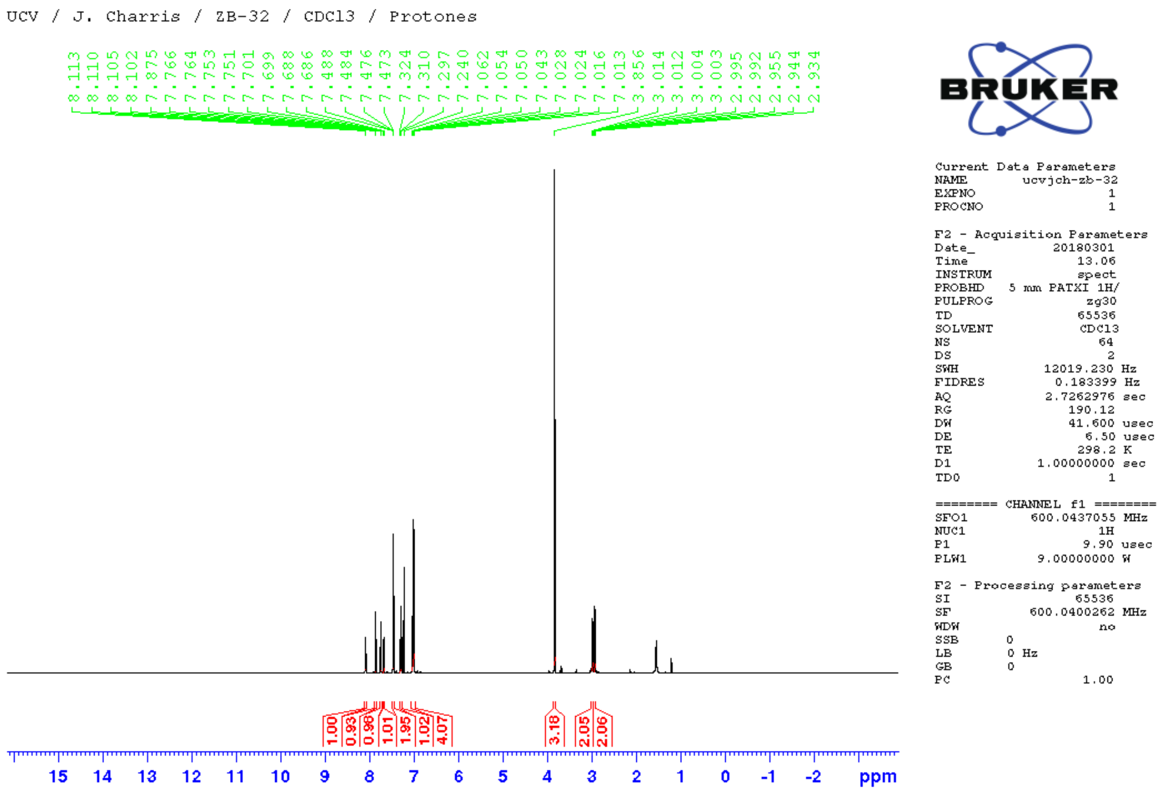
UCV / J. Charris / ZB-32 / CDCl3 / Protones
8.113
8.110
8.105
8.102
7.875
7.766
7.764
7.753
7.751
7.701
7.699
7.688
7.686
7.488
7.484
7.476
7.473
7.324
7.310
7.297
7.240
7.062
7.054
7.050
7.043
7.028
7.024
7.016
7.013
3.856
3.014
3.012
3.004
3.003
2.995
2.992
2.955
2.944
2.934
1.00
0.93
0.98
1.01
1.95
1.02
4.07
3.18
2.05
2.06
15 14 13 12 11 10 9 8 7 6 5 4 3 2 1 0 -1 -2 ppm
BRUKER
Current Data Parameters
NAME ucvjch-zb-32
EXPNO 1
PROCNO 1
F2 - Acquisition Parameters
Date_ 20180301
Time 13.06
INSTRUM spect
PROBHD 5 mm PATXI 1H/
PULPROG zg30
TD 65536
SOLVENT CDCl3
NS 64
DS 2
SWH 12019.230 Hz
FIDRES 0.183399 Hz
AQ 2.7262976 sec
RG 190.12
DW 41.600 usec
DE 6.50 usec
TE 298.2 K
D1 1.00000000 sec
TD0 1
======== CHANNEL f1 ========
SFO1 600.0437055 MHz
NUC1 1H
P1 9.90 usec
PLW1 9.00000000 W
F2 - Processing parameters
SI 65536
SF 600.0400262 MHz
WDW no
SSB 0
LB 0 Hz
GB 0
PC 1.00

**Figure S22**

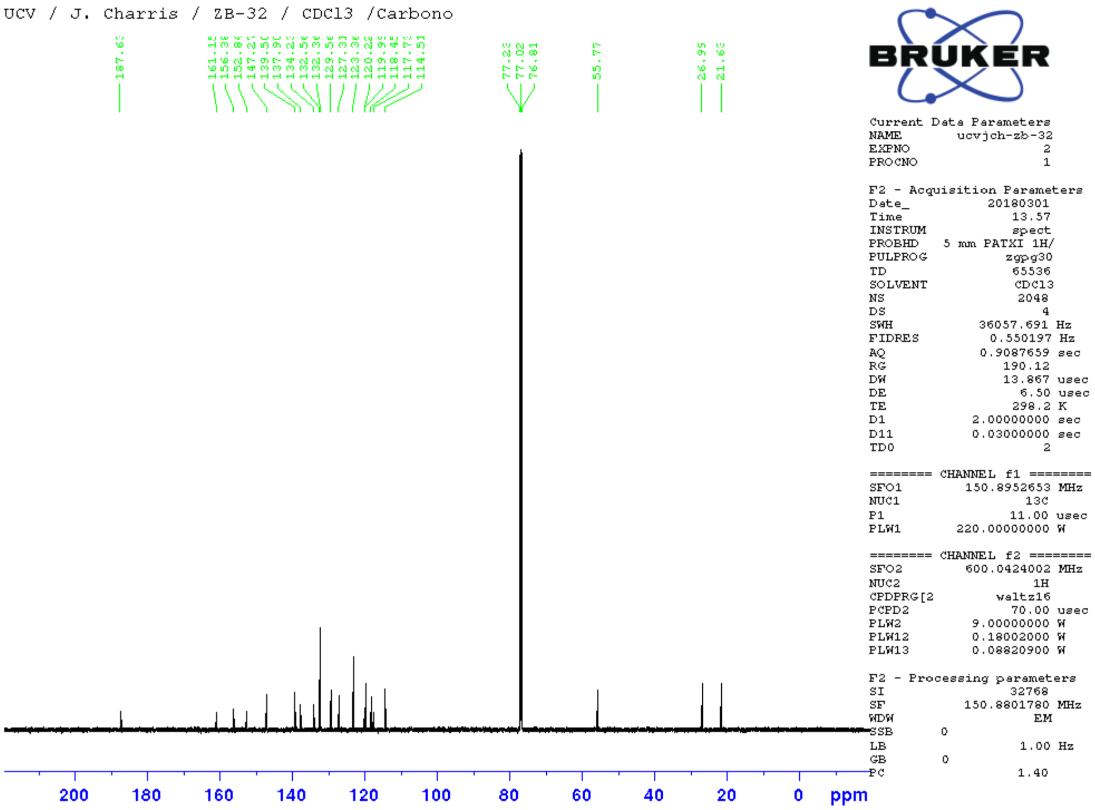
UCV / J. Charris / ZB-32 / CDCl3 /Carbono
Current Data Parameters
NAME ucvjch-zb-32
EXPNO 2
PROCNO 1
F2 - Acquisition Parameters
Date_ 20180301
Time 13.57
INSTRUM spect
PROBHD 5 mm PATXI 1H/
PULPROG zgpg30
TD 65536
SOLVENT CDCl3
NS 2048
DS 4
SWH 36057.691 Hz
FIDRES 0.550197 Hz
AQ 0.9087659 sec
RG 190.12
DW 13.867 usec
DE 6.50 usec
TE 298.2 K
D1 2.00000000 sec
D11 0.03000000 sec
TD0 2
======== CHANNEL f1 ========
SFO1 150.8952653 MHz
NUC1 13C
P1 11.00 usec
PLW1 220.00000000 W
======== CHANNEL f2 ========
SFO2 600.0424002 MHz
NUC2 1H
CPDPRG[2 waltz16
PCPD2 70.00 usec
PLW2 9.00000000 W
PLW12 0.18002000 W
PLW13 0.08820900 W
F2 - Processing parameters
SI 32768
SF 150.8801780 MHz
WDW EM
SSB 0
LB 1.00 Hz
GB 0
PC 1.40
200
180
160
140
120
100
80
60
40
20
0
ppm

**Figure S23**

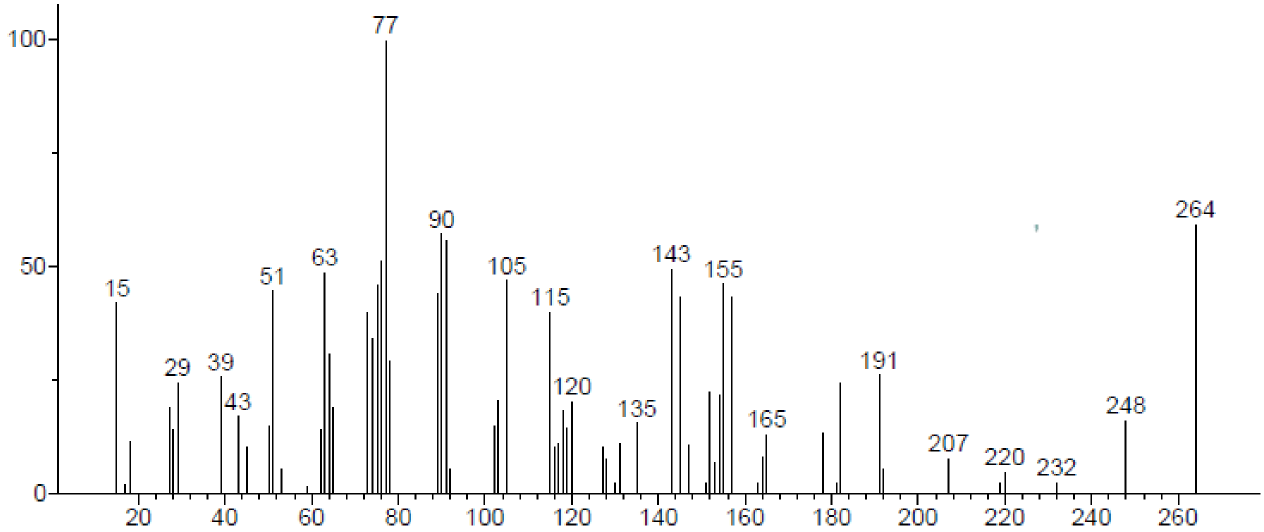

100
50
0
77
90
264
143
63
155
105
51
15
115
29
39
191
120
43
248
135
165
207
220
232
20
40
60
80
100
120
140
160
180
200
220
240
260

**Figure S24**

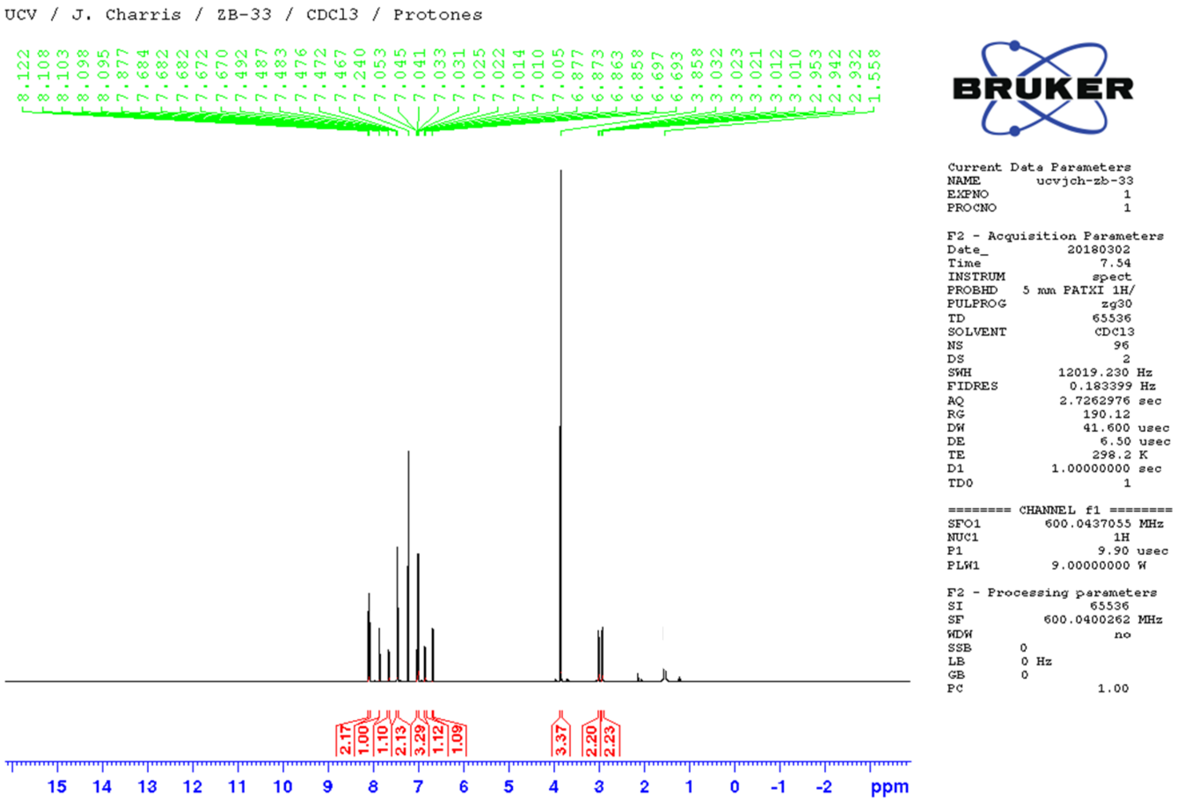
UCV / J. Charris / ZB-33 / CDCl3 / Protones
8.122 8.108 8.103 8.098 8.095 7.877 7.684 7.682 7.682 7.672 7.670 7.492 7.487 7.483 7.476 7.472 7.467 7.240 7.053 7.045 7.041 7.033 7.031 7.025 7.022 7.014 7.010 7.005 6.877 6.873 6.863 6.858 6.697 6.693 3.858 3.032 3.023 3.021 3.012 3.010 2.953 2.942 2.932 1.558
2.17 1.00 1.10 2.13 3.29 1.12 1.09 3.37 2.20 2.23
15 14 13 12 11 10 9 8 7 6 5 4 3 2 1 0 -1 -2 ppm
BRUKER
Current Data Parameters
NAME ucvjch-zb-33
EXPNO 1
PROCNO 1
F2 - Acquisition Parameters
Date_ 20180302
Time 7.54
INSTRUM spect
PROBHD 5 mm PATXI 1H/
PULPROG zg30
TD 65536
SOLVENT CDCl3
NS 96
DS 2
SWH 12019.230 Hz
FIDRES 0.183399 Hz
AQ 2.7262976 sec
RG 190.12
DW 41.600 usec
DE 6.50 usec
TE 298.2 K
D1 1.00000000 sec
TD0 1
======== CHANNEL f1 ========
SFO1 600.0437055 MHz
NUC1 1H
P1 9.90 usec
PLW1 9.00000000 W
F2 - Processing parameters
SI 65536
SF 600.0400262 MHz
WDW no
SSB 0
LB 0 Hz
GB 0
PC 1.00

**Figure S25**

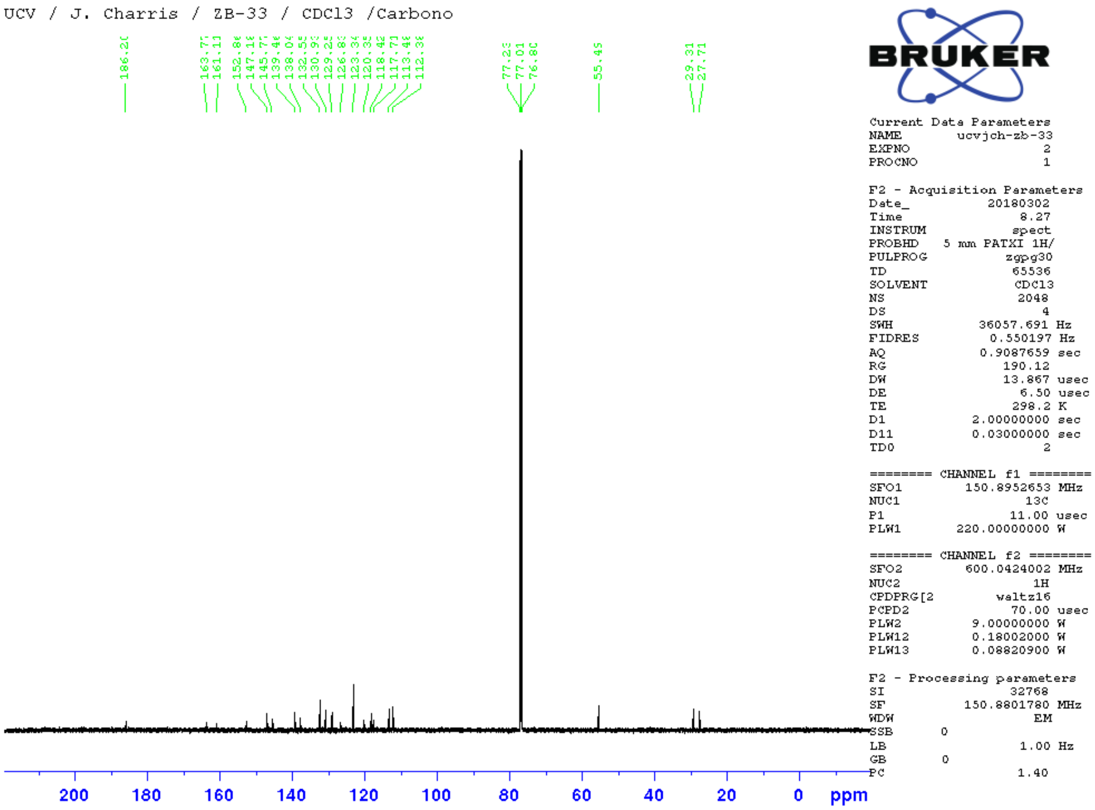
UCV / J. Charris / ZB-33 / CDCl3 /Carbono
Current Data Parameters
NAME ucvjch-zb-33
EXPNO 2
PROCNO 1
F2 - Acquisition Parameters
Date_ 20180302
Time 8.27
INSTRUM spect
PROBHD 5 mm PATXI 1H/
PULPROG zgpg30
TD 65536
SOLVENT CDCl3
NS 2048
DS 4
SWH 36057.691 Hz
FIDRES 0.550197 Hz
AQ 0.9087659 sec
RG 190.12
DW 13.867 usec
DE 6.50 usec
TE 298.2 K
D1 2.00000000 sec
D11 0.03000000 sec
TD0 2
======== CHANNEL f1 ========
SFO1 150.8952653 MHz
NUC1 13C
P1 11.00 usec
PLW1 220.00000000 W
======== CHANNEL f2 ========
SFO2 600.0424002 MHz
NUC2 1H
CPDPRG[2 waltz16
PCPD2 70.00 usec
PLW2 9.00000000 W
PLW12 0.18002000 W
PLW13 0.08820900 W
F2 - Processing parameters
SI 32768
SF 150.8801780 MHz
WDW EM
SSB 0
LB 1.00 Hz
GB 0
PC 1.40
200
180
160
140
120
100
80
60
40
20
0
ppm

**Figure S26**

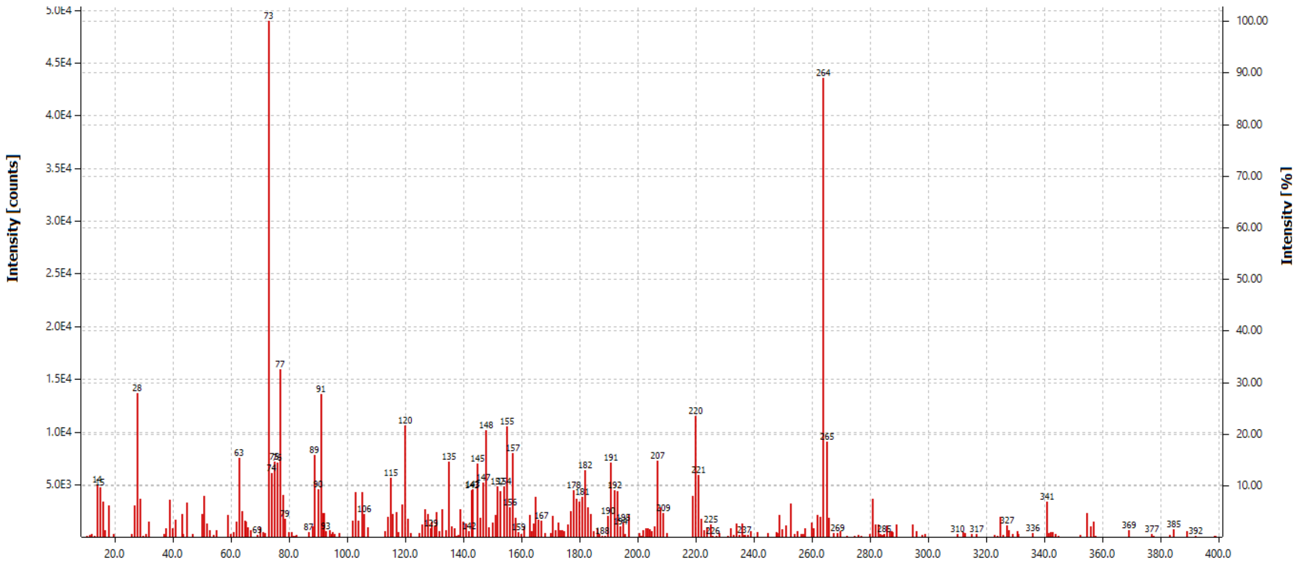

**Figure S27**

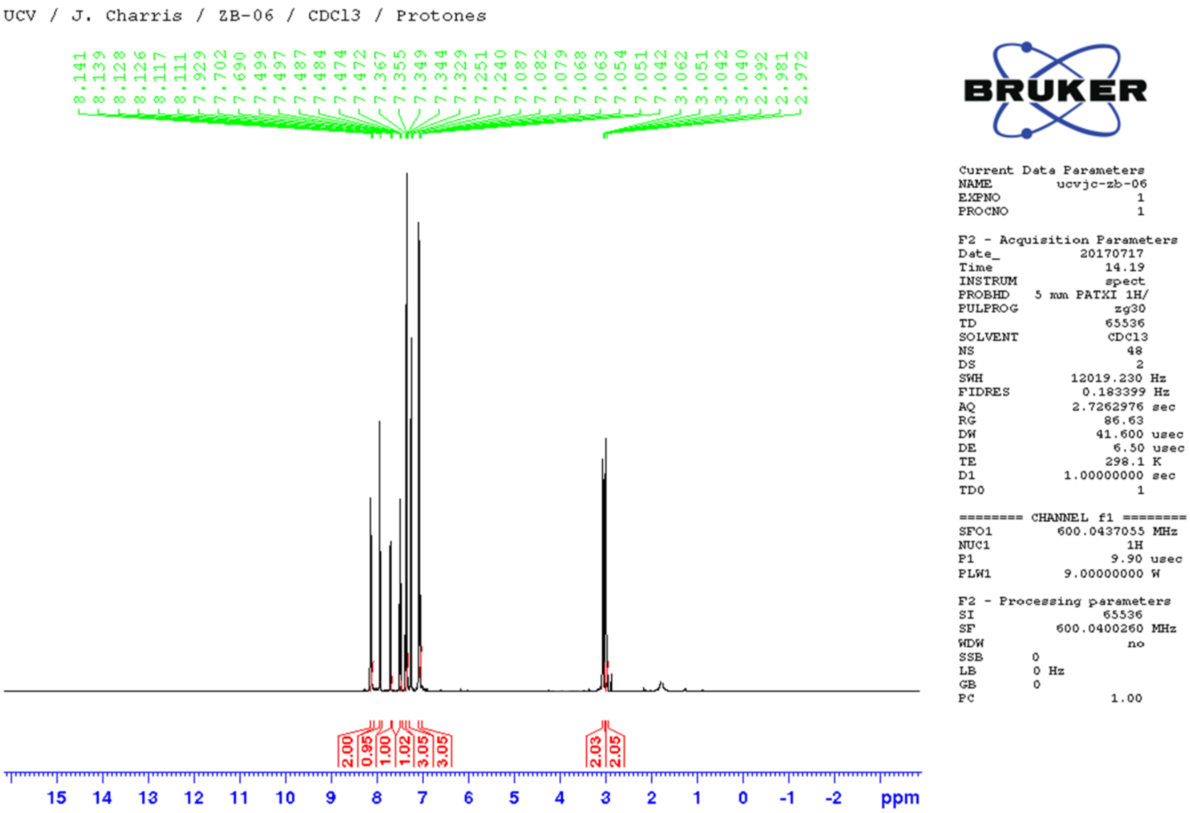
UCV / J. Charris / ZB-06 / CDCl3 / Protones
8.141
8.139
8.128
8.126
8.117
8.111
7.929
7.702
7.690
7.499
7.497
7.487
7.484
7.474
7.472
7.367
7.355
7.349
7.344
7.329
7.251
7.240
7.087
7.082
7.079
7.068
7.063
7.054
7.051
7.042
3.062
3.051
3.042
3.040
2.992
2.981
2.972
BRUKER
Current Data Parameters
NAME ucvjc-zb-06
EXPNO 1
PROCNO 1
F2 - Acquisition Parameters
Date_ 20170717
Time 14.19
INSTRUM spect
PROBHD 5 mm PATXI 1H/
PULPROG zg30
TD 65536
SOLVENT CDCl3
NS 48
DS 2
SWH 12019.230 Hz
FIDRES 0.183399 Hz
AQ 2.7262976 sec
RG 86.63
DW 41.600 usec
DE 6.50 usec
TE 298.1 K
D1 1.00000000 sec
TD0 1
======== CHANNEL f1 ========
SFO1 600.0437055 MHz
NUC1 1H
P1 9.90 usec
PLW1 9.00000000 W
F2 - Processing parameters
SI 65536
SF 600.0400260 MHz
WDW no
SSB 0
LB 0 Hz
GB 0
PC 1.00
2.00
0.95
1.00
1.02
3.05
3.05
2.03
2.05
15
14
13
12
11
10
9
8
7
6
5
4
3
2
1
0
-1
-2
ppm

**Figure S28**

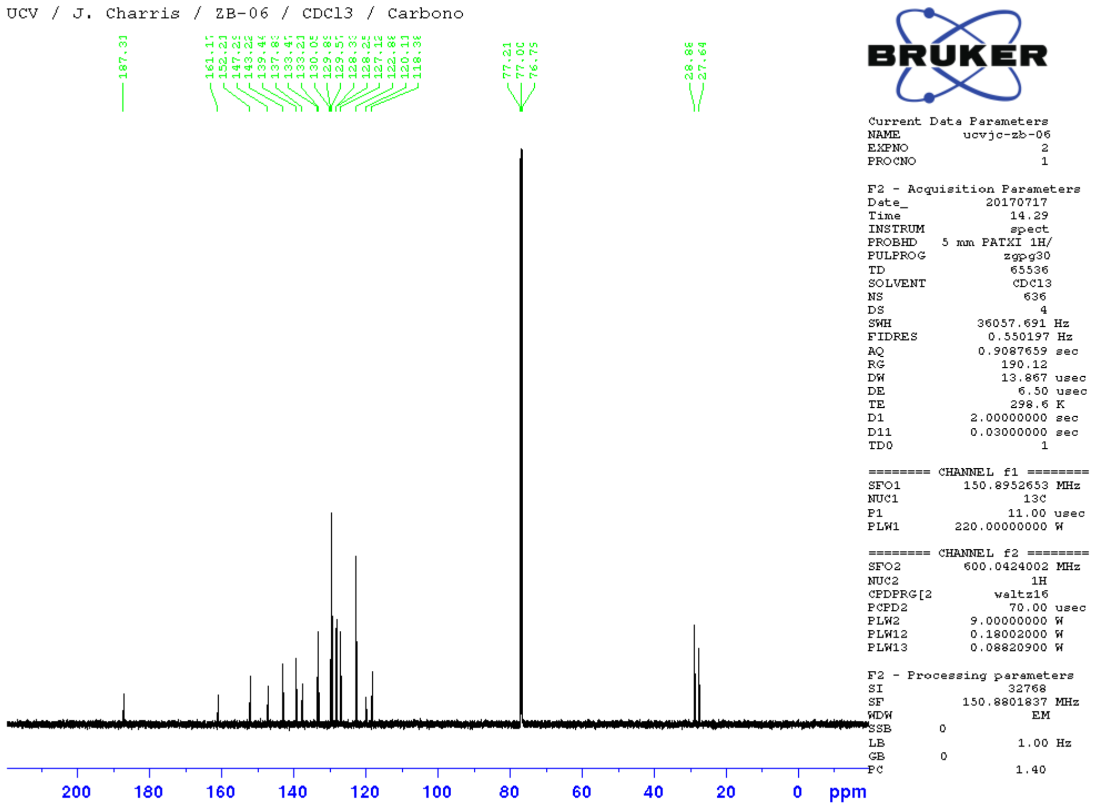

UCV / J. Charris / ZB-06 / CDCl3 / Carbono
187.31
77.21
77.00
76.79
28.86
27.64
BRUKER
Current Data Parameters
NAME ucvjc-zb-06
EXPNO 2
PROCNO 1
F2 - Acquisition Parameters
Date_ 20170717
Time 14.29
INSTRUM spect
PROBHD 5 mm PATXI 1H/
PULPROG zgpg30
TD 65536
SOLVENT CDCl3
NS 636
DS 4
SWH 36057.691 Hz
FIDRES 0.550197 Hz
AQ 0.9087659 sec
RG 190.12
DW 13.867 usec
DE 6.50 usec
TE 298.6 K
D1 2.00000000 sec
D11 0.03000000 sec
TD0 1
======== CHANNEL f1 ========
SFO1 150.8952653 MHz
NUC1 13C
P1 11.00 usec
PLW1 220.00000000 W
======== CHANNEL f2 ========
SFO2 600.0424002 MHz
NUC2 1H
CPDPRG[2 waltz16
PCPD2 70.00 usec
PLW2 9.00000000 W
PLW12 0.18002000 W
PLW13 0.08820900 W
F2 - Processing parameters
SI 32768
SF 150.8801837 MHz
WDW EM
SSB 0
LB 1.00 Hz
GB 0
PC 1.40
200 180 160 140 120 100 80 60 40 20 0 ppm

## Figure S29

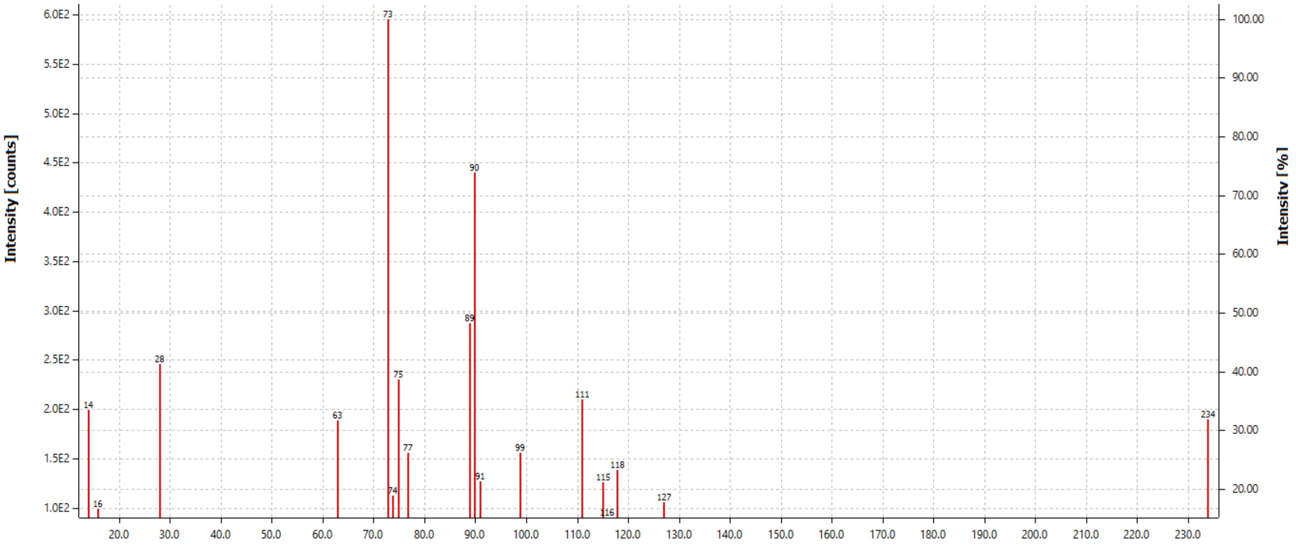

Intensity [counts]
Intensity [%]
6.0E2
5.5E2
5.0E2
4.5E2
4.0E2
3.5E2
3.0E2
2.5E2
2.0E2
1.5E2
1.0E2
100.00
90.00
80.00
70.00
60.00
50.00
40.00
30.00
20.00
20.0
30.0
40.0
50.0
60.0
70.0
80.0
90.0
100.0
110.0
120.0
130.0
140.0
150.0
160.0
170.0
180.0
190.0
200.0
210.0
220.0
230.0
14
16
28
63
73
74
75
77
89
90
91
99
111
115
116
118
127
234

**Figure S30**

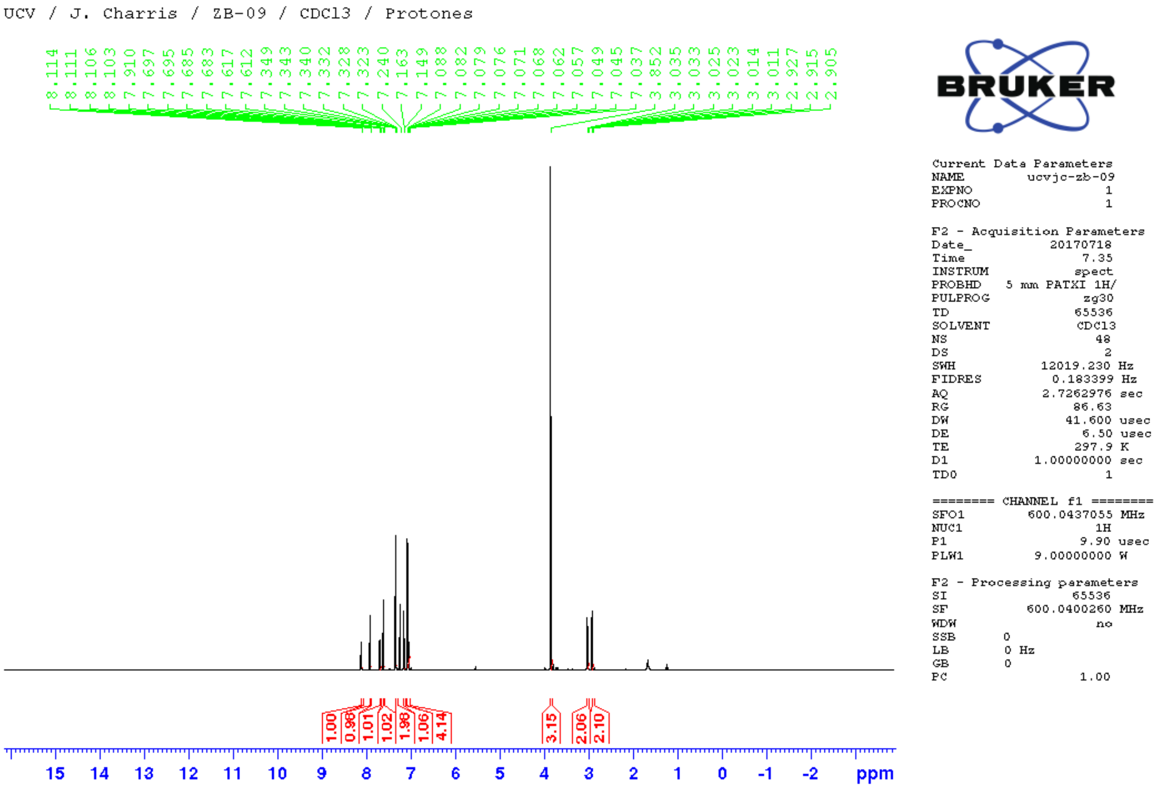

UCV / J. Charris / ZB-09 / CDCl3 / Protones
8.114
8.111
8.106
8.103
7.910
7.697
7.695
7.685
7.683
7.617
7.612
7.349
7.343
7.340
7.332
7.328
7.323
7.240
7.163
7.149
7.088
7.082
7.079
7.076
7.071
7.068
7.062
7.057
7.049
7.045
7.037
3.852
3.035
3.033
3.025
3.023
3.014
3.011
2.927
2.915
2.905
1.00
0.96
1.01
1.02
1.98
1.06
4.14
3.15
2.06
2.10
15 14 13 12 11 10 9 8 7 6 5 4 3 2 1 0 -1 -2 ppm
Current Data Parameters
NAME ucvjc-zb-09
EXPNO 1
PROCNO 1
F2 - Acquisition Parameters
Date_ 20170718
Time 7.35
INSTRUM spect
PROBHD 5 mm PATXI 1H/
PULPROG zg30
TD 65536
SOLVENT CDCl3
NS 48
DS 2
SWH 12019.230 Hz
FIDRES 0.183399 Hz
AQ 2.7262976 sec
RG 86.63
DW 41.600 usec
DE 6.50 usec
TE 297.9 K
D1 1.00000000 sec
TD0 1
======== CHANNEL f1 ========
SFO1 600.0437055 MHz
NUC1 1H
P1 9.90 usec
PLW1 9.00000000 W
F2 - Processing parameters
SI 65536
SF 600.0400260 MHz
WDW no
SSB 0
LB 0 Hz
GB 0
PC 1.00

**Figure S31**

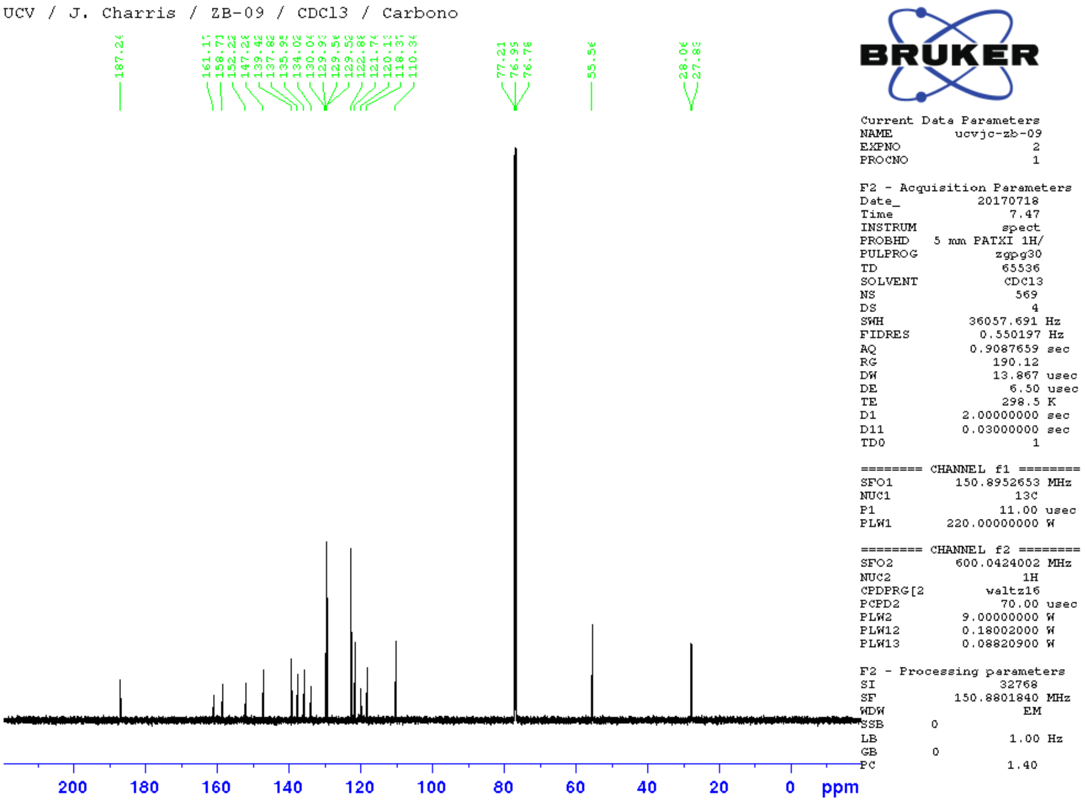

UCV / J. Charris / ZB-09 / CDCl3 / Carbono
BRUKER
Current Data Parameters
NAME ucvjc-zb-09
EXPNO 2
PROCNO 1
F2 - Acquisition Parameters
Date_ 20170718
Time 7.47
INSTRUM spect
PROBHD 5 mm PATXI 1H/
PULPROG zgpg30
TD 65536
SOLVENT CDCl3
NS 569
DS 4
SWH 36057.691 Hz
FIDRES 0.550197 Hz
AQ 0.9087659 sec
RG 190.12
DW 13.867 usec
DE 6.50 usec
TE 298.5 K
D1 2.00000000 sec
D11 0.03000000 sec
TD0 1
======== CHANNEL f1 ========
SFO1 150.8952653 MHz
NUC1 13C
P1 11.00 usec
PLW1 220.00000000 W
======== CHANNEL f2 ========
SFO2 600.0424002 MHz
NUC2 1H
CPDPRG[2 waltz16
PCPD2 70.00 usec
PLW2 9.00000000 W
PLW12 0.18002000 W
PLW13 0.08820900 W
F2 - Processing parameters
SI 32768
SF 150.8801840 MHz
WDW EM
SSB 0
LB 1.00 Hz
GB 0
PC 1.40
200
180
160
140
120
100
80
60
40
20
0
ppm

**Figure S32**

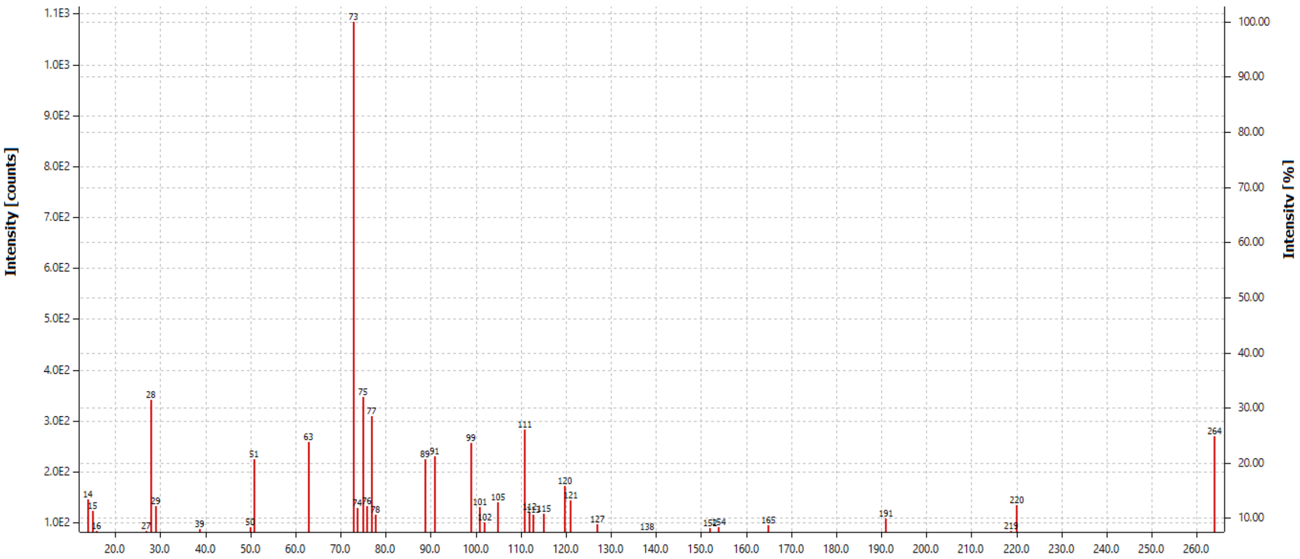

Intensity [counts]
Intensity [%]
1.1E3
1.0E3
9.0E2
8.0E2
7.0E2
6.0E2
5.0E2
4.0E2
3.0E2
2.0E2
1.0E2
100.00
90.00
80.00
70.00
60.00
50.00
40.00
30.00
20.00
10.00
20.0
30.0
40.0
50.0
60.0
70.0
80.0
90.0
100.0
110.0
120.0
130.0
140.0
150.0
160.0
170.0
180.0
190.0
200.0
210.0
220.0
230.0
240.0
250.0
260.0
73
28
75
77
63
51
111
89
91
99
264
120
121
105
101
102
115
127
138
165
191
220
219
14
15
29
39
50
74
76
78

## Supporting Information for Tables

# Synthesis, leishmanicidal, trypanocidal, antiproliferative assay and apoptotic induction of (2-phenoxypyridin-3-yl)naphthalen-1(2*H*)-one derivatives

**Table S1** Quantitative analysis of percent of apoptotic effect on human K562 cell line induced by compounds **18** and **22**

| Comp. | μM | K562 | | |
|---|---|---|---|---|
| | | AV+/IP- | AV+/IP+ | AV-/IP+ |
| **18** | 5 | 22.70 | 2.84 | 0.89 |
| **18** | 10 | 8.69 | 89.50 | 1.12 |
| **22** | 5 | 20.90 | 1.85 | 0.44 |
| **22** | 10 | 21.80 | 77.70 | 0.29 |
| **Dox** | 1 | 29.40 | 29.80 | 19.20 |
| **QC** | 50 | 34.30 | 6.25 | 1.57 |
| **Control** | | 4.63 | 2.00 | 1.60 |

Data are expressed as a percentage of the total cellular population of at least three determinations, **Dox** = doxorubicin, **QC** = quercetin, **Control** = untreated cells, **AV+=** Annexin-V Positive, **IP+=** Propidium iodide Positive, *p <0.05, **p <0.01 vs control

**Table S2** Quantitative analysis of percent of apoptotic effect on human A549 cell line induced by compounds **18** and **22**

| Comp. | μM | A549 | | |
|---|---|---|---|---|
| | | AV+/IP- | AV+/IP+ | AV-/IP+ |
| **18** | 10 | 11.10 | 3.28 | 3.06 |
| **18** | 25 | 40.10 | 21.10 | 3.12 |
| **22** | 10 | 13.80 | 23.30 | 8.44 |
| **22** | 25 | 9.36 | 78.30 | 9.32 |
| **Dox** | 1 | 8.33 | 33.50 | 29.30 |
| **QC** | 50 | 26.10 | 21.70 | 5.60 |
| **Control** | | 5.92 | 1.56 | 1.90 |

Data are expressed as a percentage of the total cellular population of at least three determinations, **Dox** = doxorubicin, **QC** = quercetin, **Control** = untreated cells, **AV+=** Annexin-V Positive, **IP+=** Propidium iodide Positive, *p <0.05, **p <0.01 vs control
